# Galápagos tortoise stable isotope ecology and the 1850s Floreana Island *Chelonoidis niger niger* extinction

**DOI:** 10.1038/s41598-022-26631-y

**Published:** 2022-12-23

**Authors:** Cyler Conrad, Laura Pagès Barceló, Lauren Scheinberg, Patrick D. Campbell, Addison Wynn, James P. Gibbs, Washington Tapia Aguilera, Linda Cayot, Kale Bruner, Allen G. Pastron, Emily Lena Jones

**Affiliations:** 1 Department of Anthropology, University of New Mexico, MSC01-1040, Anthropology 1, Albuquerque, NM 87131 USA; 2 Environmental Stewardship, Los Alamos National Laboratory, P.O. Box 1663, Los Alamos, NM J97887545 USA; 3 Department of Biology, University of New Mexico, MSC03-2020, Albuquerque, NM 87131 USA; 4 California Academy of Sciences, 55 Music Concourse Drive, Golden Gate Park, San Francisco, CA 94118 USA; 5 Darwin Centre, Natural History Museum, London, Cromwell Road, London, SW7 5BD England, UK; 6 Department of Vertebrate Zoology, National Museum of Natural History, Washington, DC 20560 USA; 7 Department of Environment and Forest Biology, State University of New York, 1 Forestry Drive, Syracuse, NY 13210 USA; 8 Galapagos Conservancy, 11150 Fairfax Boulevard, Suite 408, Fairfax, VA 22030 USA; 9 University of Málaga, Campus Teatinos, Apdo 59.29080, Málaga, Spain; 10 Museum of the Aleutians, 314 Salmon Way, Unalaska, AK 99685 USA; 11 Archeo-Tec: Consulting Archaeologists, 5283 Broadway, Oakland, CA 94618 USA

**Keywords:** Biochemistry, Ecology

## Abstract

A consequence of over 400 years of human exploitation of Galápagos tortoises (*Chelonoidis niger* ssp.) is the extinction of several subspecies and the decimation of others. As humans captured, killed, and/or removed tortoises for food, oil, museums, and zoos, they also colonized the archipelago resulting in the introduction of invasive plants, animals, and manipulated landscapes for farming, ranching, and infrastructure. Given current conservation and revitalization efforts for tortoises and their habitats, here we investigate nineteenth and twentieth century Galápagos tortoise dietary ecology using museum and archaeological specimens coupled with analysis of carbon (δ^13^C_collagen_ and δ^13^C_apatite_), nitrogen (δ^15^N), hydrogen (δD) and oxygen (δ^18^O_apatite_) stable isotopes and radiocarbon dating. We identify that Galápagos tortoise diets vary between and within islands over time, and that long-term anthropogenic impacts influenced change in tortoise stable isotope ecology by using 57 individual tortoises from 10 different subspecies collected between 1833 and 1967—a 134-year period. On lower elevation islands, which are often hotter and drier, tortoises tend to consume more C_4_ vegetation (cacti and grasses). Our research suggests human exploitation of tortoises and anthropogenic impacts on vegetation contributed to the extinction of the Floreana Island tortoise (*C. n. niger*) in the 1850s.

## Introduction

**Figure 1:**
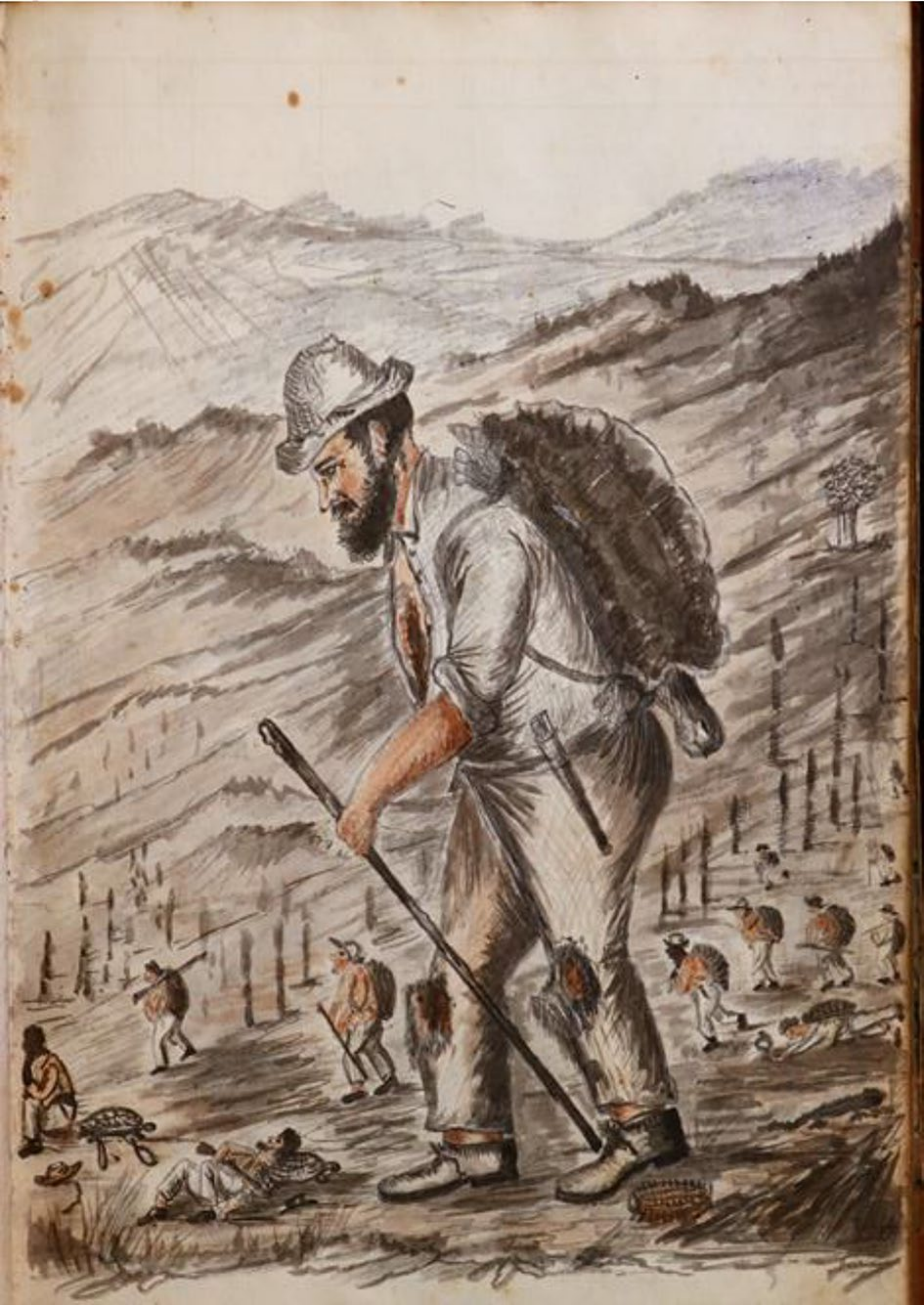
“Backing Terrapin from the Mountains on Chatham Island (San Cristóbal), Galápagos Group, in the Pacific Ocean.” Courtesy of the San Francisco Maritime National Historical Park, SAFR 19951: Sketchbook, Coast Views from the 1849 Voyage of the *Canton* to San Francisco, by D.F. Bradford. Archival photograph by Chris Wilhite.

Anthropogenic modification of the Galápagos Islands, including exploitation of Galápagos tortoises (*Chelonoidis niger* ssp.), began shortly after discovery of the archipelago in 1535. For approximately 400 years, until the establishment of the Galápagos National Park in 1959, human consumption of Galápagos tortoises, both deliberate and inadvertent introduction of exotic plants and animals, human colonization, and habitat modification occurred relatively uninhibited^1–3^. Pirates and whalers captured and consumed tortoises, while colonists attempted to farm and ranch the islands (Fig. 1; Refs.^4–7^). Whalers alone killed at a minimum over 10,000 tortoises; estimates suggest approximately 200,000 tortoises died due to human activities. Ultimately, this led to the extinction of at least three subspecies of Galápagos tortoise and the decimation of others^8^; (Table 1). At the same time, on many islands in the Galápagos the introduction of rats, dogs, pigs, goats, cattle, and a variety of domesticated agricultural plants and other herbaceous and woody taxa resulted in significant modification of tortoise habitats, loss of native vegetation, competition for food, and the introduced presence of exotic predators^9–11^. Vegetation regimes that had been present throughout the Holocene disappeared post-colonization as invasive plants flourished^12^. This combination of impacts created simultaneous, long-lasting effects: tortoises died through direct consumption from introduced carnivorous predators (including humans) and through indirect competition and loss of habitats and resources. These changes also affected Galápagos tortoise diets, by increasing competition for resources and shifting the baseline food web towards introduced, non-native plant types^13–18^. Today, Galápagos tortoise populations and habitats are undergoing long-term rehabilitation with continued efforts towards conservation and native plant restoration.

**Table 1:** Galápagos tortoise taxa per island, their status, and island elevation.

Taxon	Island (Volcano)	Maximum Island (Volcano) Elevation (m)	Status
*Chelonoidis niger abingdonii*	Pinta	777	Extinct
*Chelonoidis niger becki*	Isabela (Wolf)	1707	Living
*Chelonoidis niger microphyes*	Isabela (Darwin)	1330	Living
*Chelonoidis niger vandenburghi*	Isabela (Alcedo)	1129	Living
*Chelonoidis niger vicina*	Isabela (Cerro Azul)	1640	Living
*Chelonoidis niger guntheri*	Isabela (Sierra Negra)	1124	Living
*Chelonoidis niger phantasticus*	Fernandina	1476	Living
*Chelonoidis niger darwini*	Santiago	907	Living
*Chelonoidis niger duncanensis*	Pinzón	458	Living
*Chelonoidis niger porteri*	western Santa Cruz	864	Living
*Chelonoidis niger donfaustoi*	eastern Santa Cruz	864	Living
*Chelonoidis niger* ssp.	Santa Fe	200	Undescribed/Extinct
*Chelonoidis niger chathamensis*	San Cristóbal	730	Living (with Undescribed form)
*Chelonoidis niger niger*	Floreana	640	Extinct
*Chelonoidis niger hoodensis*	Española	206	Living

Throughout the nineteenth and early twentieth centuries, humans also removed Galápagos tortoises to zoos, private estates, and museum collections worldwide^4,19,20^. Tissue samples from the curated specimens of these animals provide, in effect, a time capsule of Galápagos tortoise ecological information. These legacy collections offer a window into the long-term impacts of anthropogenic colonization and tortoise habitat modification in the Galápagos (see^21,22^ for a similar approach for tortoise genetic relationships).

**Figure 2:**
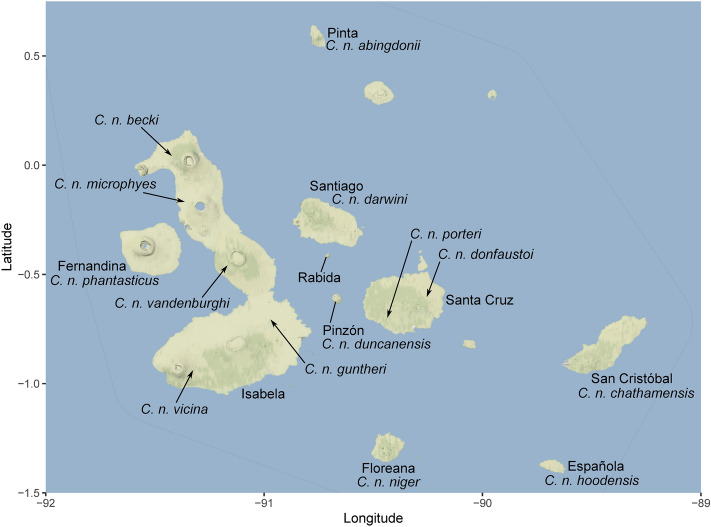
The Galápagos Islands and locations of tortoise subspecies.

In this paper, we investigate whether Galápagos tortoise diets vary between subspecies (i.e., distinct island populations; Fig. 2) as expected based on macro-dietary studies^15^, and if Galápagos tortoise diets shifted as a result of anthropogenic impacts, particularly on Floreana Island prior to the extinction of the endemic tortoise during the ~ 1850s. This approach, rooted in historical ecology (e.g.^23,24^), allows for an examination of baseline Galápagos tortoise dietary variation within and between islands prior to significant human impacts, and the scale and direction of human-derived dietary changes. We use carbon (δ^13^C_collagen_ and δ^13^C_apatite_), nitrogen (δ^15^N), hydrogen (δD), and oxygen (δ^18^O_apatite_) stable isotopes, and radiocarbon dating, of bone, skin, and scute curated by museums, and a single archaeological bone recovered from an 1850s archaeological context in San Francisco, California, ^25,26^ from ten Galápagos tortoise taxa to investigate tortoise dietary stable isotope ecology. We expect both subspecies-specific variation in tortoise diets, and an anthropogenically influenced shift in dietary ecology throughout the nineteenth and twentieth centuries.

## Results

**Figure 3:**
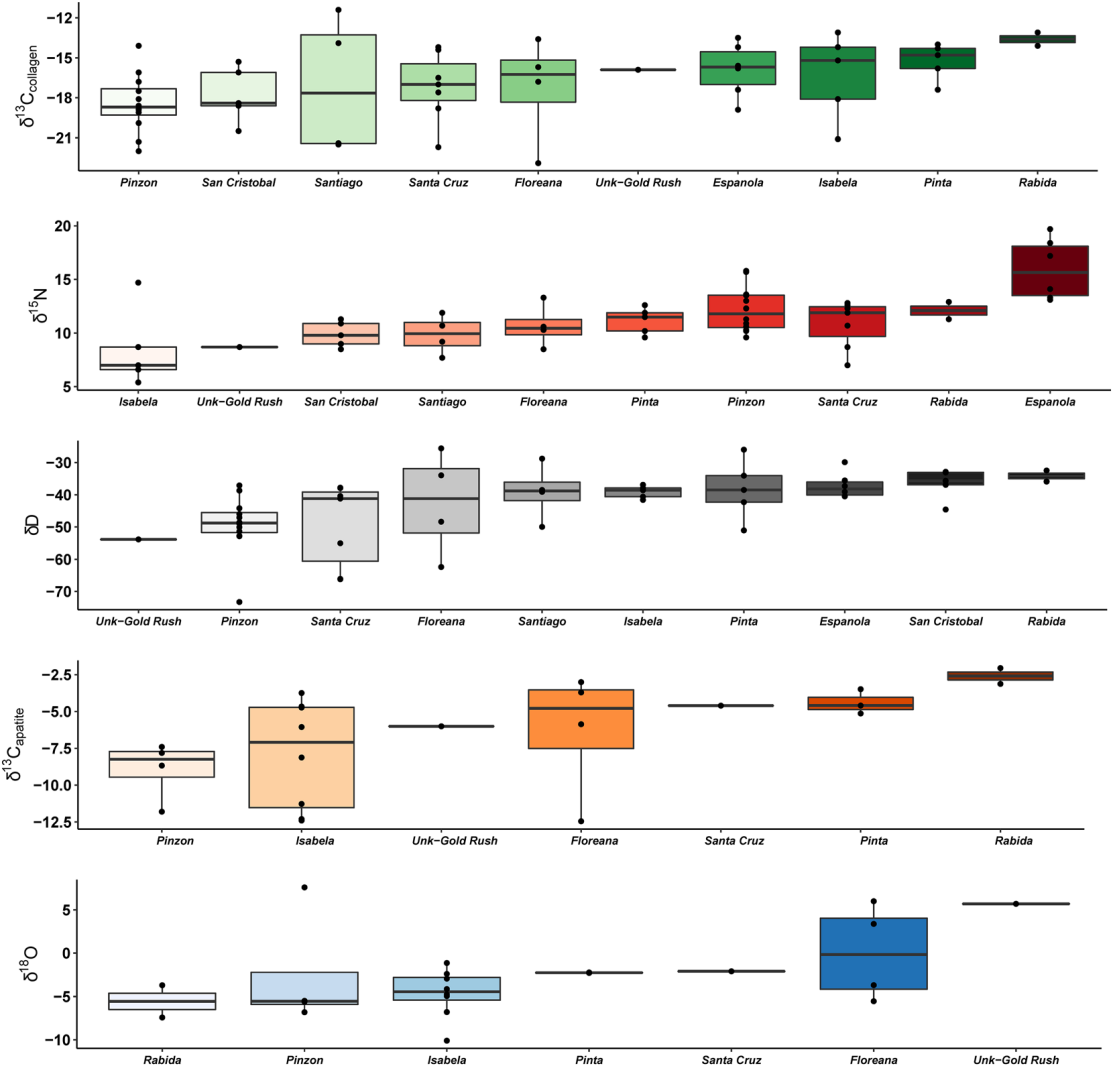
Bone collagen and apatite stable isotope boxplots, ranked by median isotope values and islands, for all wild Galápagos tortoises analyzed in this study.

**Figure 4:**
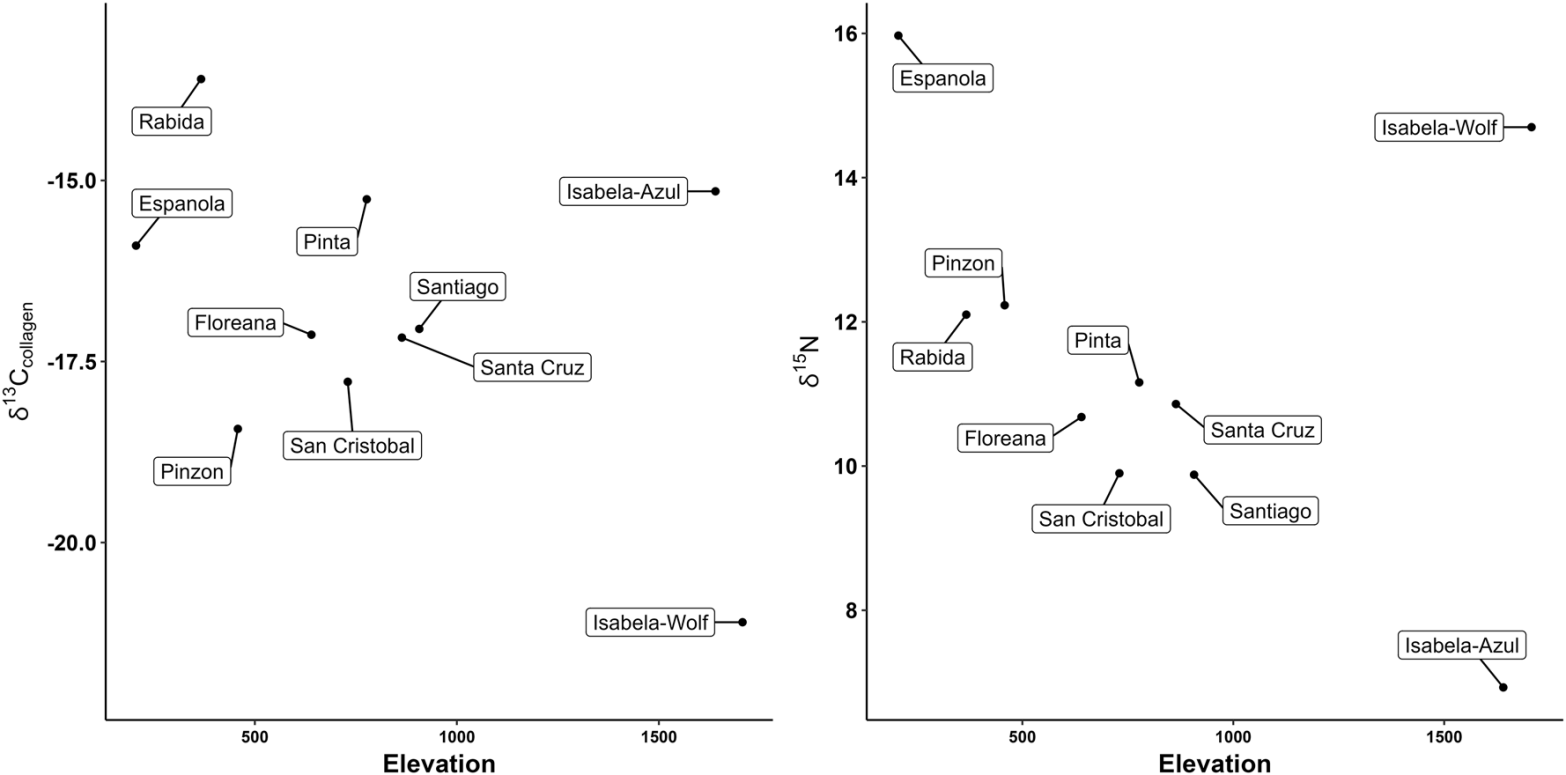
Average Galápagos tortoise stable isotope ratios for δ^13^C_collagen_ and δ^15^N per maximum island elevation (see Supplemental Information for additional results).

**Figure 5:**
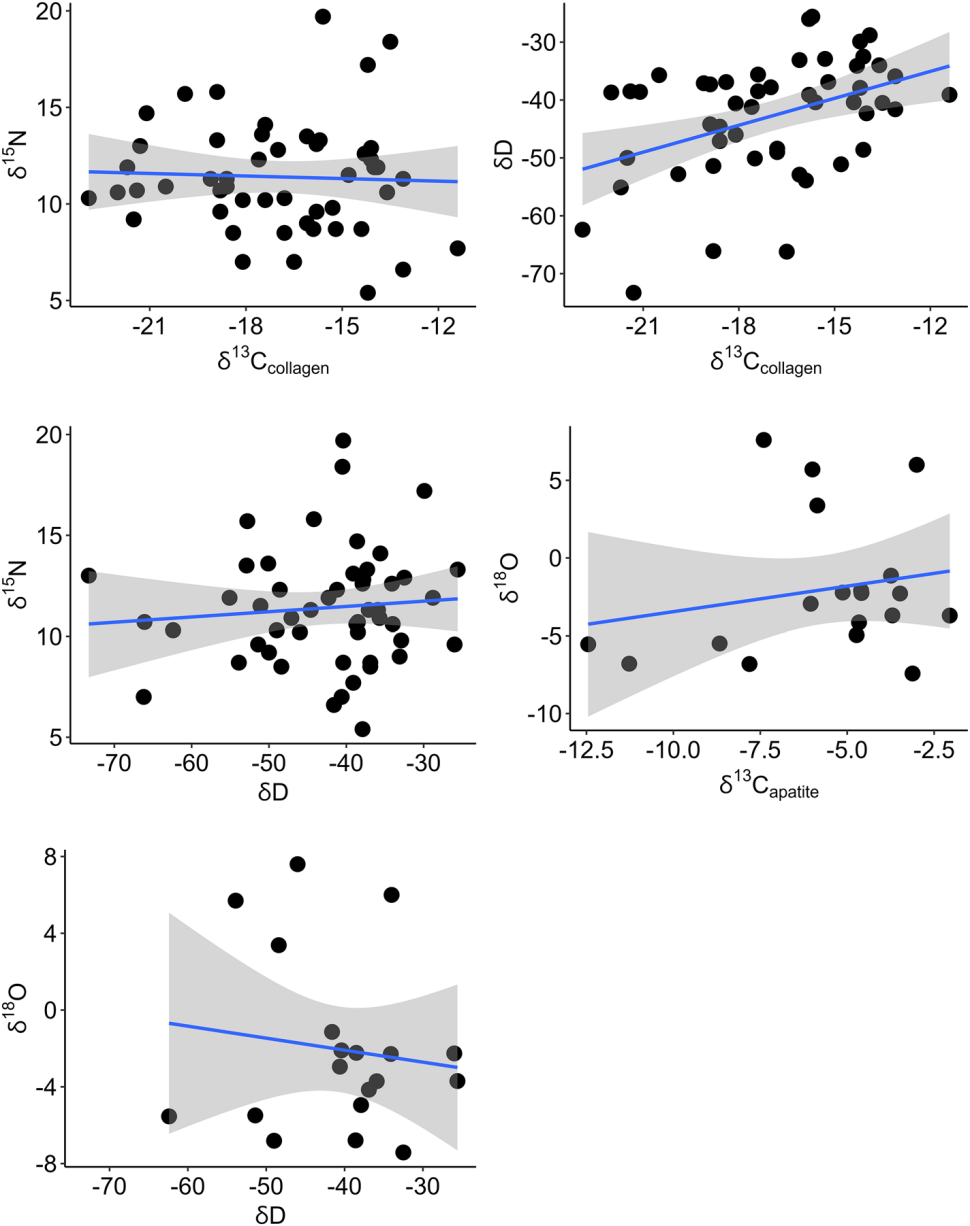
Relationship between stable isotope systems for all Galápagos tortoises analyzed in this study. See Supplemental Information for additional results.

Subspecies of Galápagos tortoise inhabiting distinct locations differ in their stable isotope signatures (Fig. 3, Table 2, Supplemental Table S1). For example, the Darwin Volcano tortoise (*C. n. microphyes*) on northern Isabela Island has a δ^13^C_collagen_ value of − 21.1‰ while the Sierra Negra Volcano tortoises (*C. n. guntheri*) on southern Isabela Island have a median value of − 14.7‰. Fluctuation within δ^15^N, δ^13^C_apatite_, δD and δ^18^O_apatite_ is equally variable between, and sometimes within islands. Qualitatively, changes in tortoise dietary ecology appear related to elevation and vegetation. Tortoises on the lower elevation islands exhibit enriched, average δ^13^C_collagen_ and δ^15^N ratios (Fig. 4), with higher elevation islands exhibiting depleted ratios. However, these patterns are less clear for δ^13^C_apatite_ and δD. Depleted δ^18^O_apatite_ ratios occur for tortoises on higher elevation islands (excluding tortoises transported to Rabida; see Supplemental Text, Figs. S1, S2, S3). Across all Galápagos tortoise subspecies, islands, and stable isotope systems, there exists a weak, significant, positive correlation between bone δ^13^C_collagen_ and δD (Fig. 5; adjusted R^2^ = 0.163, p < 0.05). No significant relationships exist between all other isotope systems (see Supplemental Figs. S4–S8).

**Table 2:** Summary metrics for Galápagos tortoise bone collagen and apatite stable isotopes.

	δ^13^C_collagen_	δ^15^N	δD	δ^13^C_apatite_	δ^18^O
Minimum	− 22.9	5.4	− 73.3	− 12.5	− 7.4
Mean	− 16.9	11.4	− 42.7	− 5.7	− 2.0
Median	− 16.8	11.3	− 40.4	− 4.7	− 3.0
Maximum	− 11.4	19.7	− 25.6	− 2.1	7.6

Plant δ^13^C and δ^15^N modeled against tortoise δ^13^C_collagen_ and δ^15^N across all samples and islands studied also suggests groupings based on the proportion of C_3_ and C_4_ inputs in tortoise diets (see^27^; Table 3; see Supplemental Table S2, Figs. S9, S10, S11). Española (*C. n. hoodensis*) and Pinta (*C. n. abingdonii*) tortoises consume a higher proportion of C_4_ plants, 77% and 81%, respectively, than tortoises inhabiting other islands. C_3_ plants comprise approximately 40–50% of tortoise diets on Floreana (*C. n. niger*), Isabela (multiple taxa), Pinzón (*C. n. duncanensis*) and Santiago (*C. n. darwini*).

**Table 3:** The proportion of C_3_ and C_4_ plants consumed by tortoises in this study, based on published plant stable isotope ratios^17^ (see Supplemental Table Supplemental Table S1 for source data), using the Stable Isotope Analysis in R package^26^.

Taxon	Island	SIAR Group	Proportion C_3_	Proportion C_4_	%C3	%C4
*C. n. hoodensis*	Española	1	0.2348441	0.7651559	23	77
*C. n. niger*	Floreana	2	0.4103207	0.5896793	41	59
*Chelonoidis n.* ssp. (Multiple)	Isabela	3	0.4196383	0.5803617	42	58
*C. n. abingdonii*	Pinta	4	0.1890172	0.8109828	19	81
*C. n. duncanensis*	Pinzón	5	0.4150514	0.5849486	42	58
*C. n. vicina*	Rabida	6	0.2632816	0.7367184	26	74
*C. n. chathamensis*	San Cristóbal	7	0.3788168	0.6211832	38	62
*C. n. porteri*	Santa Cruz	8	0.3328399	0.6671601	33	67
*C. n. darwini*	Santiago	9	0.4423766	0.5576234	44	56
*Chelonoidis n.* ssp.	Unk-Gold Rush	10	0.4619731	0.5380269	46	54

**Figure 6:**
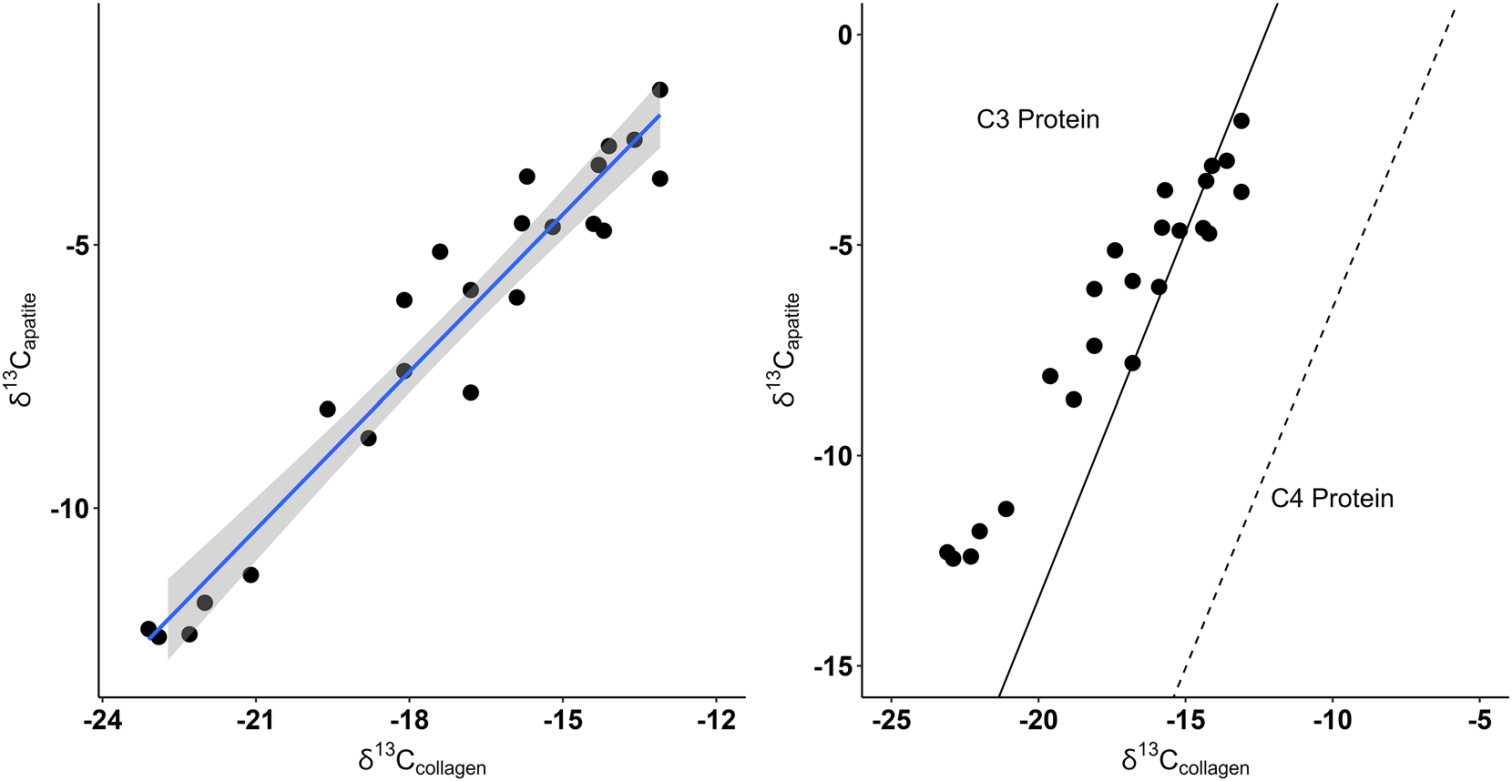
The relationship between δ^13^C_collagen_ and δ^13^C_apatite_ for individual Galápagos tortoises. C_3_ and C_4_ protein lines modeled after^28^.

Tissue-specific fractionation of δ^13^C within individual tortoises indicates that there is a significant relationship between δ^13^C_collagen_ and δ^13^C_apatite_ (Fig. 6; adjusted R^2^ = 0.93, p < 0.05). On average, δ^13^C_collagen_ is depleted − 10.6‰ compared to δ^13^C_apatite_. Comparison of possible tortoise protein sources suggests most Galápagos tortoises obtain protein through C_3_ resources. Fractionation of isotopes in various tortoise tissues indicates that the greatest shift occurs between bone apatite and bone collagen, with little to no change present for keratin and skin (see Supplemental Figs. S12–S14).

**Figure 7:**
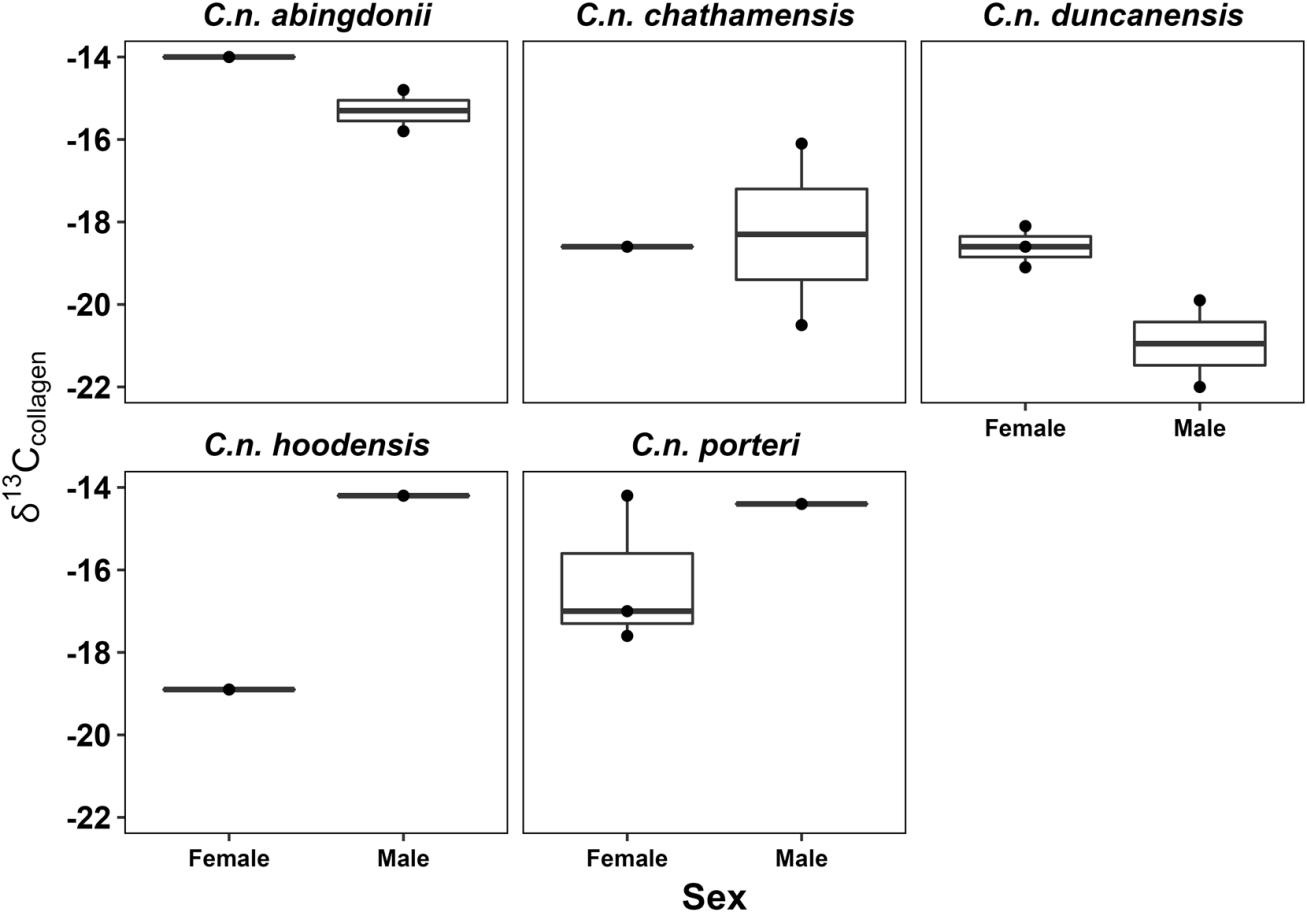
Sex based differences in δ^13^C_collagen_ for Galápagos tortoises on Pinta (*C. n. abingdonii*), San Cristóbal (*C. n. chathamensis*), Pinzón (*C. n. duncanensis*), Española (*C. n. hoodensis*) and Santa Cruz (*C. n. porteri*). Note: Unknown specimens are not plotted, see Supplemental Information for additional results.

As the museum specimens lack age information for all samples, we did not examine the impact of age on tortoise dietary ecology (although scute ring counting and/or skeletochronology techniques may provide a means of doing so in future studies). However, there do appear to be differences in male and female tortoise diets in some contexts. Male and female tortoises on Pinta and San Cristóbal Islands, for example, have similar median δ^13^C_collagen_ ratios, while Pinzón male tortoises are depleted compared to females. On both Española and western Santa Cruz (*C. n. porteri*), male tortoises are enriched relative to females (Fig. 7; see Supplemental Figs. S15–S18).

While several islands exhibit possible diachronic trends in stable isotopes (i.e., depletion in δ^13^C on Santa Cruz and Pinta over time), no clear patterns occur on Española, Rabida, San Cristóbal, Pinzón, Santiago, and Isabela (see Supplemental Figs. S19–S27). Only on Floreana Island is there a distinct record.

We analyzed four Floreana Island tortoises for stable isotopes (see Supplemental Text) with data for a fifth sample obtained from previously published literature^29^. The U.S. Navy killed two tortoises on Floreana in 1833 and their skeletons were donated to the Boston Natural History Society in 1834—Lord Walter Rothschild at the Natural History Museum at Tring eventually obtained one specimen, which is now housed in the Natural History Museum in London. This single 1833 specimen is one of the oldest tortoises in our sample (a second tortoise dating to 1833 in our analysis is from Santa Cruz Island but was not collected by the U.S. Navy). Three additional tortoises collected by Charles Townsend from a cave(s) on Floreana in 1928 date to 170 ± 30, 240 ± 30 and 880 ± 30 ^14^C years before present (BP), indicating they predate our 1833 specimen (Table 4; see also Supplemental Text). A final Floreana Island tortoise, also cave collected, was radiocarbon dated by Steadman and colleagues ^29^ to 310 ± 80 ^14^C years BP (with an associated δ^13^C_collagen_ value) and provides the fifth Floreana tortoise present in our analysis.

**Table 4:** Radiocarbon determinations for the Floreana Island tortoises collected from cave contexts in 1928.

Catalogue number	δ^13^C	Uncalibrated radiocarbon age	± sd	Laboratory Number	%C	%N	C:N
AMNH-46422	− 13.0	170	30	Beta-599427	40.63	14.79	3.2
AMNH-46424	− 15.6	880	30	Beta-599428	36.73	11.99	3.6
USNM-84294	− 17.3	240	30	Beta-599425	40.74	14.93	3.2

The δ^13^C value reported is from the radiocarbon preparation and analysis process.

**Figure 8:**
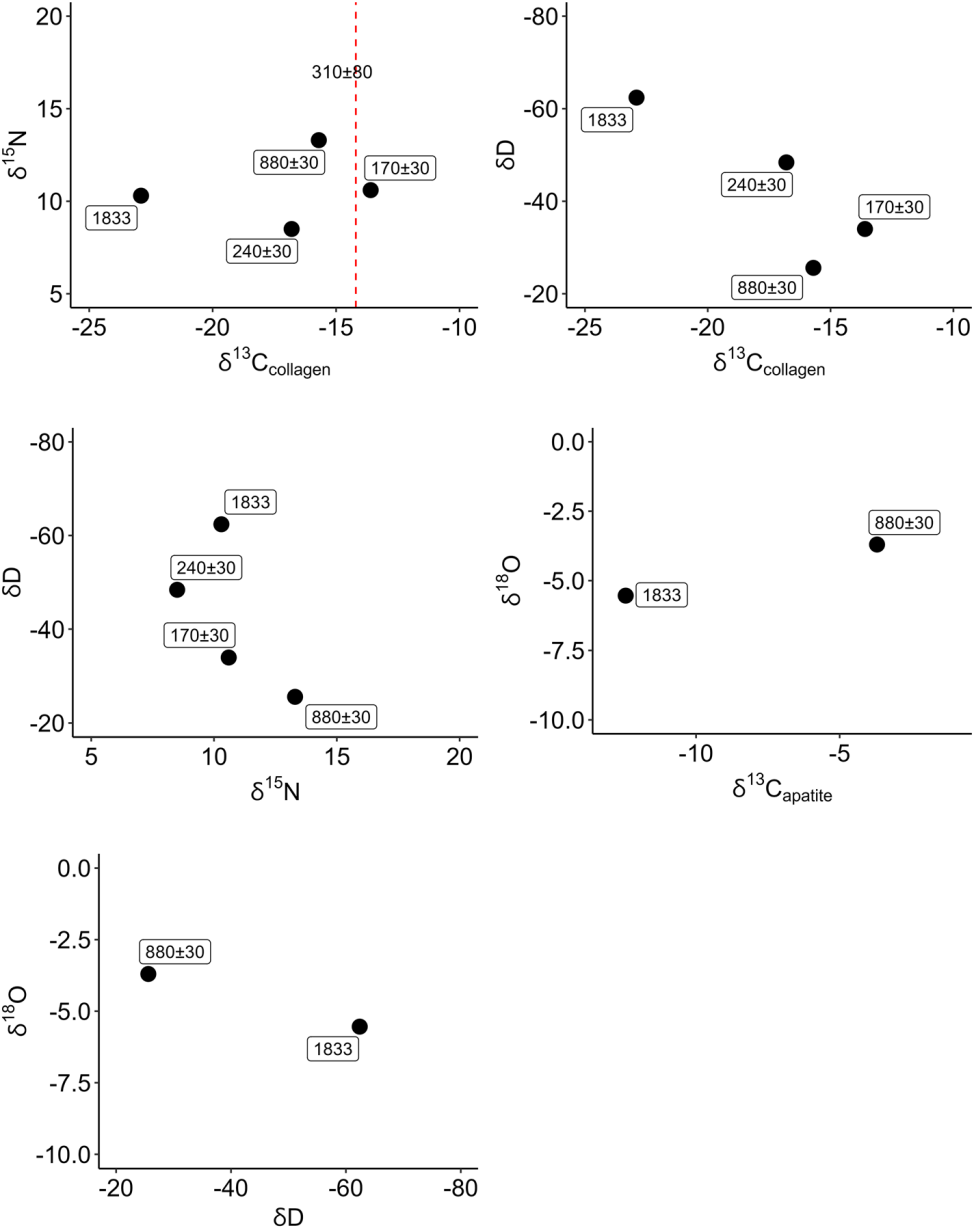
Diachronic trends in *C. n. niger* stable isotopes from Floreana Island. Red dashed line is the δ^13^C_collagen_ ratio from a tortoise analyzed in a separate study (see^29^).

Although our sample size is small—the lack of historically collected specimens likely relates to the decimation and eventual extinction of this taxon during the 1850s (see^30^)—the stable isotope systems examined here nonetheless indicate a critical shift in dietary ecology (Fig. 8). Enriched δ^13^C_collagen_ and δD occur in all samples predating 1833. Our single 1833 tortoise (− 22.9‰) shows an approximately 7‰ depletion in δ^13^C_collagen_ from the median value (− 15.7‰) predating this sample, and an approximately 28‰ depletion in δD from the median value (− 34‰).

## Discussion

Historic collections of Galápagos tortoises exhibit a range of dietary stable isotope values related to vegetation dynamics across the archipelago. These differences appear tied to island elevation and the relative aridity of tortoise foraging environments. Stable isotopes in Floreana Island tortoises provide evidence of anthropogenic impact prior to their extinction in the 1850s.

### Galápagos tortoise dietary ecology

Although tortoises analyzed in this study were collected prior to and during the early periods of anthropogenic landscape modification and non-native plant/animal introduction into the Galápagos, and thus are not perfectly comparable to modern tortoises and environments, they provide a critical baseline sample for tortoise macro-dietary studies. For example, in 1980, direct observations and fecal analysis of Alcedo Volcano (*C. n. vandenburghi*) tortoises indicated that grasses and sedges contributed to a large portion of their diet^31^. Observations noted that approximately 30–45% of *C. n. vandenburghi* diets included grasses and sedges (C_4_ plants) during the months of February-April, July and November, with consumption of *Sida* sp. browse between 22 and 28% of this time while foraging. When examining fecal matter, the proportion of *Sida* sp. increased compared to a decrease in the evidence for grasses and sedges (which likely relates to the effects of digestion on identifiable plant material). Tortoise diets at Volcán Alcedo tend to fluctuate with the dry and rainy seasons as well as by the availability of plant types along elevation gradients across the volcano throughout the year (see^32^). We did not analyze *C. n. vandenburghi* here, but we identified similar variability in a single Darwin Volcano tortoise (*C. n. microphyes*) and four Sierra Negra Volcano tortoises (*C. n. guntheri*) from Isabela dating to the late nineteenth and early twentieth centuries. Our *C. n. guntheri* specimens exhibit bone stable isotope ratios reflective of C_4_-plant consumption (median = − 14.7‰), but they contrast with a relatively depleted δ^13^C_collagen_ value for the single *C. n. microphyes* specimen (median =  − 21.1‰), a taxon which occupies a higher elevation volcano on Isabela. Dietary modeling of these analyzed Isabela tortoises suggests an approximately 58%-C_4_ to 42%-C_3_ proportion of plant types for consumption (Table 3). These observable and fecal matter dietary studies thus broadly match our stable isotope dietary values from historic specimens.

A similar analysis of tortoise diets from western (*C. n. porteri*) and eastern Santa Cruz (*C. n. donfaustoi*) populations between 2009 and 2013 identified that tortoises consumed at least 64 distinct plant taxa^14^. Over 30% of all feeding occurrences focused on grass-like plants, and when combined with forbs, these plants accounted for over 70% of recorded foraging activities. Santa Cruz tortoises therefore should theoretically consume a diet with a large proportion of C_4_ plants, and their stable isotope values support this conclusion with a median value of − 17.0‰ for δ^13^C_collagen_.

Modern dietary studies confirm that on low-elevation arid islands, such as Española and Pinzón, tortoises consume a different diet—with larger C_4_ inputs (cacti and grasses)—than do tortoises on higher-elevation islands, which are wetter and more humid^15^. Tortoises inhabiting these different island types also have differing carapace shapes (domed versus saddleback), which relate to diet (e.g., consumption of ground-level plants versus standing/reaching for food). Tortoise stable isotope values match this pattern, with individuals on lower-elevation islands displaying enriched δ^13^C_collagen_ and δ^15^N values, while individuals from higher-elevation islands have depleted δ^13^C_collagen_ and δ^15^N. For example, on Española Island (~ 200 m) tortoises exhibit median − 15.7‰ δ^13^C_collagen_ and 15.7‰ δ^15^N, while on Floreana Island (~ 640 m) tortoises exhibit median − 16.3‰ δ^13^C_collagen_ and 10.5‰ δ^15^N. Among tortoise populations that migrate across elevation gradients, sex-based dietary differences and consumption of differing diets during wet and dry seasons ultimately influence stable isotope values. Female and male tortoises also migrate for different reasons within certain islands and habitats, including for seasonal forage growth, or for finding nesting sites, and this likely influences dietary patterns.

### The Floreana Island Galápagos tortoise extinction

This dataset also allows for an investigation of long-term tortoise dietary change related to anthropogenic activities. Aside from direct human exploitation of tortoises for food, oil, and to fill private collections and museums around the world^4^, the most significant impacts on tortoise populations and habitats are introduced animals, plants, and anthropogenic landscape manipulation for agriculture and infrastructure^30^. During the 1830s and after a sequence of non-native animal introductions to the archipelago permanently altered Galápagos environments. These included goats, donkeys, cattle, pigs, rats, and dogs. The presence of these animals on the islands led, in some cases, to direct predation of tortoises and hatchlings; in others, it caused competition for resources and foraging areas. Similarly, introduction of invasive plants during the nineteenth and twentieth centuries impacted tortoise habitats (e.g., thickets of impenetrable vegetation that created barriers for tortoise mobility) but also provided new food types for tortoise consumption. The 2009–2013 study of tortoises on Santa Cruz identified that over 40% of tortoise foraging included non-native plants. Given these known impacts, our stable isotope data from Floreana Island (*C. n. niger*) is significant as it suggests the initiation of long-term vegetation change, and thus tortoise diets, prior to the extinction of this taxon during the ~ 1850s.

By the 1700s, whalers visiting the Galápagos to hunt sperm whales and exploit tortoises for food stopped on Floreana Island and its Post Office Bay where a wooden barrel served as a mailbox for passing vessels. Human settlement occurred by at least 1807 on Floreana, if not before, when an Irish sailor was abandoned on the island^30^. Following colonization and the introduction of non-native species, plant communities on Floreana changed dramatically: the arid environment of Floreana Island likely originally included a larger abundance of C_4_ (or crassulacean acid metabolism) plants, such as *Opuntia* sp. cacti; these plants were replaced by C_3_ trees and shrubs such as *Bursera graveolens*, a typical result of invasive animal foraging (see also^12,33^).

This process is visible in our results. Of the five samples analyzed here, the four cave collected tortoises from Floreana likely died through natural causes after falling into caves/crevices and becoming trapped (see^33^). Their radiocarbon ages indicate that these individuals lived through the era of human exploitation of tortoises, but not through significant landscape alteration on Floreana. The 1833 tortoise, however, would have experienced both human exploitation and landscape manipulation. A complex process of continuous tortoise exploitation by humans visiting Floreana, tortoise competition with introduced animals, C_4_-to-C_3_ vegetation change driven by non-native animal foraging, and human colonization of the island, would explain the difference in isotopic values between our 1833 specimen and the other four tortoises. The Floreana Island tortoise went extinct during the 1850s, likely due to the same combination of processes that caused the isotopic signature visible in our 1833 *C. n. niger* specimen.

### Natural history collections and Galápagos historical ecology

One of the tragedies of the legacy of human-tortoise interaction in the Galápagos is that when scientific interest in Galápagos tortoises increased, the history of human exploitation of these tortoises meant that several subspecies were already extinct while other populations were significantly depleted. In many cases, there was simply a lack of living tortoises, or deceased tortoise skeletal material, available to study certain populations. Charles Darwin regretted not collecting more tortoises, or keeping their skeletal remains, after tortoises were consumed on the *Beagle*, once he realized the potential of using individuals from different islands for his theory of evolution^34^.

The analyses presented here contribute to our understanding of anthropogenic impacts on tortoise populations during the nineteenth to twentieth centuries, but they also highlight the potential of natural history museum collections for research on Galápagos tortoises—and the need to seek out additional specimens (e.g.^22^). All of our studied *C. n. hoodensis* specimens, for example, derive from the 1905 to 1906 California Academy of Sciences expedition; it is possible, even likely, that earlier Española Island tortoises remain hidden within museum collections worldwide, waiting for genetic and radiocarbon analyses to confirm their speciation and provenience. While our analysis establishes a baseline of Galápagos tortoise stable isotope dietary ecology and the legacy of anthropogenic impacts on Floreana Island tortoises, only through continued and exhaustive investigation of tortoise skeletal collections will it be possible to reconstruct additional island-specific records.

## Materials and methods

### Sample procurement and data analysis

To establish a diachronic dataset of Galápagos tortoise dietary stable isotope ecology, we selected samples from five sources (see Supplemental Text): the American Museum of Natural History, New York, New York, (2) the California Academy of Sciences, San Francisco, California, (3) the Natural History Museum, London, England, (4) the National Museum of Natural History, Smithsonian Institution, Washington, D.C., and (5) the Thompson’s Cove (CA-SFR-186H) archaeological site in San Francisco, California. We provide details regarding sample provenience information and date-of-death as supplemental information. From these collections, we obtained single or multiple isotope samples from a total of 57 individual tortoises representing the following subspecies (n = 10) and islands: five *C. n. abingdonii* (Pinta Island), one *C. n. becki* (Volcán Wolf, Isabela Island), five *C. n. chathamensis* (San Cristóbal Island), four *C. n. darwini* (Santiago Island), thirteen *C. n. duncanensis* (Pinzón Island), four *C. n. guentheri* (Sierra Nega, *Isabela Island*), six *C. n. hoodensis* (Española Island), one *C. n. microphyes* (Volcán Darwin, Isabela Island), four *C. n. niger* (Floreana Island), nine *C. n. porteri* (Western Santa Cruz Island), one *C. n. vicina* (Cerro Azul, Isabela Island), one unknown Isabela Island tortoise, two *C. n. vicina* tortoises which were transported, lived and collected on Rabida Island, and one unknown tortoise (*Chelonoidis niger* ssp.; unknown Island—the San Francisco Gold Rush sample). The two earliest collected tortoises in our sample date to1833 and the latest tortoise is from 1967, representing a period of 134 years.

To understand tissue-specific isotopic variation and fractionation for the purposes of reconstructing long-term dietary ecology, we sampled tortoise bone collagen (n = 57), bone apatite (n = 23), scute keratin (n = 8) and skin (n = 2) for carbon (δ^13^C_collagen_ and δ^13^C_apatite_), nitrogen (δ^15^N), hydrogen (δD) and oxygen (δ^18^O_apatite_) stable isotopes. All samples were drilled or cut using a Dremel rotary tool with either a blade or diamond spherical bit attachment and were transported to the University of New Mexico, Center for Stable Isotopes (UNM-CSI), Albuquerque, NM, for preparation and analysis. All statistical and metric data analysis and visualization occurred in R (4.0.4) and RStudio (2022.02.4). We provide reproducible source code supplemental to the text^35^.

### Bone collagen δ^13^C, δ^15^N and δD

Analysis of bone collagen, skin and scute keratin for carbon, nitrogen and hydrogen stable isotopes followed standardized protocols (e.g., see^36^). For bone collagen, we cut and demineralized a small portion of bulk bone in 0.5 N hydrochloric acid (HCl) at 5 °C for 24 h prior to rinsing all samples to neutrality using deionized water. For lipid extraction, we immersed the samples in a solution of 2:1 chloroform:methanol (C_2_H_5_Cl_3_) for 24 h (repeated three times) while also sonicating samples for 15 min to ensure complete chemical saturation. Preparation of skin and scute keratin samples was only included this during the later lipid extraction step (i.e., no demineralization required). After 72 h we rinsed all samples to neutrality and lyophilized the tortoise samples for another 24 h. We then measured approximately 0.5–0.6 mg of bone collagen/skin/scute tissue into tin capsules for carbon (δ^13^C_collagen_) and nitrogen (δ^15^N) stable isotope analysis. We also measured approximately 0.2–0.3 mg of bone collagen/skin/scute tissue into silver capsules for hydrogen (δD) isotope analysis. We report isotope values in delta (δ) notation, calculated as: ((R_sample_/R_standard_) − 1) × 1000, where R_sample_ and R_standard_ are the ratios (e.g., ^13^C/^12^C, ^15^N/^14^N) of the unknown and standard material, respectively. Delta values are reported as parts per thousand (‰).

Carbon and nitrogen samples were measured on a Costech 4010 elemental analyzer (Valencia, California, USA) coupled to a Scientific Delta V Plus isotope ratio mass spectrometer by a Conflo IV, and hydrogen samples were measured on a Finnigan high-temperature conversion elemental analyzer (TC/EA) coupled to a Thermo Scientific Delta V Plus mass spectrometer by a Conflo IV at UNM-CSI (see^37^ for details on the high temperature conversion method for hydrogen analysis). All nitrogen and carbon isotope data are reported relative to atmospheric N_2_ and V-PDB, respectively. The data were corrected using lab standards with values of δ^15^ N = 6.4‰ and δ^13^C =  − 26.5‰ (casein protein), and of δ^15^N = 13.3‰ and δ^13^C =  − 16.7‰ (tuna muscle) that have been calibrated relative to the universally accepted standards: IAEA-N1, USGS 24, IAEA 600, USGS 63, and USGS 40.

To ensure equilibrium between the exchangeable hydrogen in tissue samples and local atmosphere^38^, we weighed hydrogen standards and samples into silver capsules and allowed both to sit in the laboratory for at least 2 weeks before analysis. Hydrogen data were corrected using three UNM-CSI laboratory keratin standards (δD_non-ex_ =  − 174‰, − 93‰, and − 54‰) of which the δD_non-ex_ values were previously determined through a series of atmospheric exchange experiments. These standards were also calibrated to USGS standards CBS and KHS values of − 178.8‰ and − 47.5‰, respectively (see^39,40^ for details and updated values). To quantitate any error imparted to our collagen data through correction with keratin standards, a UNM-CSI cow (*Bos taurus*) bone collagen standard was analyzed in every run over a 6-month period (July 2017–January 2018) and gave an inter-run standard deviation of 3.9‰, suggesting the difference in percent exchangeable hydrogen between collagen and keratin tissues did not significantly impact our results. All hydrogen isotope data are reported relative to Vienna-Standard Mean Ocean Water (V-SMOW). The H^3^ factor was between 8 and 8.5 for all runs.

Collagen precision (standard deviation; SD) for within-run analyses is < 0.14‰ for δ^13^C_collagen_ and δ^15^N, and ≤ 0.54‰ δD. Given that weight percent C:N ratios provide a measure of collagen contamination^41^, and our samples ranged between 2.7 and 3.5, this suggests intact and preserved collagen.

### Bone apatite δ^13^C and δ^18^O

To understand bone apatite (structural carbonate) carbon and oxygen stable isotopes we homogenized a small sample of tortoise bone powder for analysis following standardized protocols (e.g., see^36^). This homogenized powder was cleansed with 3% hydrogen peroxide (H_2_O_2_) for 24 h to remove organics and then was rinsed to neutrality using deionized water and centrifugation. A second treatment of 0.1 M buffered acetic acid (CH_3_COOH) for 30 min (followed by rinsing to neutrality) occurred to remove labile carbonates. After drying, we measured approximately 8.0–10.0 mg of homogenized bone apatite powder into exetainer vials, flushed those vials with He to remove atmospheric CO_2_, and then reacted the sample with phosphoric acid at 50 °C for at least 6 h. This reaction produced CO_2_ for carbon (δ^13^C_apatite_) and oxygen (δ^18^O_apatite_) stable isotope analysis. All samples were analyzed at UNM-CSI on a Thermo Scientific GasBench (Bremen, Germany) coupled to a Delta Plus isotope ratio mass spectrometer with a Conflo II. An in-house Carrara marble standard (δ^13^C = 2.0 and δ^18^O =  − 1.8) was analyzed in every run and used to correct the data. All data are reported relative to Vienna Pee Dee Belemnite (V-PDB). Within-run standard precision (SD) is < 0.25‰ for δ^13^C and δ^18^O.

### Radiocarbon

We submitted radiocarbon samples to Beta Analytic Testing Laboratory in Miami, Florida. Beta Analytic prepared sub-sampled bulk bone collagen specimens from AMNH-46422 (Beta-599427), AMNH-46424 (Beta-599428), and USNM-84294 (Beta-599425), using internationally accepted protocols prior to measurement on an Accelerator Mass Spectrometer. Specific details relating to sample preparation and results are available in the Supplemental Text. Radiocarbon determinations are standardized to the Libby 5568-year half-life.

### Stable isotope mixing model

Estimation of the proportion of C_3_ and C_4_ plants in Galápagos tortoise diets occurred through a Bayesian Monte Carlo fitted model using consumer (tortoises), sources (plants) and, and trophic enrichment factors (see^27,42^). Our dataset of non-corrected (see below) Galápagos tortoise δ^13^C and δ^15^N stable isotopes functioned as the consumer data for the model. We then extracted plant δ^13^C and δ^15^N from Gibbs et al.^17^. Collection of these modern plants occurred on three islands—Pinta, Santa Fe and Española (see Supplemental Table S2)—but we use these data as a proxy for vegetation stable isotope ratios across the archipelago and thus compare them against all tortoise subspecies. Plants within this dataset fall into two distinct groups (C_3_ and C_4_), and we take the average δ^13^C and δ^15^N ratios from these groups for our source input values. We obtained tortoise trophic enrichment factors from turtle controlled feeding studies^43,44^. Future such studies specific to Galápagos tortoises are required to fully evaluate these factors (see^45,46^).

### Suess corrections

Anthropogenic changes in atmospheric carbon (^13^CO_2_ to ^12^CO_2_) due to the burning of fossil fuels impact δ^13^C ratios; addressing these requires a Suess correction^47^. Given the short temporal range of most of our samples, in this study we apply the correction only to those samples pre-dating 1833, that is, the Floreana Island tortoise δ^13^C ratios. For all other tortoise and plant samples, we did not Suess correct the data, and it is presented in unaltered form (see Supplemental Table S1 for details). We note that for wild, non-captive tortoises, the greatest calendrical age in our samples is between 1833 (Floreana and Santa Cruz) and 1929–1934 (Santa Cruz), an approximately 100-year period, and the Suess correction between 1833 and 1934 is 0.3‰. As future research focuses on analysis of more recent Galápagos tortoises, it will be necessary to correct our ratios to account for changes between, for example, the nineteenth and twenty-first centuries.

## Supplementary Information

Supplementary Information 1.

Supplementary Information 2.

## Data Availability

All data and supplemental source code in R is available through an open-source repository: 10.17605/OSF.IO/ZMBV5.
