# Supplementary material for: Galápagos tortoise stable isotope ecology and the 1850s Floreana Island Chelonoidis niger niger extinction
Source: Sci Rep. 2022 Dec 23;12:22187. doi: 10.1038/s41598-022-26631-y (PMC9789057; doi:10.1038/s41598-022-26631-y)
Supplement: Supplementary file 2 — Supplementary Information 2. [file 41598_2022_26631_MOESM2_ESM.docx]

**Supplementary Information for**

Galápagos tortoise stable isotope ecology and the 1850s Floreana Island *Chelonoidis niger niger* extinction

Cyler Conrad, Laura Pagès Barceló, Lauren Scheinberg, Patrick D. Campbell, Addison Wynn, James P. Gibbs, Wacho Tapia Aguilera, Linda Cayot, Kale Bruner, Allen G. Pastron, and Emily Lena Jones

Supplementary Text

**Galápagos Tortoise Sample Provenience and History.**

Galápagos tortoise (*Chelonoidis niger* ssp.) samples analyzed during this project derive from five sources: 1) the American Museum of Natural History, New York, New York, 2) the California Academy of Sciences, San Francisco, California, 3) the Natural History Museum, London, England, 4) the National Museum of Natural History, Smithsonian Institution, Washington, D.C., and 5) the Thompson’s Cove (CA-SFR-186H) archaeological site in San Francisco, California.

The following provides a specimen-by-specimen discussion of tortoise provenience, sex, age, history, and date of death organized by our laboratory sample identifications (ID). In some cases, original institution records identify tortoises based on previously accepted taxonomic criteria (e.g., *Geochelone* vs. *Chelonoidis*). Therefore, for all specimens, we accept and include the most recently published taxonomic criteria [1]. This forms the basis of our specimen identification to both species and island in the Galápagos. For specimens which have previously published DNA, they are specifically indicated below.

Finally, as the date of when each tortoise was killed, collected, or died is significant for understanding diachronic trends in stable isotopes, we use the terms *terminus post quem* and *terminus ante quem* to indicate the earliest, and latest, moment of death for the tortoises, respectively. For example, during the 1905-1906 California Academy of Sciences expedition to the Galápagos numerous tortoises were collected and killed, but expedition members also collected several individual tortoise skeletal elements from caves (and elsewhere). These cave-collected tortoise bones gathered in 1905-1906 thus have a *terminus ante quem* date of 1905-1906, as they must have died before this date, but the true date of death is currently unknown (e.g., see Sample ID 3/ CAS-8138).

*Sample ID 1*

Taxon: *Chelonoidis* *niger* ssp.

Galápagos Island: Unknown

Collection: Thompson’s Cove (CA-SFR-186H), San Francisco, California

Sex: Unknown

Age: Unknown

Date: 1850s

Catalogue Number: TC-529

Notes: Zooarchaeological analysis identified two *Chelonoidis* *niger* ssp. specimens from the Thompson’s Cove archaeological site based on morphological comparisons between Galápagos tortoise collections curated at the California Academy of Sciences. One tortoise specimen is a left humerus shaft and distal end fragment (catalogue number TC-529) which likely articulates with the second specimen, a left humerus head fragment (TC-530) identified in the same context: Courtyard-Area 1/Block 2 (South of 40N) in Feature 25 [2]. Isotopic analyses discussed here derive from specimen TC-529. This sample dates to the 1850s [3].

*Sample ID 2*

Taxon: *Chelonoidis niger vicina*

Galápagos Island: Rabida Island

Collection: California Academy of Sciences

Sex: Unknown

Age: Adult

Date: December 20, 1905

Catalogue Number: CAS-8134

Notes: Catalogued in the California Academy of Sciences (CAS) collections as *Geochelone wallacei*. Although the CAS expedition to the Galápagos identified and collected this single tortoise from Rabida Island [4: 34], genetic analysis of this specimen indicates that this tortoise was likely transported to Rabida from Isabela Island [5: 167]. Here, we cluster this tortoise, genetically, with those from Isabela Island, but allow its dietary stable isotope signature to reflect Rabida.

*Sample ID 3*

Taxon: *Chelonoidis niger hoodensis*

Galápagos Island: Española Island

Collection: California Academy of Sciences

Sex: Unknown

Age: Unknown

Date: June 27, 1906

Catalogue Number: CAS-8138

Notes: Catalogued in the CAS collections as *Geochelone hoodensis*. On June 27, 1906, the CAS expedition found only one living tortoise [4: 65; see Sample ID 7] but reported collecting “some good bones”. Therefore, this specimen has a *terminus ante quem* death date of 1906.

*Sample ID 4*

Taxon: *Chelonoidis niger microphyes*

Galápagos Island: Isabela Island

Collection: California Academy of Sciences

Sex: Male

Age: Adult

Date: 1898

Catalogue Number: CAS-SUR 4800

Notes: Catalogued in the CAS collections as *Geochelone microphyes*. Although curated at the California Academy of Sciences, this Isabela Island tortoise originates from the Stanford University Galápagos expedition lead by E. Heller and R.E. Snodgrass in 1898-1899 [6]. However, documentation from this expedition indicates that *C. n. vicina* specimens *were not* collected from the Galápagos. Measurements of *C. n. vicina* are provided but the author writes, “The above table is derived from measurements made from *adult* living specimens taken by the crew. The specimens are not in the zoological museum of Stanford University” [emphasis in original; 6: 55]. Instead, the expedition reported collecting “the shells and skulls of four large males taken east of Tagus Cove,” likely from the species *C. n. microphyes*. Therefore, this specimen is considered an Isabela Island tortoise with a *terminus ante quem* death date of 1898.

*Sample ID 5*

Taxon: *Chelonoidis niger hoodensis*

Galápagos Island: Española Island

Collection: California Academy of Sciences

Sex: Unknown

Age: Unknown

Date: February 3, 1906

Catalogue Number: CAS-8136

Notes: Catalogued in the CAS collections as *Geochelone hoodensis*. No additional information identified.

*Sample ID 6*

Taxon: *Chelonoidis niger hoodensis*

Galápagos Island: Española Island

Collection: California Academy of Sciences

Sex: Unknown

Age: Unknown

Date: June 27, 1906

Catalogue Number: CAS-8137

Notes: Catalogued in the CAS collections as *Geochelone hoodensis*. On June 27, 1906, the expedition found only one living tortoise [4: 65; see Sample ID 7] but reported collecting “some good bones”. Therefore, this specimen has a *terminus ante quem* death date of 1906. See also, Sample ID 3.

*Sample ID 7*

Taxon: *Chelonoidis niger hoodensis*

Galápagos Island: Española Island

Collection: California Academy of Sciences

Sex: Male

Age: Adult

Date: June 27, 1906

Catalogue Number: CAS-8121 (Holotype)

Notes: Catalogued in the CAS collections as *Geochelone hoodensis*. E.S. King found this tortoise, “in the thick brush near the edge of a large open area” [4: 65].

*Sample ID 8*

Taxon: *Chelonoidis niger hoodensis*

Galápagos Island: Española Island

Collection: California Academy of Sciences

Sex: Female

Age: Adult

Date: June 26, 1906

Catalogue Number: CAS-8122 (Paratype)

Notes: Catalogued in the CAS collections as *Geochelone hoodensis*. W.H. Ochsner found this adult female tortoise “lying in the shade of a large cactus tree at the edge of a thick brush patch” [4: 65].

*Sample ID 9*

Taxon: *Chelonoidis niger porteri*

Galápagos Island: Santa Cruz Island

Collection: California Academy of Sciences

Sex: Female

Age: Adult

Date: 1897-1905

Catalogue Number: CAS-8276

Notes: Catalogued in the CAS collections as *Geochelone porteri*. On October 25, 1905, the California of Sciences expedition found two female, and one male, tortoises on Santa Cruz Island. The male tortoise is likely CAS-8275 (see Sample ID 11). However, there are three female tortoises collected on this date (CAS-8276, 8274 and 8382) in the collections, suggesting one of them must derive from tortoises previously killed on the Island by Rollo Beck in either 1897 or 1901. Therefore, the female specimens have a *terminus post quem* death date of 1897 and a *terminus ante quem* death date of 1905.

*Sample ID 10*

Taxon: *Chelonoidis niger porteri*

Galápagos Island: Santa Cruz Island

Collection: California Academy of Sciences

Sex: Female

Age: Adult

Date: 1897-1905

Catalogue Number: CAS-8274

Notes: Catalogued in the CAS collections as *Geochelone porteri*. See note for Sample ID 9. This specimen has a *terminus post quem* death date of 1897 and a *terminus ante quem* death date of 1905.

*Sample ID 11*

Taxon: *Chelonoidis niger porteri*

Galápagos Island: Santa Cruz Island

Collection: California Academy of Sciences

Sex: Male

Age: Adult

Date: October 26, 1905

Catalogue Number: CAS-8275

Notes: Catalogued in the CAS collections as *Geochelone porteri*. See note for Sample ID 9. Male tortoise captured on October 25, 1905, escaped, and recaptured and killed on October 26, 1905.

*Sample ID 12*

Taxon: *Chelonoidis niger porteri*

Galápagos Island: Santa Cruz Island

Collection: California Academy of Sciences

Sex: Female

Age: Unknown

Date: 1897-1905

Catalogue Number: CAS-8382

Notes: Catalogued in the CAS collections as *Geochelone porteri*. See note for Sample ID 9. This specimen has a *terminus post quem* death date of 1897 and a *terminus ante quem* death date of 1905.

*Sample ID 13*

Taxon: *Chelonoidis niger chathamensis*

Galápagos Island: San Cristóbal Island

Collection: California Academy of Sciences

Sex: Unknown

Age: Adult

Date: 410±20 years before present

Catalogue Number: CAS-8129

Notes: Catalogued in the CAS collections as *Geochelone chathamensis*. “Bones marked X (CAS 8129) from remains, all rotted away and no measurements could be taken” [4: 46]. This specimen has a *terminus ante quem* death date of 1906. Recent genetic analysis identified that this tortoise belongs to a now extinct clade on San Cristóbal Island, but it is not currently named as a new subspecies [7]. Therefore, we group the dietary stable isotope results from this tortoise with all other *C. n. chathamensis*. A single radiocarbon determination on this tortoise returned an age of 410±20 years before present [7].

*Sample ID 14*

Taxon: *Chelonoidis niger chathamensis*

Galápagos Island: San Cristóbal Island

Collection: California Academy of Sciences

Sex: Female

Age: Adult

Date: February 10, 1906

Catalogue Number: CAS-8133 (Paratype)

Notes: Catalogued in the CAS collections as *Geochelone chathamensis*. When this female tortoise was captured it was eating *Opuntia* cactus [4: 45]. Jensen and colleagues [7] genetically confirmed that this tortoise is *C. n. chathamensis*.

*Sample ID 15*

Taxon: *Chelonoidis niger abingdonii*

Galápagos Island: Pinta Island

Collection: California Academy of Sciences

Sex: Unknown

Age: Adult

Date: September 21, 1906

Catalogue Number: CAS-8113

Notes: Catalogued in the CAS collections as *Geochelone abingdoni*. On September 21, 1906, expedition members encountered a single male tortoise on Pinta Island (see Sample ID 18) and “an old shell and a few bones in a cave the tortoises probably fell into and died. We brought these down and they are in fairly good state” [4: 83]. Therefore, this specimen has a *terminus ante quem* death date of 1906.

*Sample ID 16*

Taxon: *Chelonoidis niger chathamensis*

Galápagos Island: San Cristóbal Island

Collection: California Academy of Sciences

Sex: Male

Age: Adult

Date: February 12-14, 1906

Catalogue Number: CAS-8127 (Holotype)

Notes: Catalogued in the CAS collections as *Geochelone chathamensis*. “Bones marked square (CAS 8127) belong to the large shell collected” [4: 46]. This specimen has a *terminus ante quem* death date of 1906. Recent genetic analysis identified that this tortoise belongs to a now extinct clade on San Cristóbal Island, but it is not currently named as a new subspecies [7]. Therefore, we group the dietary stable isotope results from this tortoise with all other *C. n. chathamensis*.

*Sample ID 17*

Taxon: *Chelonoidis niger chathamensis*

Galápagos Island: San Cristóbal Island

Collection: California Academy of Sciences

Sex: Unknown

Age: Adult

Date: February 12-14, 1906

Catalogue Number: CAS-8128

Notes: Catalogued in the CAS collections as *Geochelone chathamensis*. “Collected a good set of bones marked zero (CAS 8128)” [4: 45]. This specimen has a *terminus ante quem* death date of 1906. Recent genetic analysis identified that this tortoise belongs to a now extinct clade on San Cristóbal Island, but it is not currently named as a new subspecies [7]. Therefore, we group the dietary stable isotope results from this tortoise with all other *C. n. chathamensis*.

*Sample ID 18*

Taxon: *Chelonoidis niger abingdonii*

Galápagos Island: Pinta Island

Collection: California Academy of Sciences

Sex: Male

Age: Adult

Date: September 21, 1906

Catalogue Number: CAS-8112

Notes: Catalogued in the CAS collections as *Geochelone abingdoni*. On September 21, 1906, expedition members encountered this single male tortoise on Pinta Island “about a mile or two above the green zone on the southern slope of the mountain” [4: 83]. The stomach contents contained grass and cactus.

*Sample ID 19*

Taxon: *Chelonoidis niger hoodensis*

Galápagos Island: Española Island

Collection: California Academy of Sciences

Sex: Unknown

Age: Unknown

Date: September 27, 1905

Catalogue Number: CAS-8126

Notes: Catalogued in the CAS collections as *Geochelone hoodensis*. “[F]ragments of tortoise bones laying on the ground among the lava blocks and exposed to the sun,” which were collected by Rollo Beck from Española Island. This specimen has a *terminus ante quem* death date of 1905.

*Sample ID 20*

Taxon: *Chelonoidis niger vicina*

Galápagos Island: Rabida Island

Collection: California Academy of Sciences

Sex: Unknown

Age: Adult

Date: December 21, 1905

Catalogue Number: CAS-8135

Notes: Catalogued in the CAS collections as *Geochelone wallacei*. See Sample ID 2. The expedition identified and collected a single tortoise from Rabida Island [4: 34] on December 20, 1905, but there are no records of tortoises collected from Rabida on December 21, 1905. There is a record from this date indicating, “[f]inished skinning a turtle started yesterday and skinned one other [sic],” however, this is likely describing green sea turtles as they were being simultaneously collected and killed from Rabida during this time. Given that genetic analysis on the prior Rabida specimen indicates that the tortoise was transported to Rabida from Isabela Island [5: 167], and since no new tortoises are reported as collected from Rabida on this date, here we assume that CAS-8135 and CAS-8134 (Sample ID 2) are from the same individual or from two tortoises collected on the island. Therefore, we cluster this tortoise, genetically, with those from Isabela Island, but allow its dietary stable isotope signature to reflect Rabida.

*Sample ID 21*

Taxon: *Chelonoidis niger duncanensis*

Galápagos Island: Pinzón Island

Collection: California Academy of Sciences

Sex: Female

Age: Adult

Date: December 1-17, 1905

Catalogue Number: CAS-8376

Notes: Catalogued in the CAS collections as *Geochelone ephippium*. Unclear provenience. According to written records, no tortoises were collected or killed on Pinzón Island on December 1, 1905. However, at least 12 tortoises were killed from the Island during December 1-17, 1905 [4; including CAS-8342 and CAS-8343; Sample IDs 22 and 23]. Therefore, these tortoises are assumed to derive from kills during this period.

*Sample ID 22*

Taxon: *Chelonoidis niger duncanensis*

Galápagos Island: Pinzón Island

Collection: California Academy of Sciences

Sex: Female

Age: Adult

Date: December 1-17, 1905

Catalogue Number: CAS-8342

Notes: Catalogued in the CAS collections as *Geochelone ephippium.* See Sample ID 21 note.

*Sample ID 23*

Taxon: *Chelonoidis niger duncanensis*

Galápagos Island: Pinzón Island

Collection: California Academy of Sciences

Sex: Male

Age: Adult

Date: December 1-17, 1905

Catalogue Number: CAS-8343

Notes: Catalogued in the CAS collections as *Geochelone ephippium*. See Sample ID 21 note.

*Sample ID 24*

Taxon: *Chelonoidis niger guentheri*

Galápagos Island: Isabela Island

Collection: California Academy of Sciences

Sex: Female

Age: Adult

Date: August 1-September 4, 1906

Catalogue Number: CAS-8265

Notes: Catalogued in the CAS collections as *Geochelone guentheri.* Throughout this period, expedition members collected several tortoises from Isabela Island. Although this exact individual has unknown provenience, it was likely killed during this period in 1906.

*Sample ID 25*

Taxon: *Chelonoidis niger guentheri*

Galápagos Island: Isabela Island

Collection: California Academy of Sciences

Sex: Female

Age: Adult

Date: October 1906

Catalogue Number: CAS-8259

Notes: Catalogued in the CAS collections as *Geochelone guentheri.* Between late-September and mid-October 1906, expedition members collected and killed several tortoises from Isabela Island. Although records are unclear as to the exact provenience of this tortoise, and others (CAS-8251 and 8225; Sample IDs 26 and 27), it died during October 1906.

*Sample ID 26*

Taxon: *Chelonoidis niger guentheri*

Galápagos Island: Isabela Island

Collection: California Academy of Sciences

Sex: Female

Age: Adult

Date: October 1906

Catalogue Number: CAS-8251

Notes: Catalogued in the CAS collections as *Geochelone guentheri.* See Sample ID 25 note.

*Sample ID 27*

Taxon: *Chelonoidis niger guentheri*

Galápagos Island: Isabela Island

Collection: California Academy of Sciences

Sex: Female

Age: Adult

Date: October 1906

Catalogue Number: CAS-8225

Notes: Catalogued in the CAS collections as *Geochelone guentheri.* See Sample ID 25 note.

*Sample ID 28*

Taxon: *Chelonoidis niger darwini*

Galápagos Island: Santiago Island

Collection: California Academy of Sciences

Sex: Unknown

Age: Unknown

Date: December 28, 1905

Catalogue Number: CAS-8102

Notes: Catalogued in the CAS collections as *Geochelone darwini.* “Stayed on board and skinned tortoises,” is recorded in the entry for December 28, 1905 [4: 37].

*Sample ID 29*

Taxon: *Chelonoidis niger darwini*

Galápagos Island: Santiago Island

Collection: California Academy of Sciences

Sex: Unknown

Age: Unknown

Date: December 27, 1905

Catalogue Number: CAS-8103

Notes: Catalogued in the CAS collections as *Geochelone darwini*. “King and the Captain skinned tortoises today,” is recorded in the entry for December 27, 1905 [4: 36].

*Sample ID 30*

Taxon: *Chelonoidis niger darwini*

Galápagos Island: Santiago Island

Collection: California Academy of Sciences

Sex: Unknown

Age: Adult

Date: August 4, 1906

Catalogue Number: CAS-8104

Notes: Catalogued in the CAS collections as *Geochelone darwini*. In August 1906, the expedition recorded capturing and killing several tortoises from Santiago Island [4: 71-75]. Although it is unclear which specific tortoise this specimen is referenced to, it was killed and collected during this period.

*Sample ID 31*

Taxon: *Chelonoidis niger darwini*

Galápagos Island: Santiago Island

Collection: California Academy of Sciences

Sex: Female

Age: Adult

Date: August 3, 1906

Catalogue Number: CAS-8106

Notes: Catalogued in the CAS collections as *Geochelone darwini*. See Sample ID 30 note.

*Sample ID 32*

Taxon: *Chelonoidis niger niger*

Galápagos Island: Floreana Island

Collection: American Museum of Natural History

Sex: Unknown

Age: Unknown

Date: 170±30 radiocarbon years before present

Catalogue Number: AMNH-R46422

Notes: Catalogued in the American Museum of Natural History (AMNH) collections as *Geochelone elephantopus galapagoensis* collected in 1928. During the 1928 New York Zoological Society and U.S. Bureau of Fisheries expedition to the Galápagos, approximately 180 live tortoises were collected, removed and relocated from Isabela Island to locations throughout North America and elsewhere [8]. Several tortoises became included in the collections of the AMNH. However, while only Isabela tortoises were collected live, this expedition also noted, “[finding] in a cave a dozen large and fairly complete skeletons of the long-extinct tortoise of Charles Island” [8: 30]. It is important to note that Townsend [8: 157-159] indicates they specifically collected more recently deceased specimens (i.e., closer in death date to 1928) based on their appearance, “[t]he bleached and bony remains of those not too antiquated and fragile to be removed, had long lost their dark horny plates which lay curled and twisted beside them. In a dozen of these, both carapace and plastron were practically intact, while skulls and leg bones had usually been disturbed and scattered. A considerable amount of broken tortoise remains had long since become mixed with the soil of the cave floor. The later arrivals lay where they died, their large white carapaces showing conspicuously as our flashlights were turned in their direction [sic].” AMNH catalogue notes for this specimen also indicate “miscellaneous parts of many specimens,” confirming this is a tortoise sample collected from the cave on Floreana Island. This sample received bone radiocarbon dating (see text) and dates to 170±30 radiocarbon years before present.

*Sample ID 33*

Taxon: *Chelonoidis niger niger*

Galápagos Island: Floreana Island

Collection: American Museum of Natural History

Sex: Unknown

Age: Unknown

Date: 880±30 radiocarbon years before present

Catalogue Number: AMNH-R46424

Notes: Catalogued in the AMNH collections as *Geochelone nigra nigra* collected in 1928. See Sample ID 32 note. DNA from this specimen confirms the species identification as *C. n. niger* [5]. AMNH catalogue notes for this specimen note, “limb bones, vertebrae of many specimens,” confirming this is a tortoise sample collected from the cave on Floreana Island. This sample received bone radiocarbon dating (see text) and dates to 880±30 radiocarbon years before present.

*Sample ID 34*

Taxon: *Chelonoidis niger duncanensis*

Galápagos Island: Pinzón Island

Collection: American Museum of Natural History

Sex: Unknown

Age: Unknown

Date: September 1961

Catalogue Number: AMNH-R87330

Notes: Catalogued in the AMNH collections as *Testudo elephantopus ephippium* collected in 1928. According to records from the 1928 Galápagos expedition [8-9] no living tortoises, or tortoise bones, were collected from Pinzón [Duncan] Island during this time. However, in 1928 William K. Vanderbilt led an expedition to the Galápagos and collected five tortoises from Pinzón [10: 124; 8: 155]. Townsend writes, “[t]he tortoise of Duncan Island (*Testudo ephippium*) is well represented in museum collections and the New York Zoological Park has three adult specimens – the gift of Mr. Vanderbilt” [see 8: 154-155]. AMNH catalogue records for this specimen indicate that it was skeletonized in September 1961, after death. Therefore, this specimen (AMNH-R87330) was collected in 1928 and died in 1961 after living at the New York Zoological Park. This *C. n. duncanensis* specimen has a death date of 1961 and its stable isotope data does not reflect dietary ecology of the Galápagos, but rather a captive-fed diet.

*Sample ID 35*

Taxon: *Chelonoidis niger* ssp.

Galápagos Island: Isabella Island

Collection: American Museum of Natural History

Sex: Male

Age: Unknown

Date: November 1964

Catalogue Number: AMNH-R93382

Notes: Catalogued in the AMNH collections as *Geochelone e. elaphantopus* collected in 1928. See Sample ID 32 note. This *C. niger* ssp. specimen has a death date of 1964 as it lived in the New York Zoological Park until November 1964 when it died and was skeletonized. This male weighed 490 pounds at the time of death. Given that *C. niger* *niger* went extinct in the mid-19^th^ century, it is unclear which tortoise species this individual represents – but the living collection of tortoises from Isabella Island in 1928 (see Sample ID 32) suggests this is an Isabella Island tortoise. An additional note in the AMNH collections indicates that this tortoise was collected from “Isabela Island”, so it is likely that the listed species identification is inaccurate based on current taxonomies. This tortoise is grouped with Isabella Island tortoises in this analysis.

*Sample ID 36*

Taxon: *Chelonoidis niger becki*

Galápagos Island: Isabela Island

Collection: American Museum of Natural History

Sex: Unknown

Age: Unknown

Date: October 1967

Catalogue Number: AMNH-R110443

Notes: Catalogued in the AMNH collections as *Geochelone elaphantopus becki*. Provenience information for this specimen is unclear, but AMNH catalogue records clearly state this tortoise died in October 1967. During the early 1960s the herpetologist Herndon G. Dowling was employed at the AMNH as a research associate (i.e., an unpaid honorary non-staff position). One of the tortoises in the AMNH collections is a specimen attributed to Dowling from April 1962. This matches records of when Dowling surveyed tortoise populations in the Galápagos in 1962 [11]. This specimen (Sample ID 36) collected during June 1962 is also likely a Dowling collected tortoise [see also 12-13], but it survived in the United States until it died in October 1967. Thus, this *C. n. becki* specimen has a death date of 1967 and its stable isotope data does not reflect dietary ecology of the Galápagos, rather a captive-fed diet.

*Sample ID 37*

Taxon: *Chelonoidis niger duncanensis*

Galápagos Island: Pinzón Island

Collection: National Museum of Natural History

Sex: Unknown

Age: Unknown

Date: 1889

Catalogue Number: USNM 15192

Notes: Catalogued in the National Museum of Natural History (USNM) collections as *Geochelone elephantopus ephippium*. According to Smithsonian records, this tortoise was obtained in April 1888 from the “Galápagos Arch.” during the 1887-1888 Albatross Expedition to the Galápagos. In 1888 the first *Albatross* expedition to the Galápagos occurred [8, 14-15] and was followed by a second *Albatross* expedition in 1891 [16]. There is some discrepancy from the records of tortoises collected during the 1888 *Albatross* voyage, but “about 20 tortoises,” [14] or “eighteen of the long-necked and hard-shelled ‘Galápagos’ [sic]”, [15] or “sixteen tortoises, some of them from Abingdon Island” [8] were collected from the archipelago in 1888. An official account describes capturing “[t]en Galápagos of moderate size” from “Duncan Island” (Pinzón Island) and “eight tortoises” from “Chatham Island” (San Cristóbal) for a total of 18 during the first *Albatross* expedition [17: 39]. However, USNM collections also include two tortoises from Albemarle Island (Isabela Island) and one from Abingdon Island (Pinta), all collected in 1888 that are not described in the official *Albatross* account. Descriptions of these tortoises also indicate that they did not survive their first winter in Washington D.C. and are now “in the National Museum” [15] after being catalogued on March 30, 1889. Although USNM records do not indicate which island this tortoise was obtained, DNA analysis from this specimen confirms the species identification as *C. n. duncanensis* [5]. Therefore, we accept the species/island identification and date of death for this specimen as 1889 with a stable isotope record that likely reflects dietary ecology in the Galápagos.

*Sample ID 38*

Taxon: *Chelonoidis niger duncanensis*

Galápagos Island: Pinzón Island

Collection: Smithsonian National Museum of Natural History

Sex: Unknown

Age: Unknown

Date: 1889

Catalogue Number: USNM 15193

Notes: Catalogued in the USNM collections as *Geochelone elephantopus ephippium*. See Sample ID 37 note. DNA from this specimen also confirms the species identification as *C. n. duncanensis* [5].

*Sample ID 39*

Taxon: *Chelonoidis niger vicina*

Galápagos Island: Isabela Island

Collection: National Museum of Natural History

Sex: Unknown

Age: Unknown

Date: 1923

Catalogue Number: USNM 65896

Notes: Catalogued in the USNM collections as *Geochelone elephantopus vicina.* Smithsonian records for USNM 65896 do not record an exact provenience or date of collection, however, an associated note included with this specimen states, “Found in the attic and cleaned by Mr. Mirguet”. This is an important detail as Mr. Charles E. Mirguet worked at the Smithsonian sometime between 1892 and 1952 [18], which matches the catalogue date for this specimen of June 25, 1923. Therefore, we assume this *C. n. vicina* specimen has a *terminus ante quem* death date of 1923 and its stable isotope data does not reflect dietary ecology of the Galápagos, rather a captive-fed diet or unknown diet.

*Sample ID 40*

Taxon: *Chelonoidis niger niger*

Galápagos Island: Floreana Island

Collection: National Museum of Natural History

Sex: Unknown

Age: Unknown

Date: 240±30 radiocarbon years before present

Catalogue Number: USNM 84294

Notes: Catalogued in the USNM collections as *Geochelone elephantopus.* See Sample ID note 32. Smithsonian records indicate that this tortoise was collected during the 1928 *Albatross* Galápagos expedition. Although this voyage did not collect and/or kill living tortoises from Floreana Island, they did discover several recently deceased tortoises and their decomposing bones and tissues in a cave [8-9, 19]. This sample received bone radiocarbon dating (see text) and dates to 240±30 radiocarbon years before present.

*Sample ID 41*

Taxon: *Chelonoidis niger abingdonii*

Galápagos Island: Pinta Island

Collection: National Museum of Natural History

Sex: Unknown

Age: Unknown

Date: 1888.

Catalogue Number: USNM 222479

Notes: Catalogued in the USNM collections as *Geochelone elephantopus abingdoni.* See Sample ID 37 note. Expedition members noted that “[o]n the voyage of 1888 we obtained sixteen tortoises, some of them from Abingdon Island where they have since become extinct. This species is (*Testudo abingdoni*)” [8]. USNM records indicate that this is a “[u]nique spec” and it was catalogued on March 11, 1890. Note, there is no published DNA for this specimen.

*Sample ID 42*

Taxon: *Chelonoidis niger duncanensis*

Galápagos Island: Unknown – Possibly Pinzón Island

Collection: National Museum of Natural History

Sex: Unknown

Age: Unknown

Date: Unknown – Possibly 1928?

Catalogue Number: USNM 222494

Notes: Catalogued in the USNM collections as *Geochelone elephantopus ephippium*. See Sample ID note 37. Note, there is no published DNA for this specimen. Although Smithsonian records do not record exact provenience or collection information for this specimen, it is most likely that it derives from the Pinzón Island tortoises collected during the 1888 expedition to the Galápagos or from the 1928 Vanderbilt expedition [8]. Therefore, we ascribe a *terminus ante quem* death date of 1928.

*Sample ID 43*

Taxon: *Chelonoidis niger porteri*

Galápagos Island: Unknown – Possibly Santa Cruz Island?

Collection: National Museum of Natural History

Sex: Unknown

Age: Unknown

Date: Unknown – Possibly 1929-1934?

Catalogue Number: USNM 222501

Notes: Catalogued in the USNM collections as *Geochelone elephantopus nigrita*. Unfortunately, there are no associated catalogue data included with this tortoise specimen to indicate a date of death, collection or exact provenience in the Galápagos. Historic records indicate that at least three expeditions to Santa Cruz Island occurred in the late 1920s and early 1930s in which tortoises were collected and several brought to the USNM – the Pinchot expedition in 1929 [20], the Astor expedition in 1930 [21] and the Hancock expedition between 1931-1934 [22-23]. USNM records indicate that there are four tortoises from the Pinchot expedition (two from Isabela and two from Pinzón), but none from Santa Cruz Island – even though Pinchot records indicate collecting a single tortoise from the Island [20]. In contrast, at least eight tortoises from the Hancock expedition are from Santa Cruz, and these date to 1934 in the USNM collections (USNM collections include at least three individuals). The Astor expedition collected tortoises from Santa Cruz [10], but these likely ended up in the AMNH instead of the USNM. A recent review of collections from the Galápagos suggests that these were the only documented collecting trips on Santa Cruz in which tortoises could have been deposited at the Smithsonian [24-25]. Therefore, while the exact provenience is unknown, it is likely that this USNM specimen derives from either the Pinchot or Hancock expeditions and dates between 1929-1934. We assume the stable isotope data for this specimen reflects dietary ecology in the Galápagos.

*Sample ID 44*

Taxon: *Chelonoidis niger abingdonii*

Galápagos Island: Pinta Island

Collection: Natural History Museum, London

Sex: Female

Age: Unknown

Date: 1875

Catalogue Number: NHMUK 1947.3.4.95 (original number NHMUK 1876.6.21.39 [Syntype])

Notes: Catalogued in the Natural History Museum, London (NHMUK) collections as *Chelonoidis nigra abingdonii*. The collector of this specimen was Commander Cookson of the H.M.S. *Peterel*, who in an 1875 expedition to the Galápagos captured four tortoises from Pinta Island [26-28].

*Sample ID 45*

Taxon: *Chelonoidis niger abingdonii*

Galápagos Island: Pinta Island

Collection: Natural History Museum, London

Sex: Male

Age: Unknown

Date: 1875

Catalogue Number: NHMUK 1947.3.4.39 (original number NHMUK 1876.6.21.44 [Syntype])

Notes: Catalogued in the NHMUK collections as *Chelonoidis nigra abingdonii*. See Sample ID 44 note.

*Sample ID 46*

Taxon: *Chelonoidis niger chathamensis*

Galápagos Island: San Cristóbal Island

Collection: Natural History Museum, London

Sex: Male

Age: Unknown

Date: 1884

Catalogue Number: NHMUK 1949.1.4.44

Notes: Catalogued in the NHMUK collections as *Chelonoidis nigra chathamensis*. NHMUK and historic records indicate that this specimen derives from San Cristóbal Island and was collected by an Italian vessel. Lord Walter Rothschild [29] writes “the male, a stuffed specimen, was received in March 1884 on Chatham Island, during the voyage of the Italian vessel *Vetta Pisani*, and bears the Florence Museum Register No. M2454, Coll. 25. 1896.”

*Sample ID 47*

Taxon: *Chelonoidis niger duncanensis*

Galápagos Island: Pinzón Island

Collection: Natural History Museum, London

Sex: Unknown

Age: Unknown

Date: 1891-1906

Catalogue Number: NHMUK 1974.2454

Notes: Catalogued in the NHMUK collections as *Chelonoidis nigra ephippium*. The Pinzón Island tortoises in the NHMUK were obtained by Lord Rothschild [29] through a variety of expeditions or collections in the Galápagos, including by Baur (ca. 1891), Webster-Harris (ca. 1897), Heller and Snodgrass (ca. 1898-1899), Johnson-Green (ca. 1900-1901), Noyes (ca. 1900) and R.H. Beck (ca. 1901-1902 and 1905-1906). Since surviving NHMUK records do not specify from which expedition this tortoise derives, it has a *terminus post quem* death date of 1891 and *terminus ante quem* death date of 1906.

*Sample ID 48*

Taxon: *Chelonoidis niger duncanensis*

Galápagos Island: Pinzón Island

Collection: Natural History Museum, London

Sex: Unknown

Age: Unknown

Date: 1891-1906

Catalogue Number: NHMUK 1900.3.27.1

Notes: Catalogued in the NHMUK collections as *Chelonoidis nigra ephippium*. See Sample ID 47 note.

*Sample ID 49*

Taxon: *Chelonoidis niger duncanensis*

Galápagos Island: Pinzón Island

Collection: Natural History Museum, London

Sex: Male

Age: Unknown

Date: 1897

Catalogue Number: NHMUK 1949.1.3.72

Notes: Catalogued in the NHMUK collections as *Chelonoidis nigra ephippium*. A museum record associated with this tortoise reads, “Harris, Number 24” which matches Rothchild’s [29] table of *C. n. duncanensis* specimens. Given that Harris collected tortoises for Rothschild in 1897, this specimen dates to this year. A NHMUK catalogue note for this specimen indicates that it died in San Francisco, prior to being transported to Rothschild. Thus, we assume the stable isotope data for this specimen reflects dietary ecology in the Galápagos.

*Sample ID 50*

Taxon: *Chelonoidis niger duncanensis*

Galápagos Island: Pinzón Island

Collection: Natural History Museum, London

Sex: Female

Age: Unknown

Date: 1891-1906

Catalogue Number: NHMUK 1900.3.27.2

Notes: Catalogued in the NHMUK collections as *Chelonoidis nigra ephippium*. See Sample ID 47 note.

*Sample ID 51*

Taxon: *Chelonoidis niger duncanensis*

Galápagos Island: Pinzón Island

Collection: Natural History Museum, London

Sex: Unknown

Age: Unknown

Date: 1891-1906

Catalogue Number: NHMUK 1974.4.2453

Notes: Catalogued in the NHMUK collections as *Chelonoidis nigra ephippium*. See Sample ID 47 note.

*Sample ID 52*

Taxon: *Chelonoidis niger niger*

Galápagos Island: Floreana Island

Collection: Natural History Museum, London

Sex: Male

Age: Unknown

Date: 1833

Catalogue Number: NHMUK 1949.1.3.90

Notes: Catalogued in the NHMUK collections as *Chelonoidis nigra nigra*. Rothschild [29] writes, “The two specimens in the Tring Museum were formerly the property of the Peabody Museum, Salem, Mass., and were lent to me for study.” The Natural History Museum, London, register for this specimen supports that this tortoise originated from the Peabody Museum stating, “exch. Mus. Peabody Acad. [sic]” According to Günther [30], who also wrote about these Peabody tortoises, “The first is one of two specimens in the museum of the Peabody Academy of Science in Salem, Massachusetts. They are said to have been brought to the United States *at the same time when the two living ones* were given to the Boston Natural History Society, and that all of them came from Charles Island. As both of these Salem specimens were found to be ‘absolutely identical,’ one of them was ceded to the Rothschild Museum” (emphasis added). This is a significant record as the Boston Natural History Society [31; see also 32] indicates that in June 1834 the society received the following donations: “Two Small Galipagos tortoises, Lieutenant Babbit, (U.S. Navy), Two gigantic Galipagos Tortoises (living) weighing near three hundred and twenty pounds each. Capt. John Downes, (U.S. Navy) [sic].” Therefore, this specimen is one of the two non-living Babbit tortoises captured and killed in 1833, donated to the Boston Natural History Society in 1834 and then loaned to Rothschild in the early 1900s. This specimen is labeled at the Co-Type in the NHMUK catalogue. Preliminary DNA from this specimen confirms the species identification as *C. nigra* (Personal communication with Evelyn Jensen, Adalgisa Caccone and Nicole Fusco, 2022).

*Sample ID 53*

Taxon: *Chelonoidis niger duncanensis*

Galápagos Island: Pinzón Island

Collection: Natural History Museum, London

Sex: Unknown

Age: Unknown

Date: 1901-1906

Catalogue Number: NHMUK 1974.2468

Notes: Catalogued in the NHMUK collections as *Testudo (Elephantopus) nigrita.* Historically, this suggests that the tortoise is from Santa Cruz Island (i.e., *C. n. porteri*) based on revised taxonomic nomenclature [1], however, NHMUK records indicate that this tortoise was collected by Beck from “Duncan Island”. See Sample ID 47 note – Beck collected between 1901-1906. Given the revision in taxonomic nomenclature for tortoises since the early 19^th^ century, and since Pinzón (Duncan) Island tortoises did not go extinct during this time (e.g., facilitating the need to bring Santa Cruz tortoises to Pinzón), we accept the taxonomy for this specimen as *C. n. duncanensis* from Pinzón Island collected sometime between 1901-1906.

*Sample ID 54*

Taxon: *Chelonoidis niger porteri*

Galápagos Island: Santa Cruz Island

Collection: Natural History Museum, London

Sex: Female

Age: Juvenile

Date: 1902

Catalogue Number: NHMUK 1949.1.3.94 (Type)

Notes: Catalogued in the NHMUK collections as *Chelonoidis nigra porteri*. Museum records indicate that this specimen was collected from “Indefatigable Island” and came from the London Zoo where it died in 1902. However, Rothschild [33] notes that Beck collected five dead tortoises from Santa Cruz Island in 1902. It is unclear if Beck’s tortoises were all sent to the NHMUK. Therefore, this tortoise likely died in 1902 but the stable isotope data associated with this tortoise must be evaluated with caution as it likely is not representative of the tortoise’s diet in the Galápagos and instead reflects a captive-fed diet.

*Sample ID 55*

Taxon: *Chelonoidis niger porteri*

Galápagos Island: Santa Cruz Island

Collection: Natural History Museum, London

Sex: Unknown

Age: Juvenile

Date: 1902?

Catalogue Number: NHMUK 1870.12.18.1

Notes: Catalogued in the NHMUK collections as *Chelonoidis nigra porteri*. See Sample ID 54 note. This tortoise also was reportedly from the London Zoo and has a catalogue note indicating it is from, “Chile, South America”.

*Sample ID 56*

Taxon: *Chelonoidis niger porteri*

Galápagos Island: Santa Cruz Island

Collection: Natural History Museum, London

Sex: Unknown

Age: Juvenile

Date: 1833

Catalogue Number: NHMUK 1876.10.23.2

Notes: Catalogued in the NHMUK collections as *Chelonoidis nigra porteri*. See Sample ID 54 note. NHMUK catalogue records indicate that this tortoise was registered in 1876, which means it was collected prior to this date. There are very few records of *C. n. porteri* collected and sent to England prior to the late-19^th^ and early-20^th^ century for Rothschild [29]. However, Rothschild does note that in the NHMUK collections there are *C. n. porteri* specimens that were collected in 1833, “from a whaling boat in Callao Bay by Mr. Wallace, of Distington, at the dispersal of whose museum I obtained them.” We argue that this indicates this Santa Cruz Island tortoise was collected in 1833.

*Sample ID 57*

Taxon: *Chelonoidis niger porteri*

Galápagos Island: Santa Cruz Island

Collection: Natural History Museum, London

Sex: Unknown

Age: Unknown

Date: 1891-1906

Catalogue Number: NHMUK 1974.2463

Notes: Catalogued in the NHMUK collections as *Chelonoidis nigra porteri*. Museum records indicate that this Santa Cruz Island tortoise was collected by either Baur or Beck. See Sample ID 47 note. Therefore, this tortoise has a *terminus post quem* death date of 1891 and a *terminus ante quem* death date of 1906.

**Radiocarbon Results Sheets (Figures)**


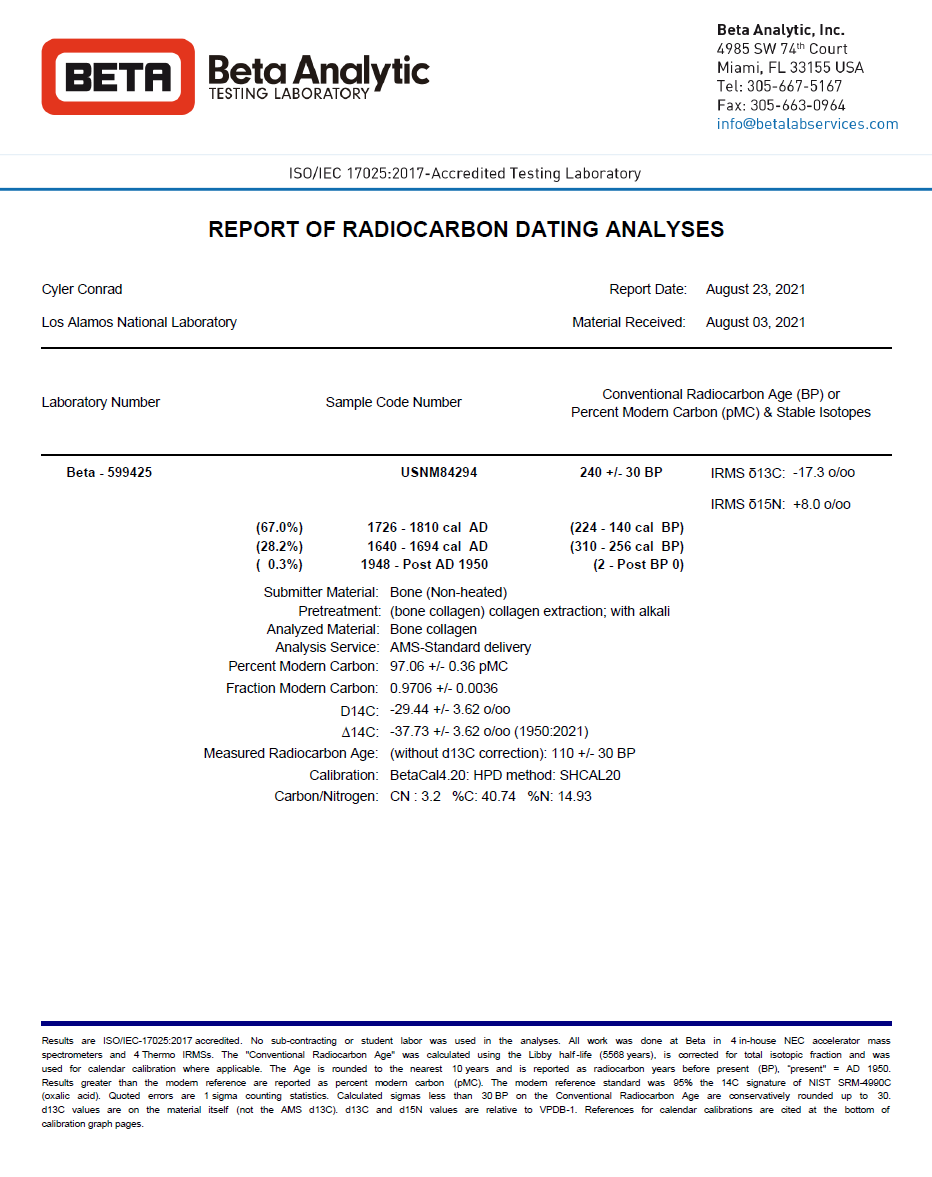


Fig. R1. Radiocarbon results sheet for sample USNM-84294.


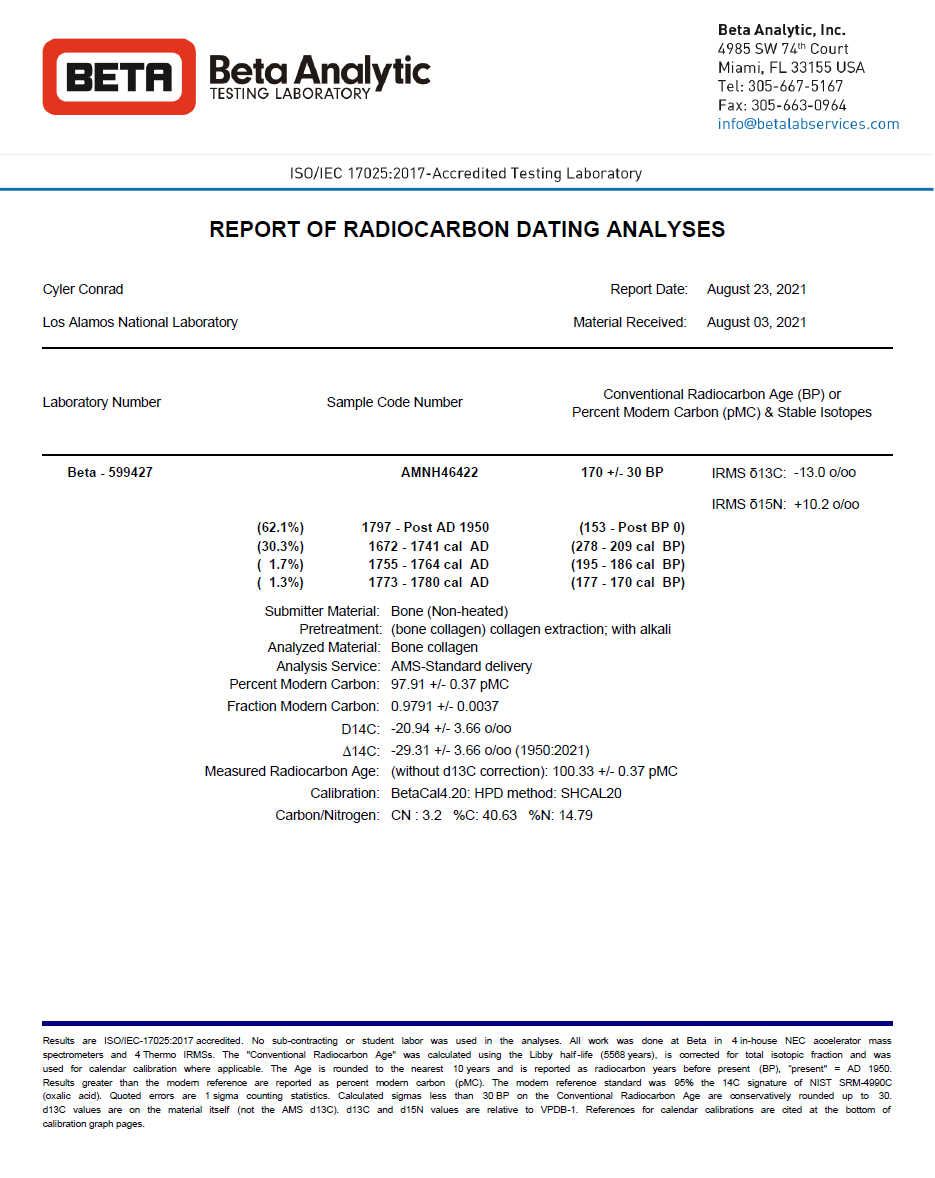


Fig. R2. Radiocarbon results sheet for sample AMNH-46422.


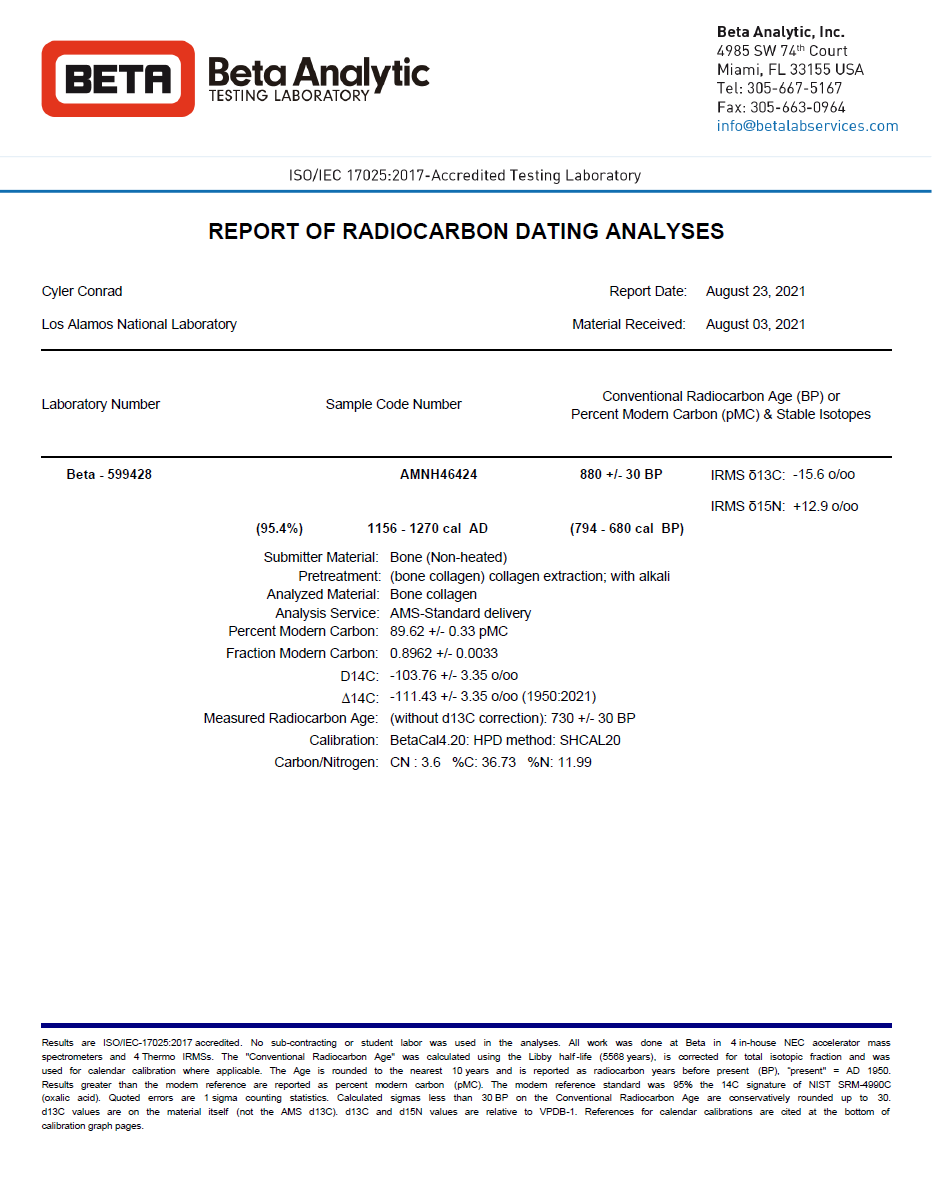


Fig. R3. Radiocarbon results sheet for sample AMNH-46424.

**Figures**

This supplemental text includes visualizations of a variety of Galápagos tortoise stable isotope data analyzed and discussed in text.


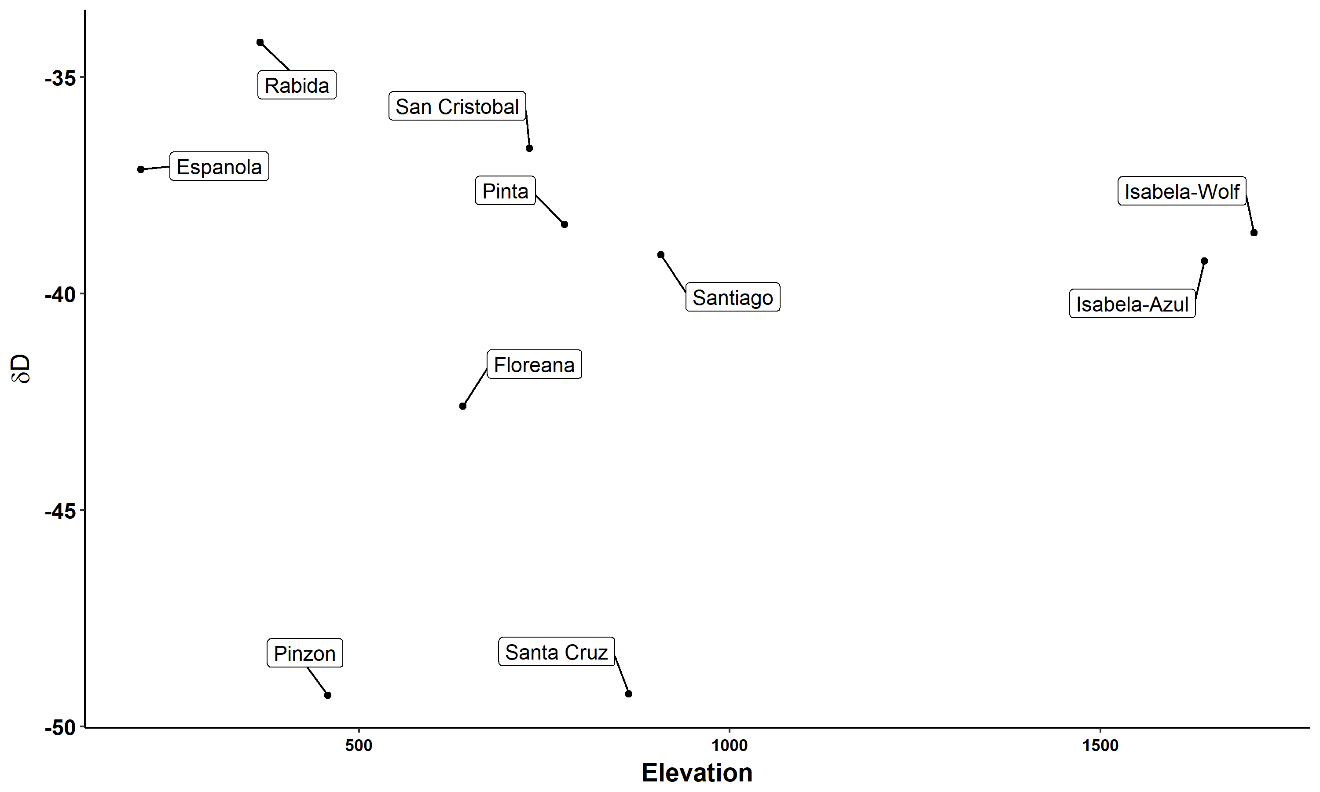


Fig. S1. Average Galápagos tortoise stable isotope ratios for δD per maximum island elevation.


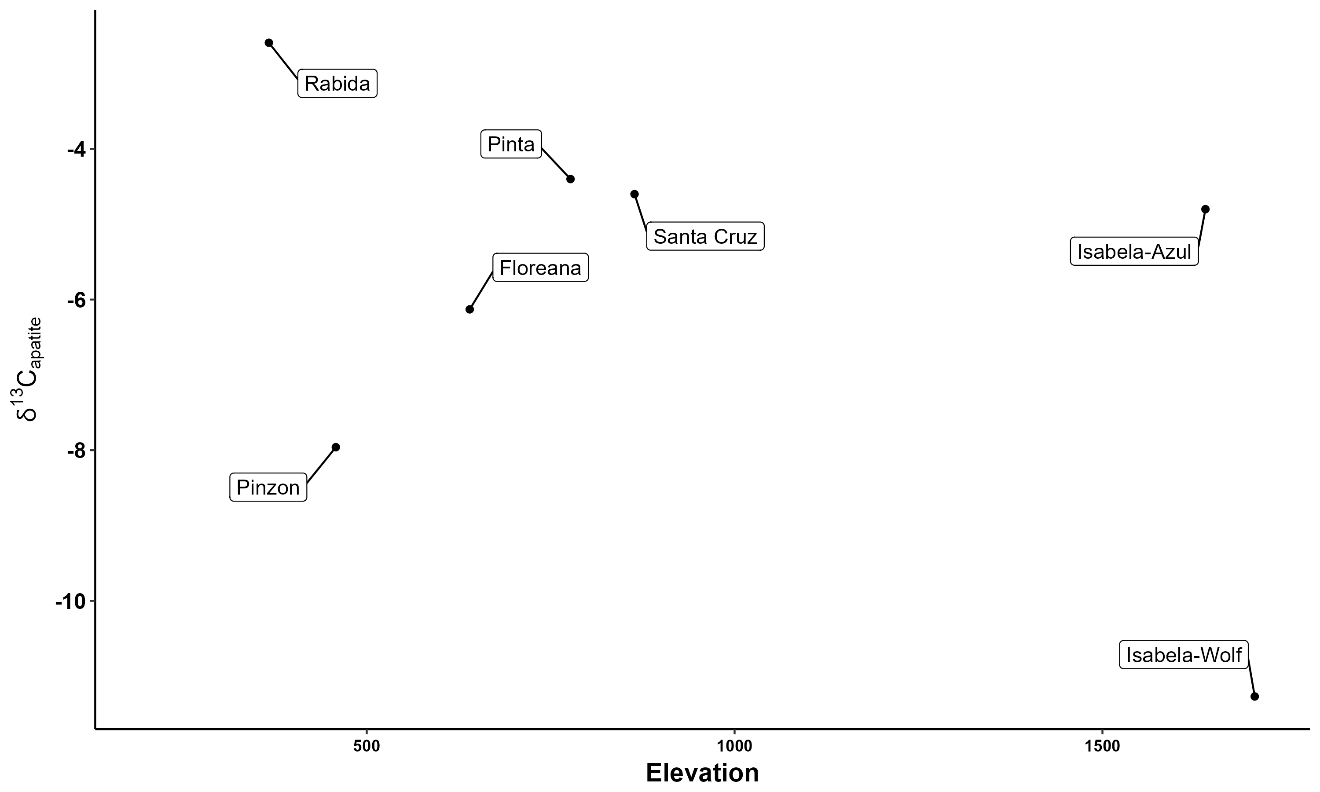


Fig. S2. Average Galápagos tortoise stable isotope ratios for δ^13^C_apatite_ per maximum island elevation.


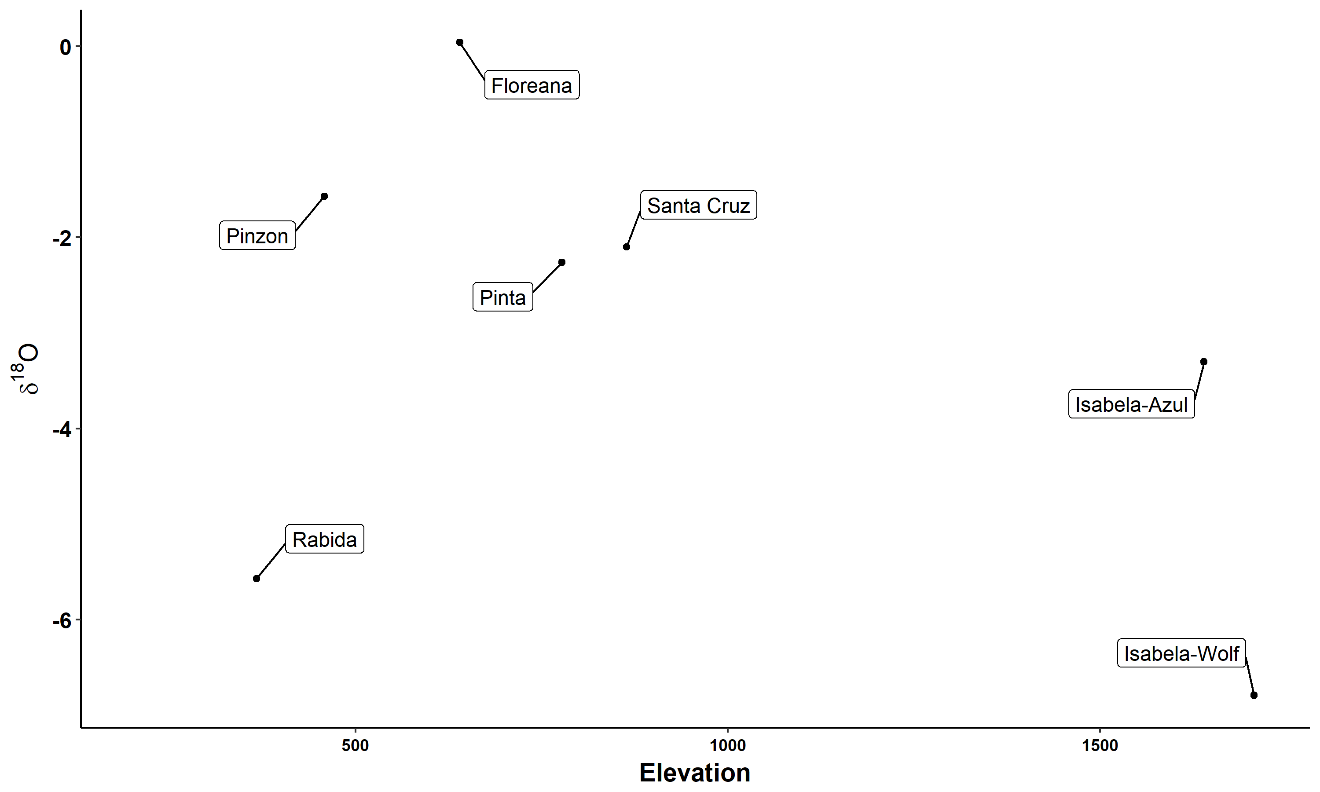


Fig. S3. Average Galápagos tortoise stable isotope ratios for δ^18^O_apatite_ per maximum island elevation.
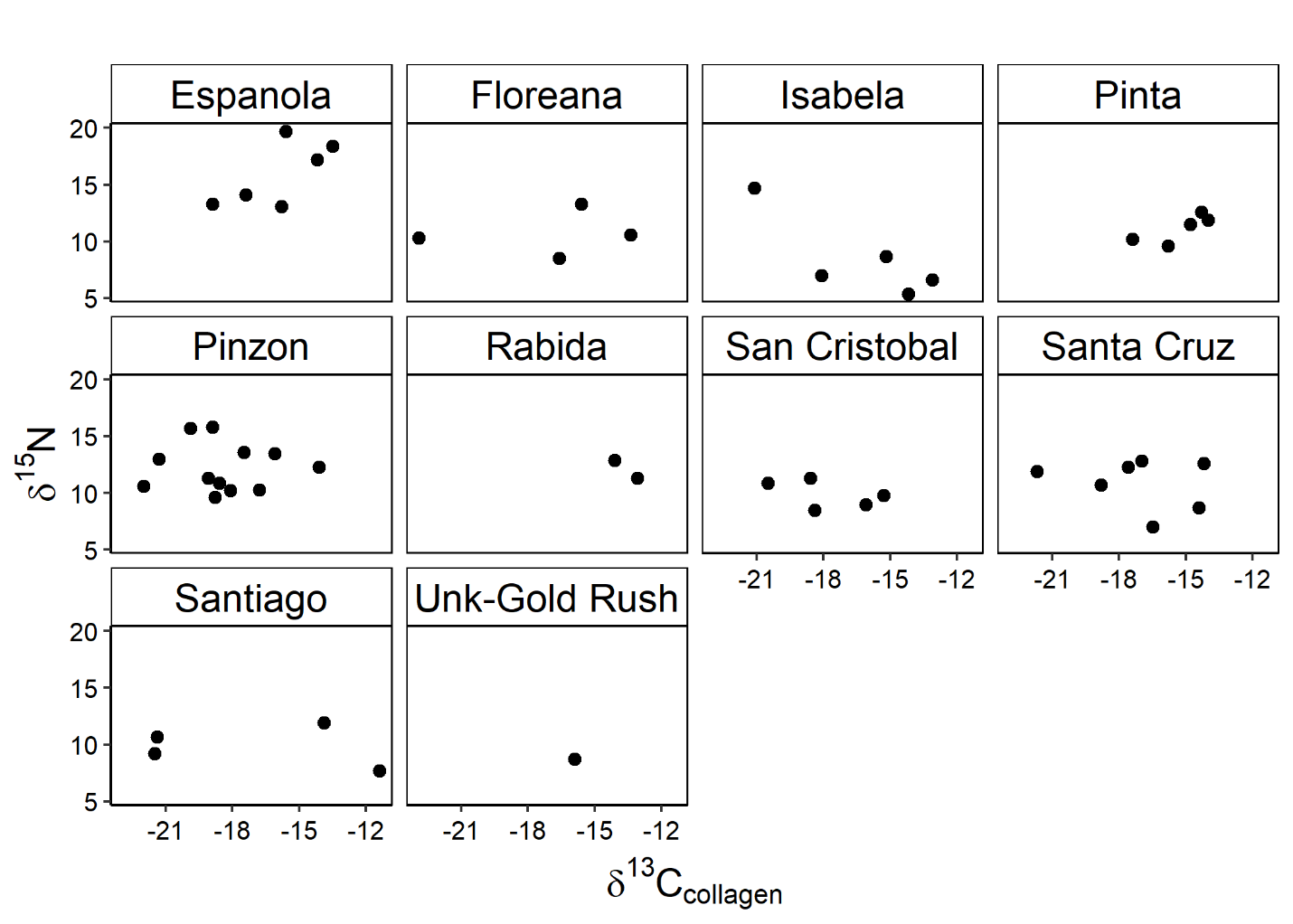


Fig. S4. Relationship between δ^13^C_collagen_ and δ^15^N_collagen_ for all Galápagos tortoises analyzed in this study, per island.


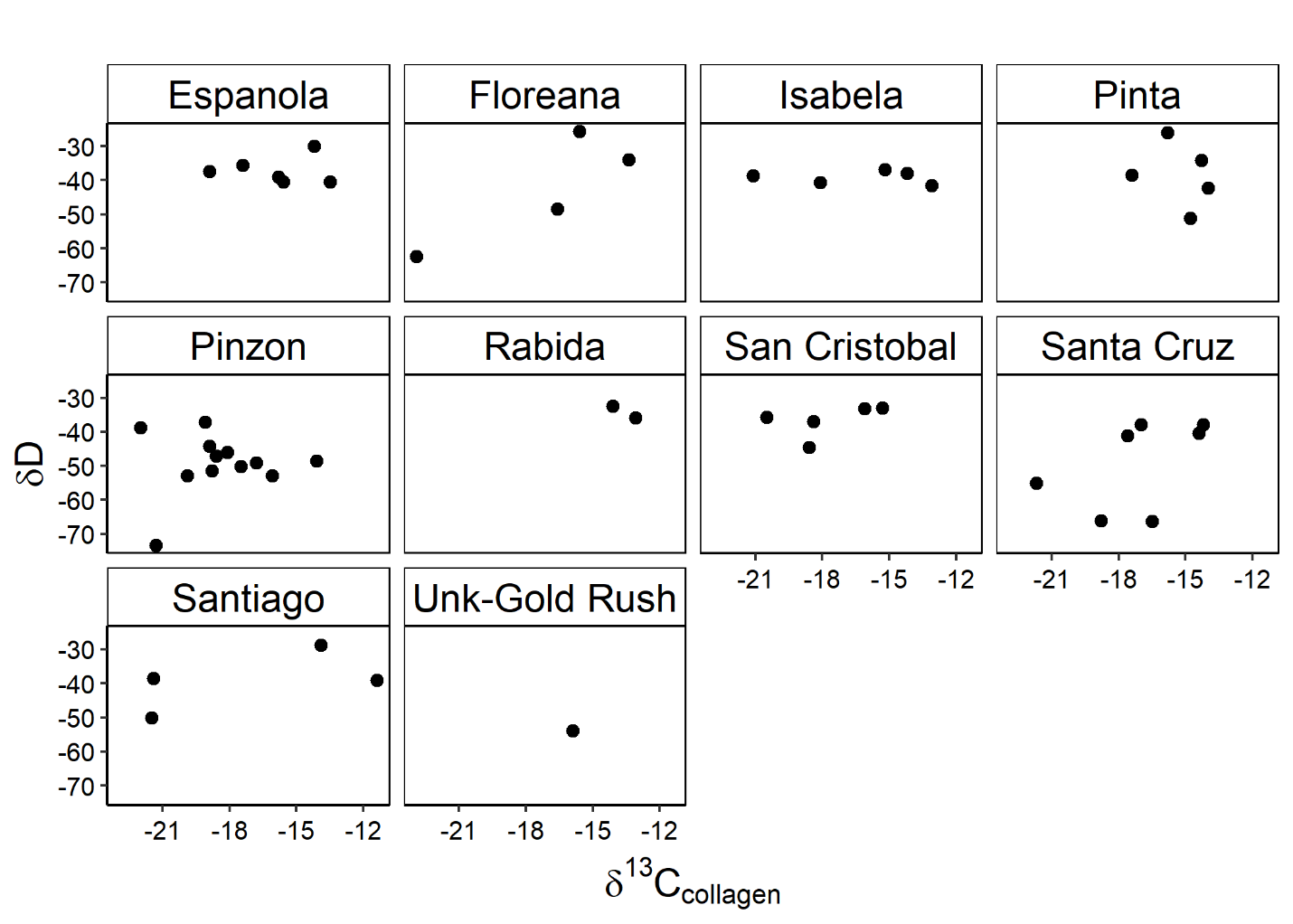


Fig. S5. Relationship between δ^13^C_collagen_ and δD for all Galápagos tortoises analyzed in this study, per island.


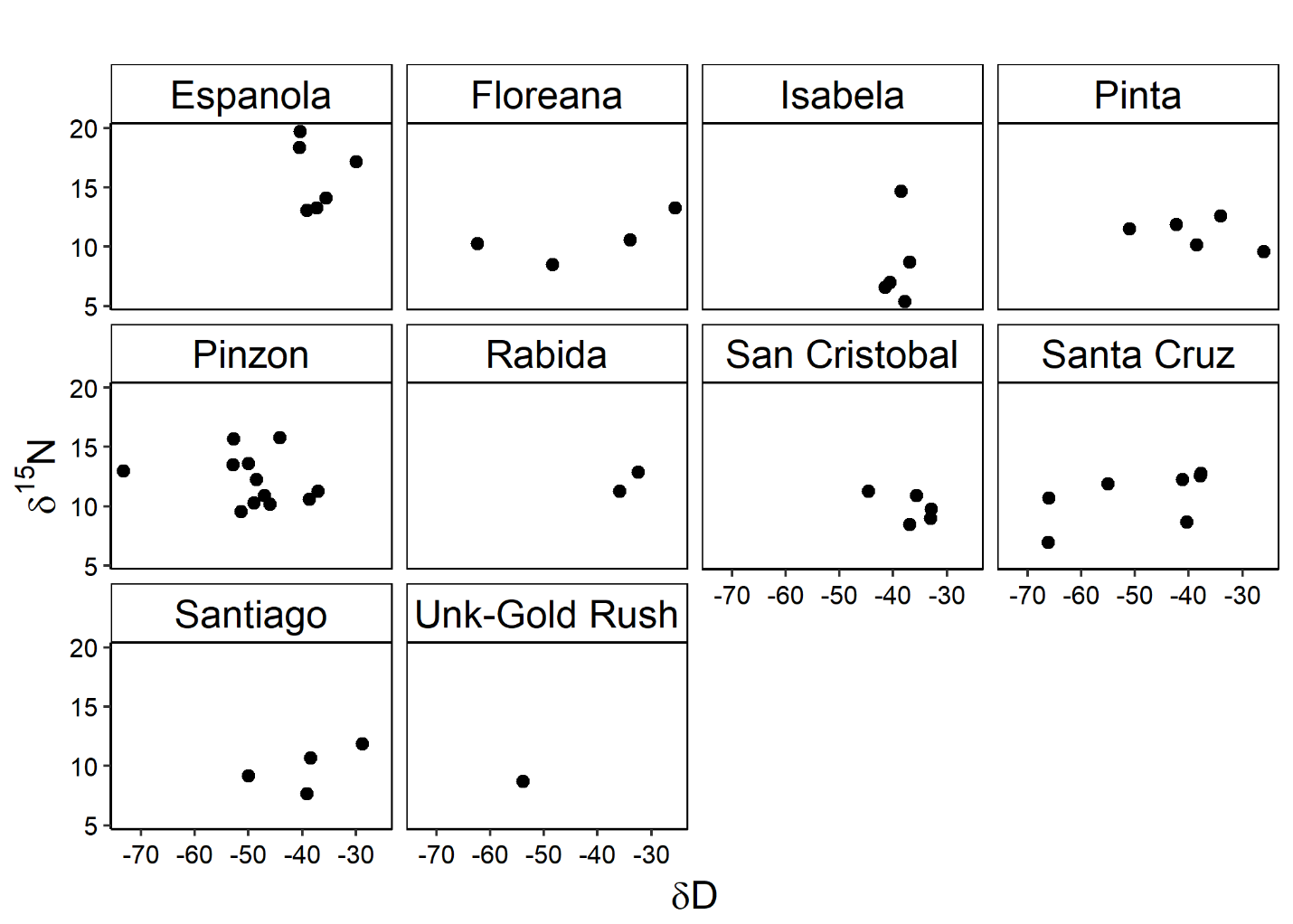


Fig. S6. Relationship between δD and δ^15^N_collagen_ for all Galápagos tortoises analyzed in this study, per island.


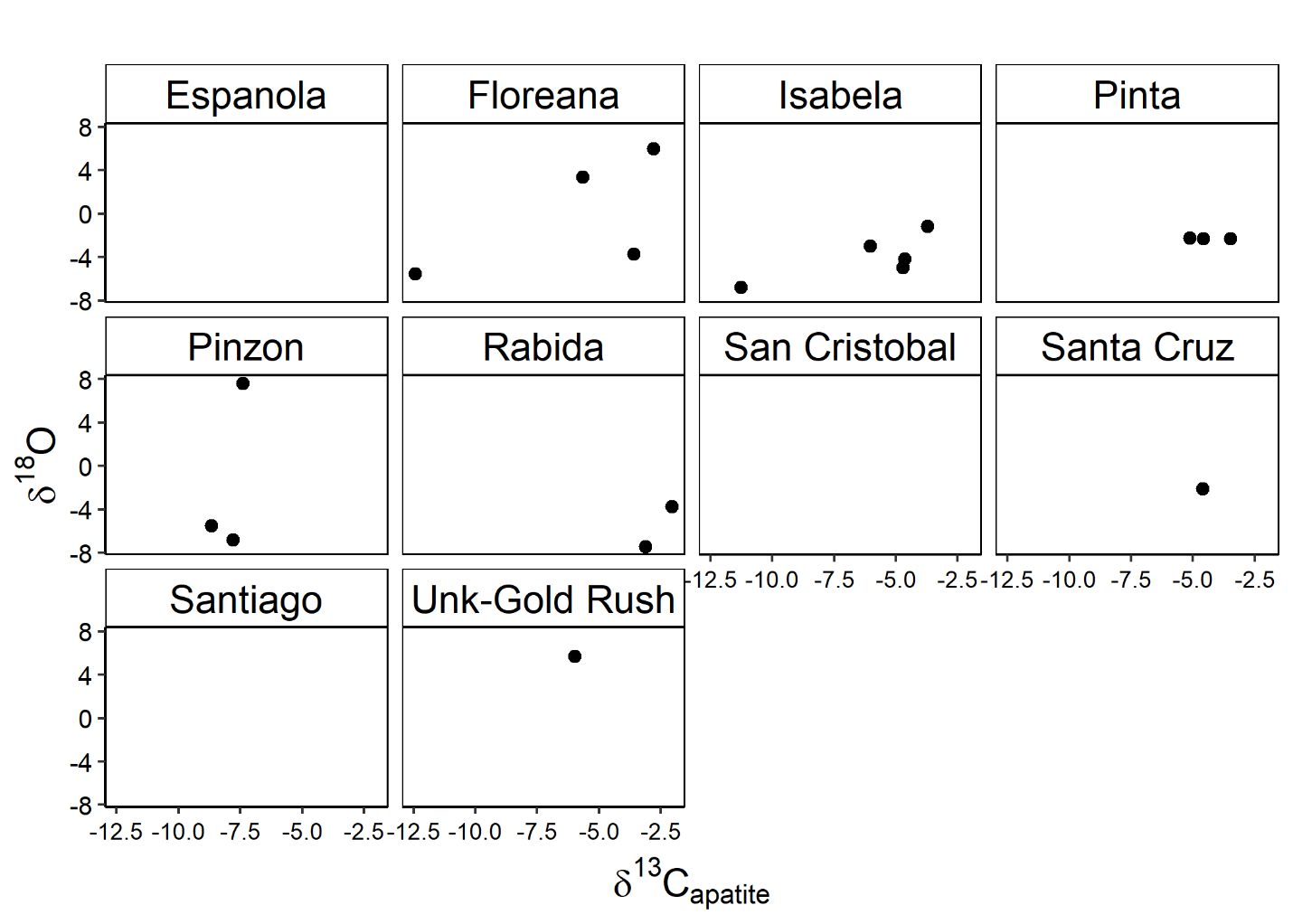


Fig. S7. Relationship between δ^13^C_apatite_ and δ^18^O_apatite_ for all Galápagos tortoises analyzed in this study, per island.


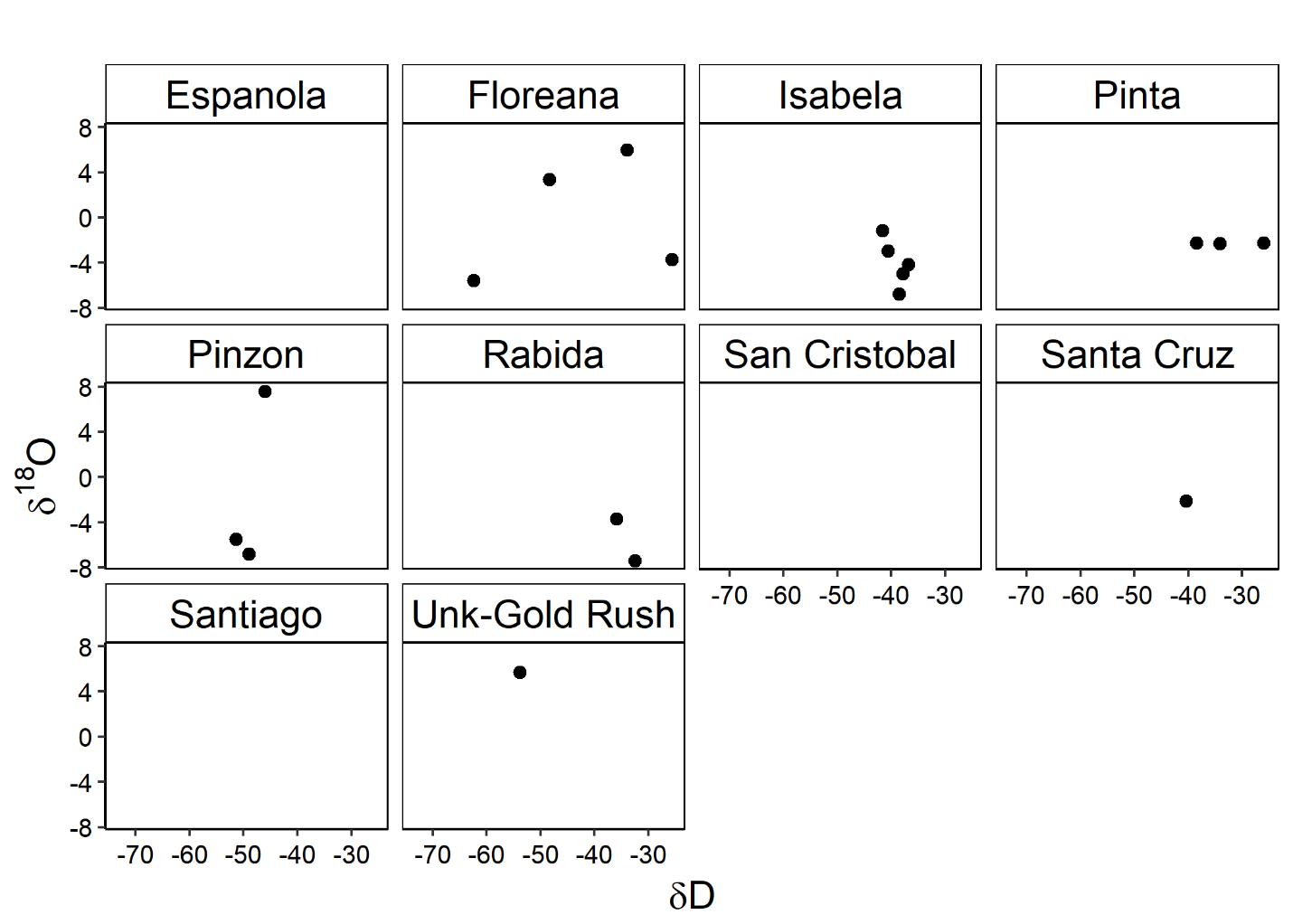


Fig. S8. Relationship between δD and δ^18^O_apatite_ for all Galápagos tortoises analyzed in this study, per island.


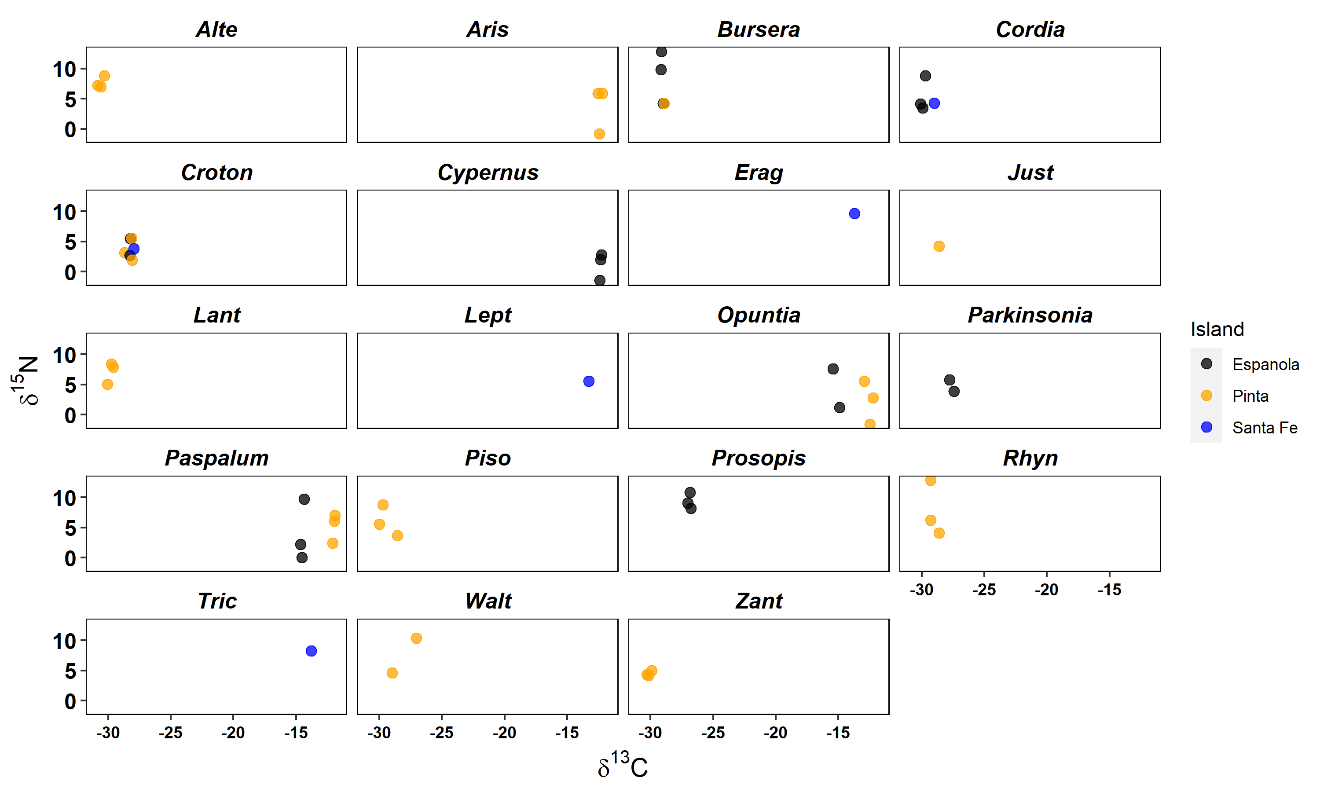


Fig. S9. Modern plant δ^13^C and δ^15^N ratios from the Galápagos. See Supplemental Table 2 for additional details.


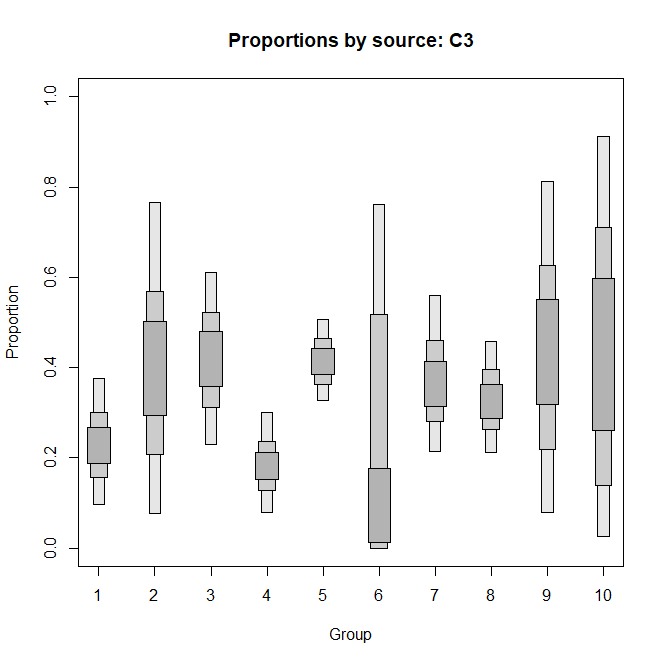
Fig. S10. The proportion of C_3_ plants per Galápagos tortoise taxa/island. See text Table 2 for group identifications.


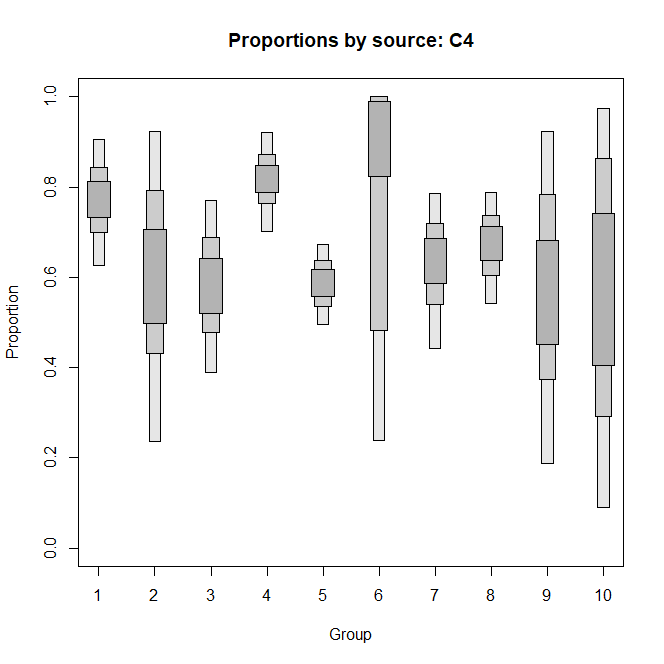
Fig. S11. The proportion of C_4_ plants per Galápagos tortoise taxa/island. See text Table 2 for group identifications.


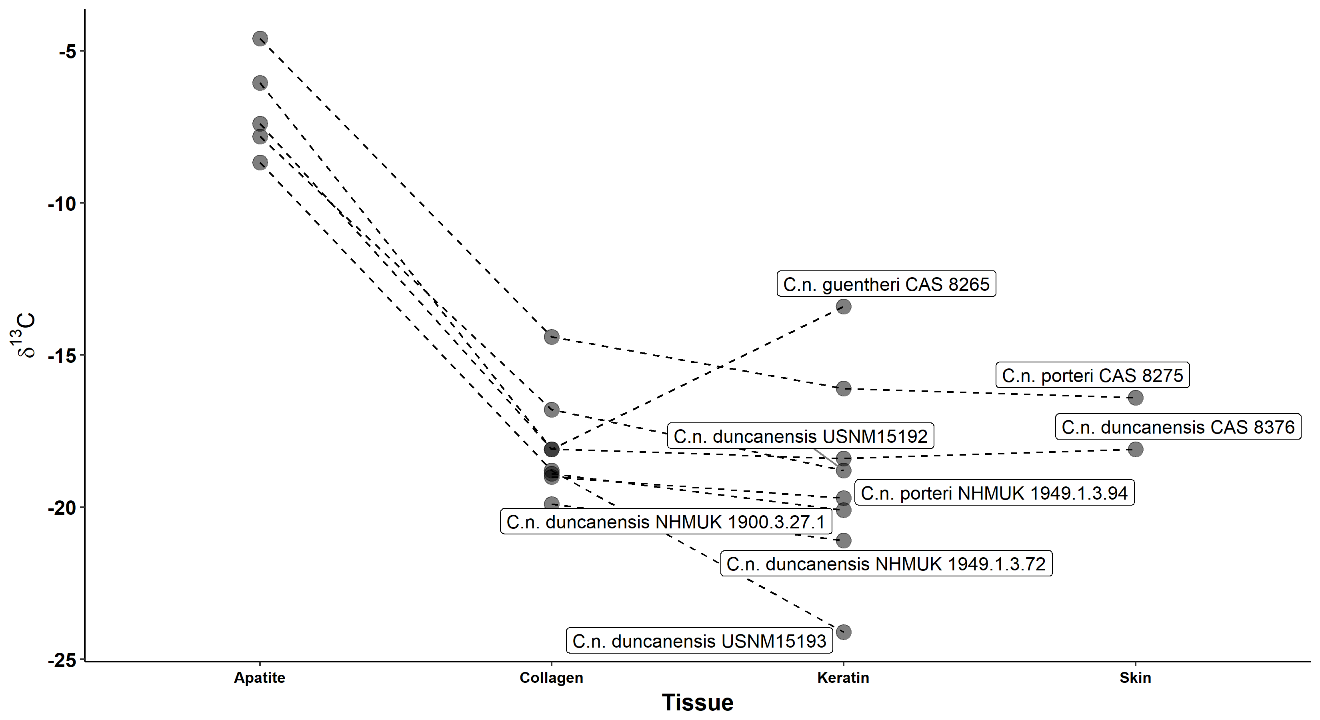


Fig. S12. Tissue specific discrimination in δ^13^C. Labels indicate specimen numbers.


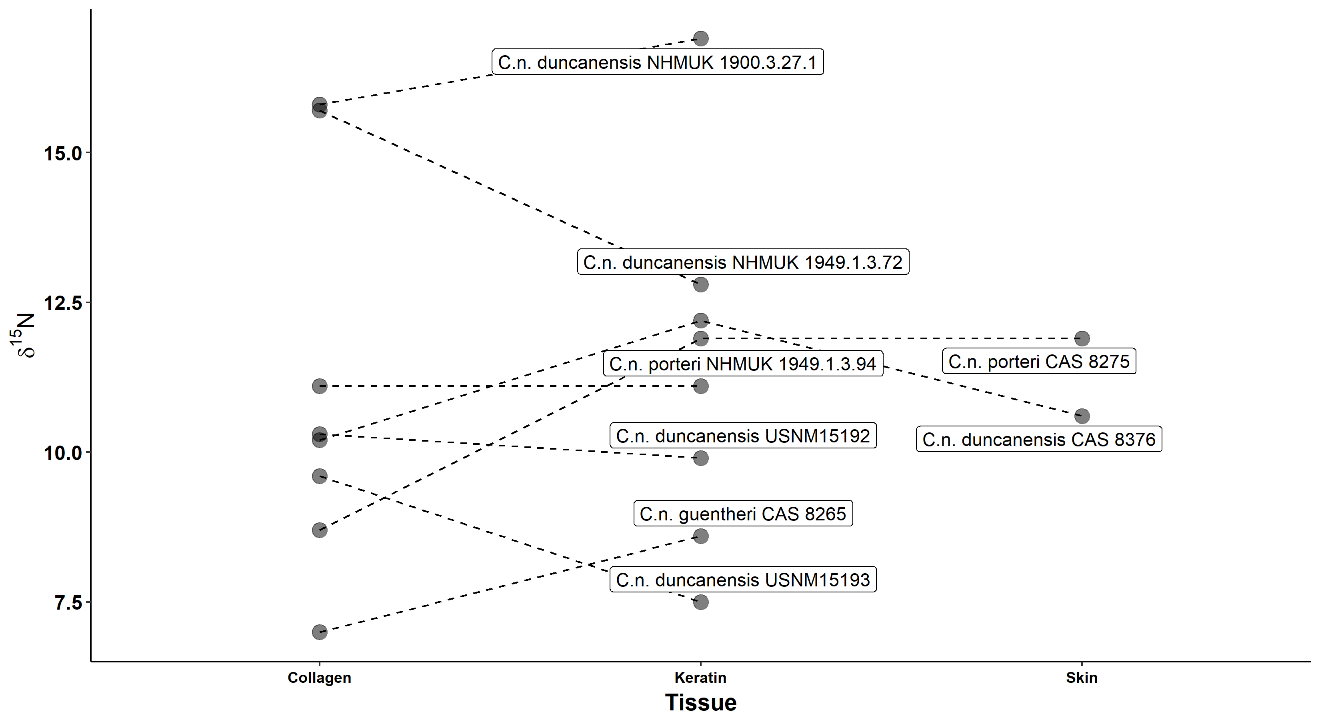


Fig. S13. Tissue specific discrimination in δ^15^N. Labels indicate specimen numbers.


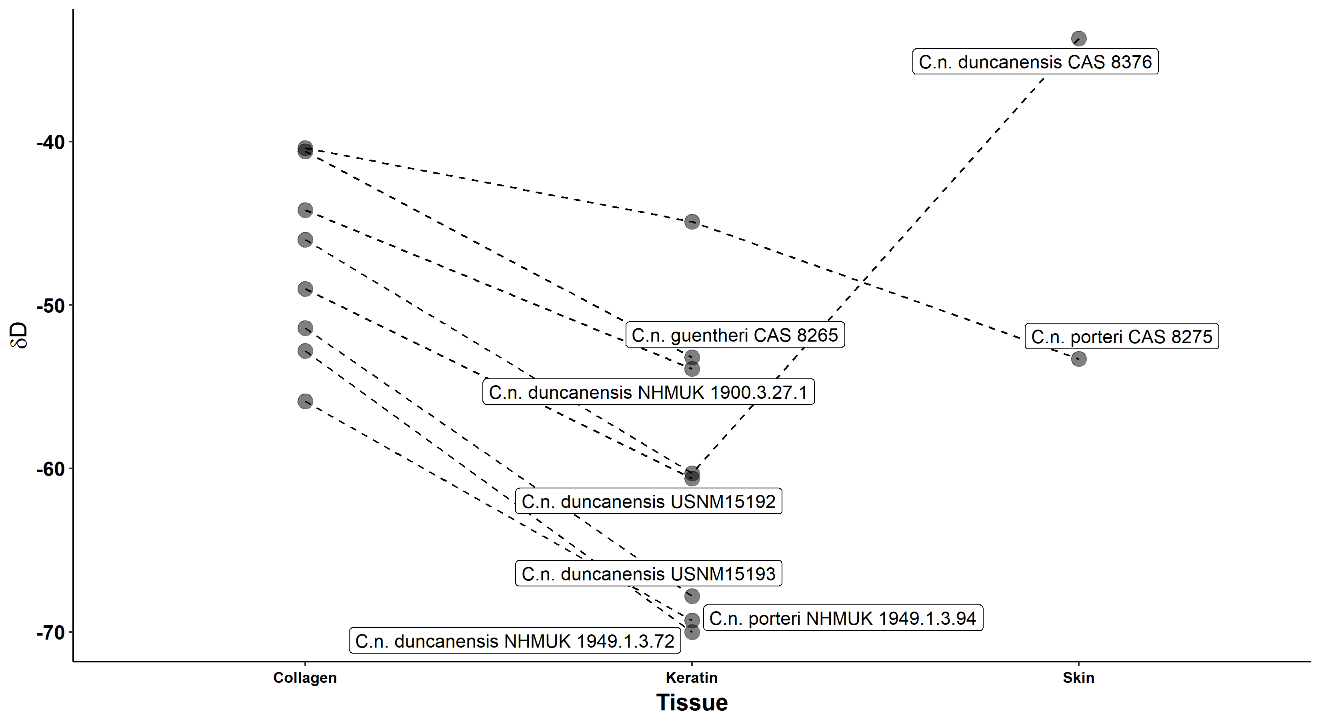


Fig. S14. Tissue specific discrimination in δD. Labels indicate specimen numbers.


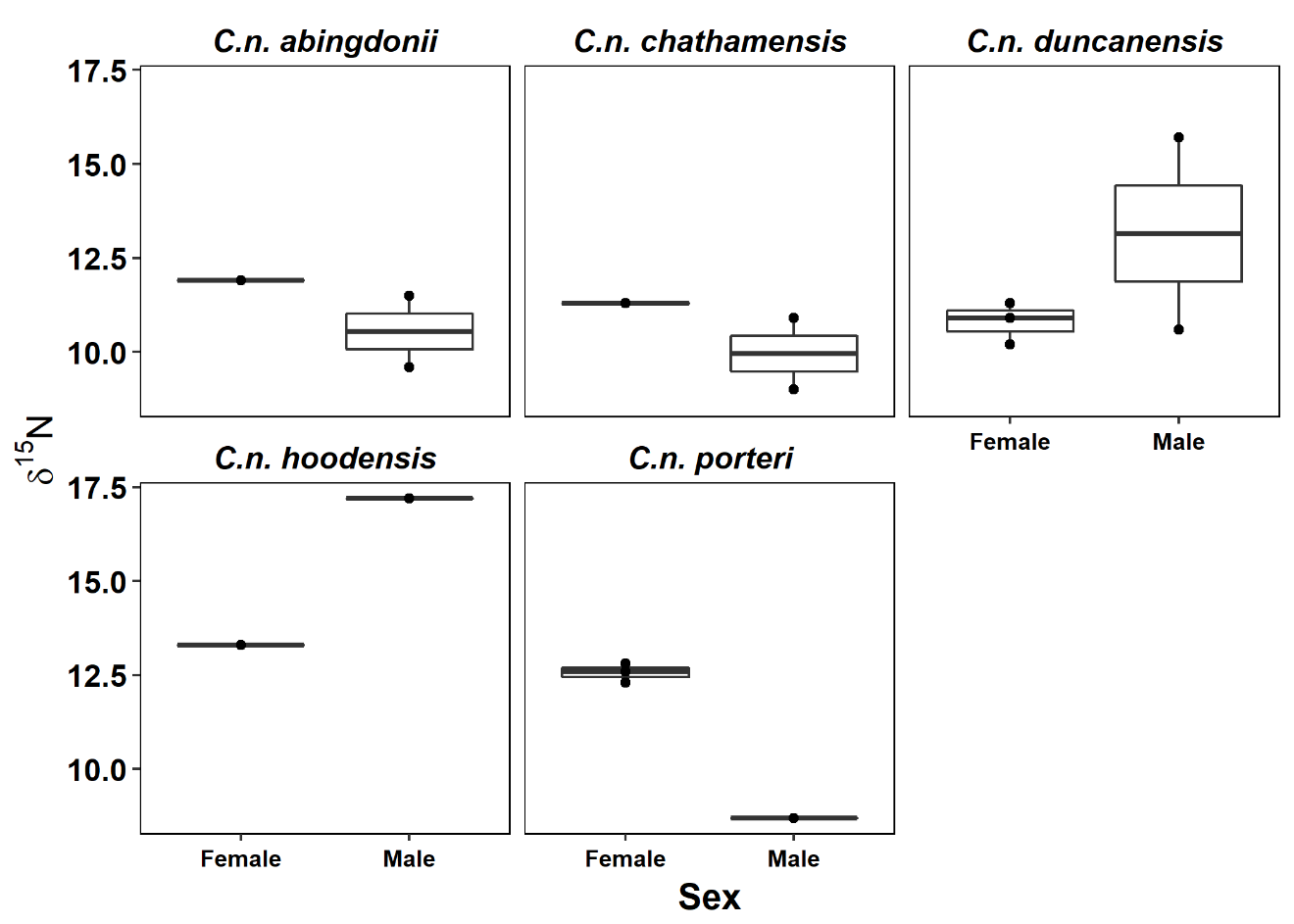


Fig. S15. Sex based differences in δ^15^N for Galápagos tortoises on Pinta (*C. n. abingdonii*), San Cristóbal (*C. n. chathamensis*), Pinzón (*C. n. duncanensis*), Española (*C. n. hoodensis*) and Santa Cruz (*C. n. porteri*).


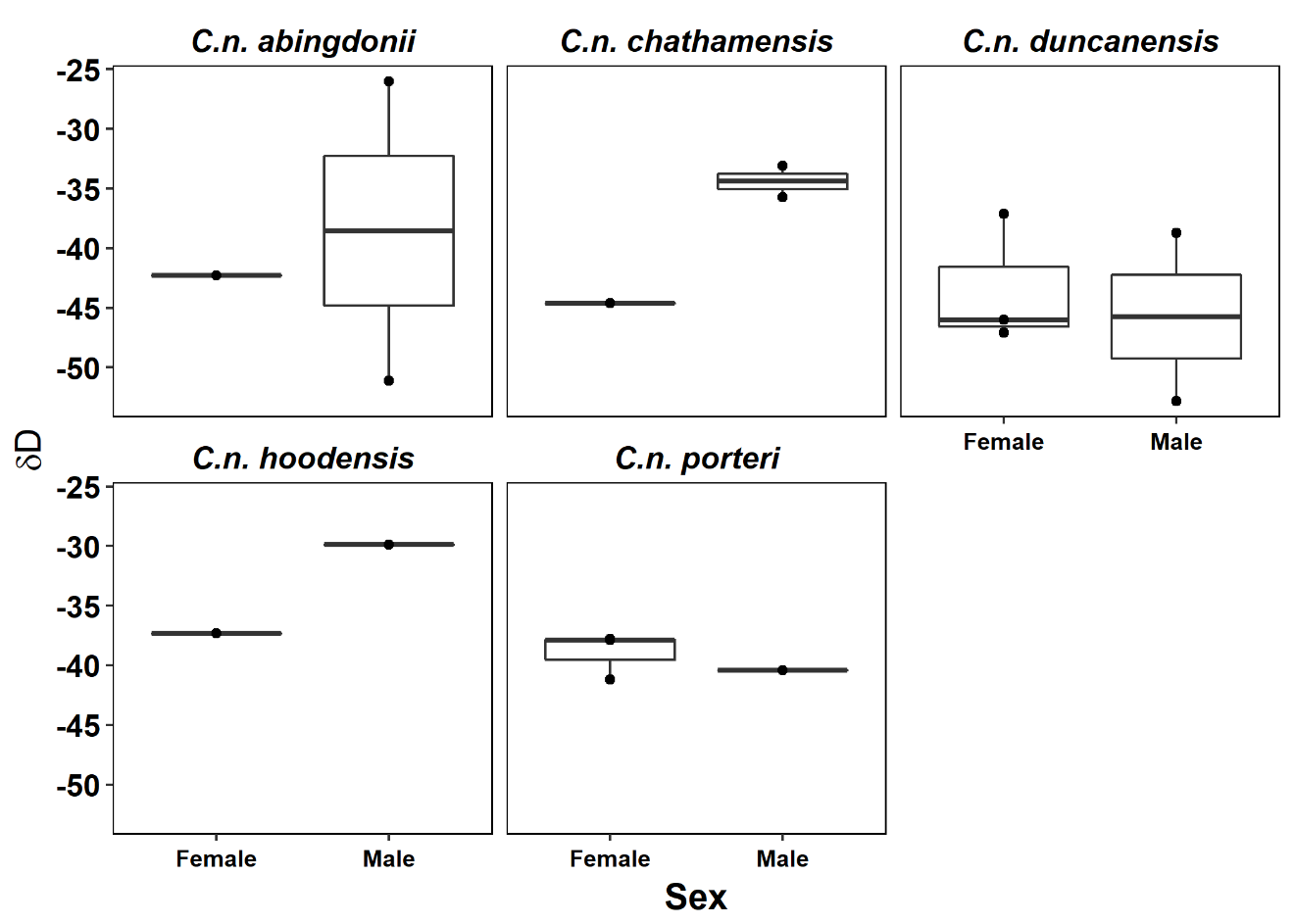


Fig. S16. Sex based differences in δD for Galápagos tortoises on Pinta (*C. n. abingdonii*), San Cristóbal (*C. n. chathamensis*), Pinzón (*C. n. duncanensis*), Española (*C. n. hoodensis*) and Santa Cruz (*C. n. porteri*).


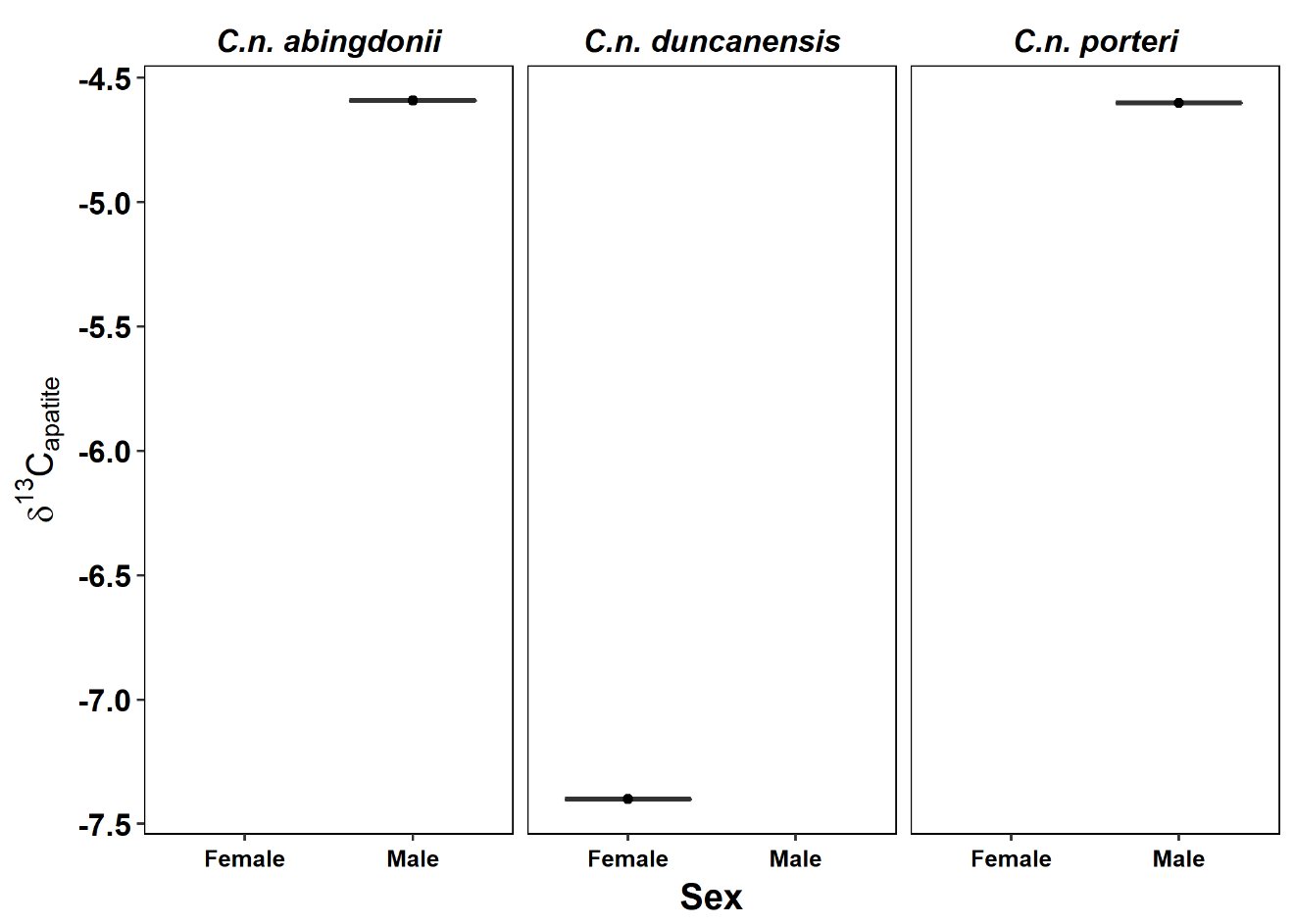


Fig. S17. Sex based differences in δ^13^C_apatite_ for Galápagos tortoises on Pinta (*C. n. abingdonii*), Pinzón (*C. n. duncanensis*), and Santa Cruz (*C. n. porteri*).


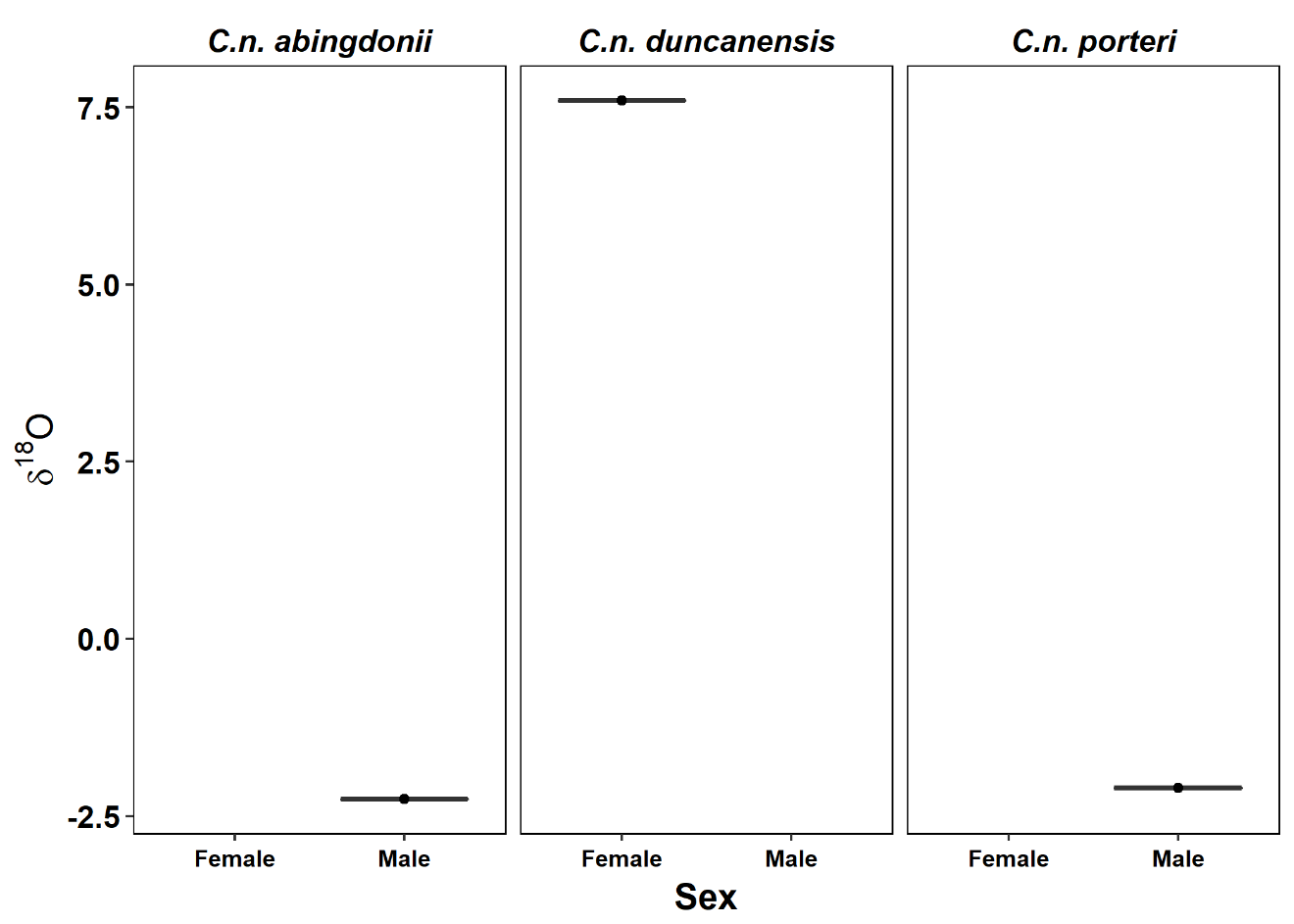


Fig. S18. Sex based differences in δ^18^O_apatite_ for Galápagos tortoises on Pinta (*C. n. abingdonii*), Pinzón (*C. n. duncanensis*), and Santa Cruz (*C. n. porteri*).


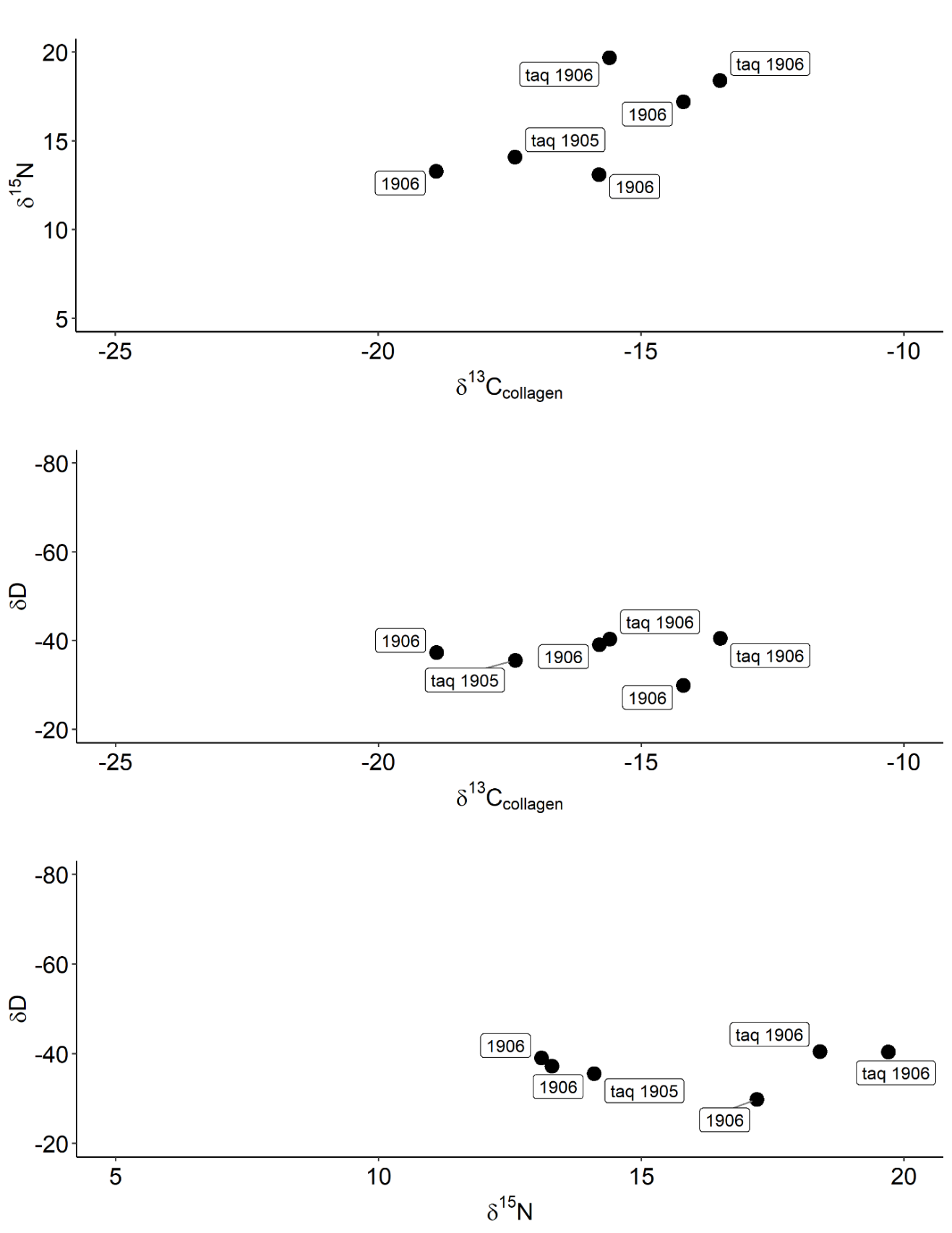


Fig. S19. Diachronic trends in *C. n. hoodensis* from Española Island.


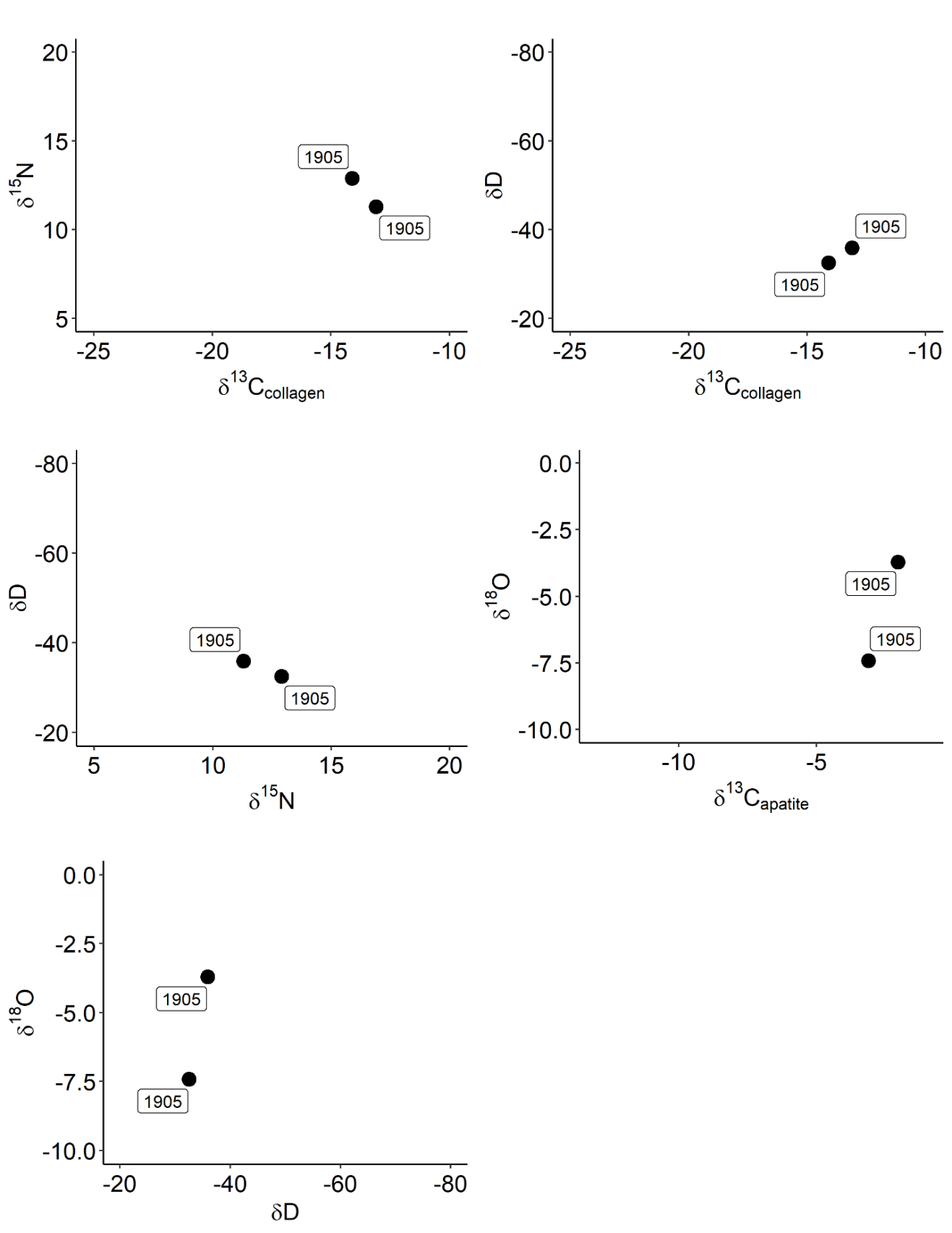


Fig. S20. Diachronic trends in *C. n. vicina* from Rabida Island.


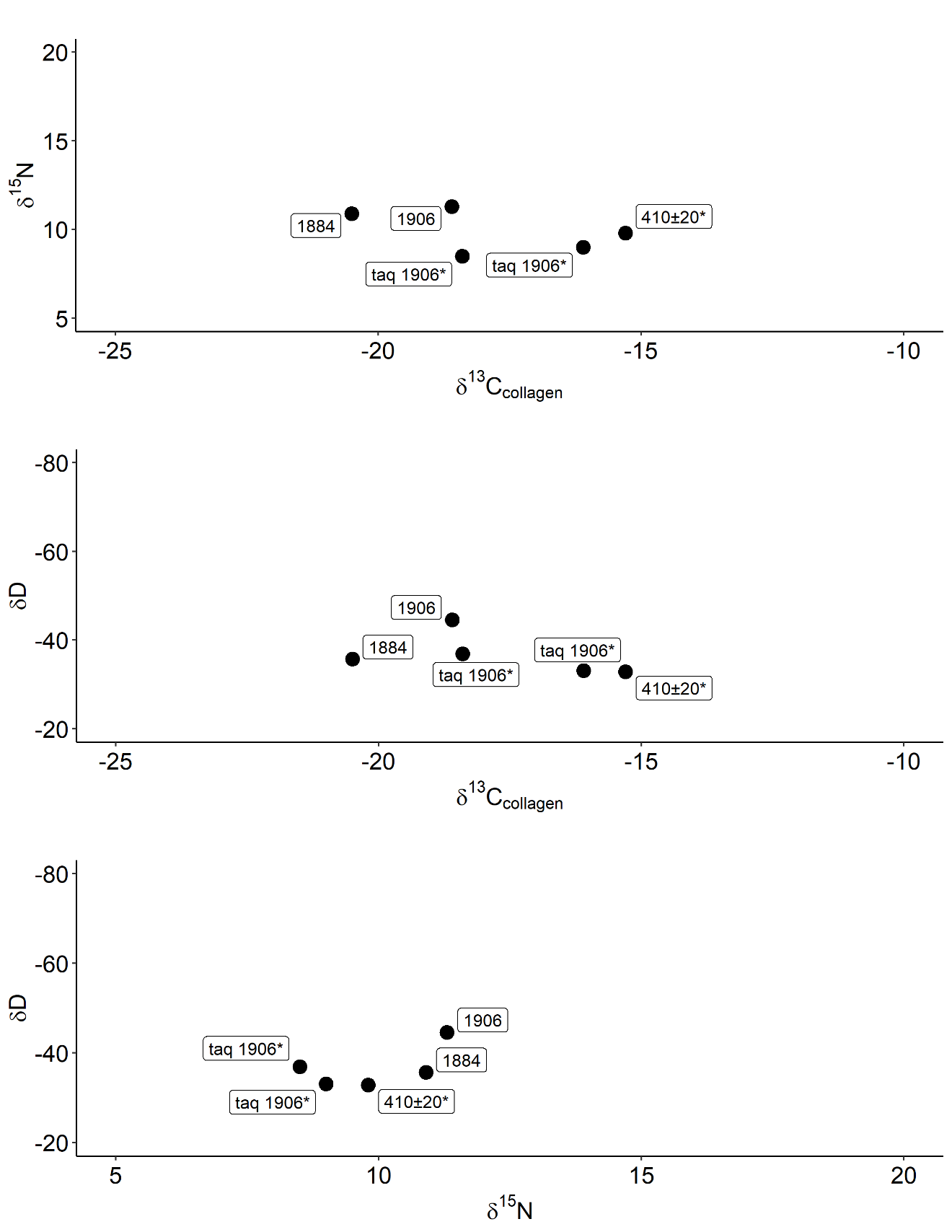


Fig. S21. Diachronic trends in *C. n. chathamensis* from San Cristóbal Island. The tortoises marked with “*” are from the now extinct clade identified by Jensen and colleagues (7). One of those tortoises (CAS-8129) returned a radiocarbon age of 410±20 years before present.


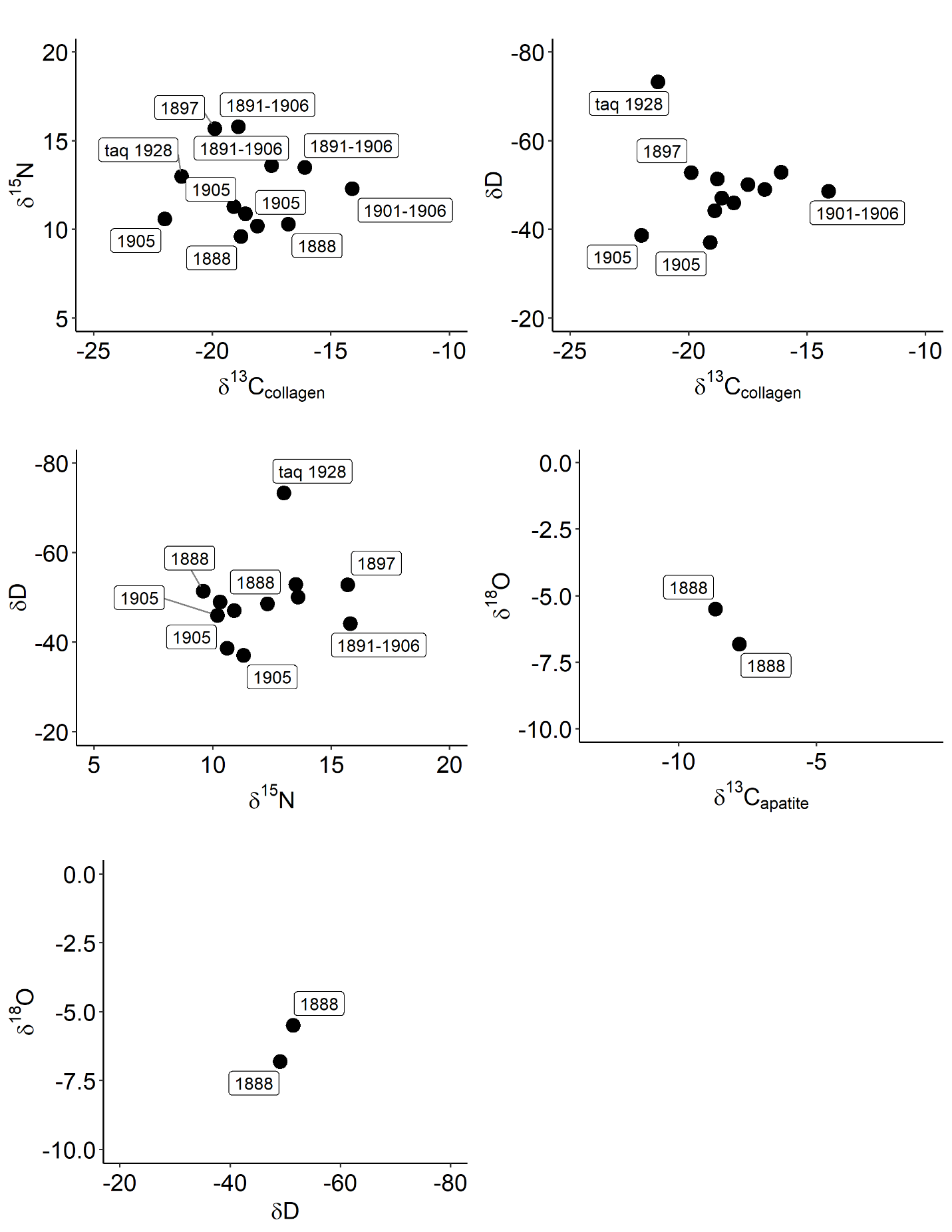


Fig. S22. Diachronic trends in *C. n. duncanensis* from Pinzón Island.


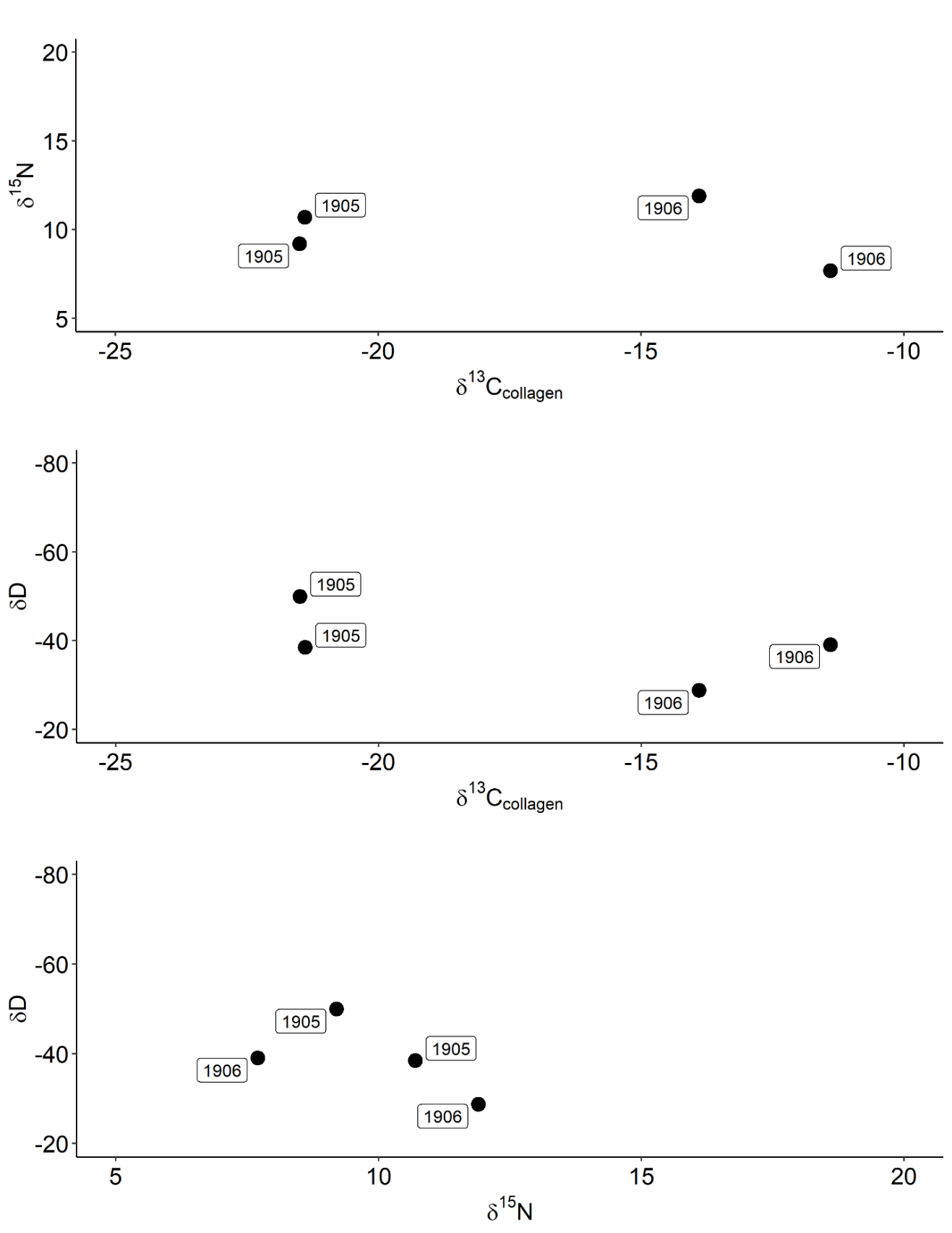


Fig. S23. Diachronic trends in *C. n. darwini* from Santiago Island.


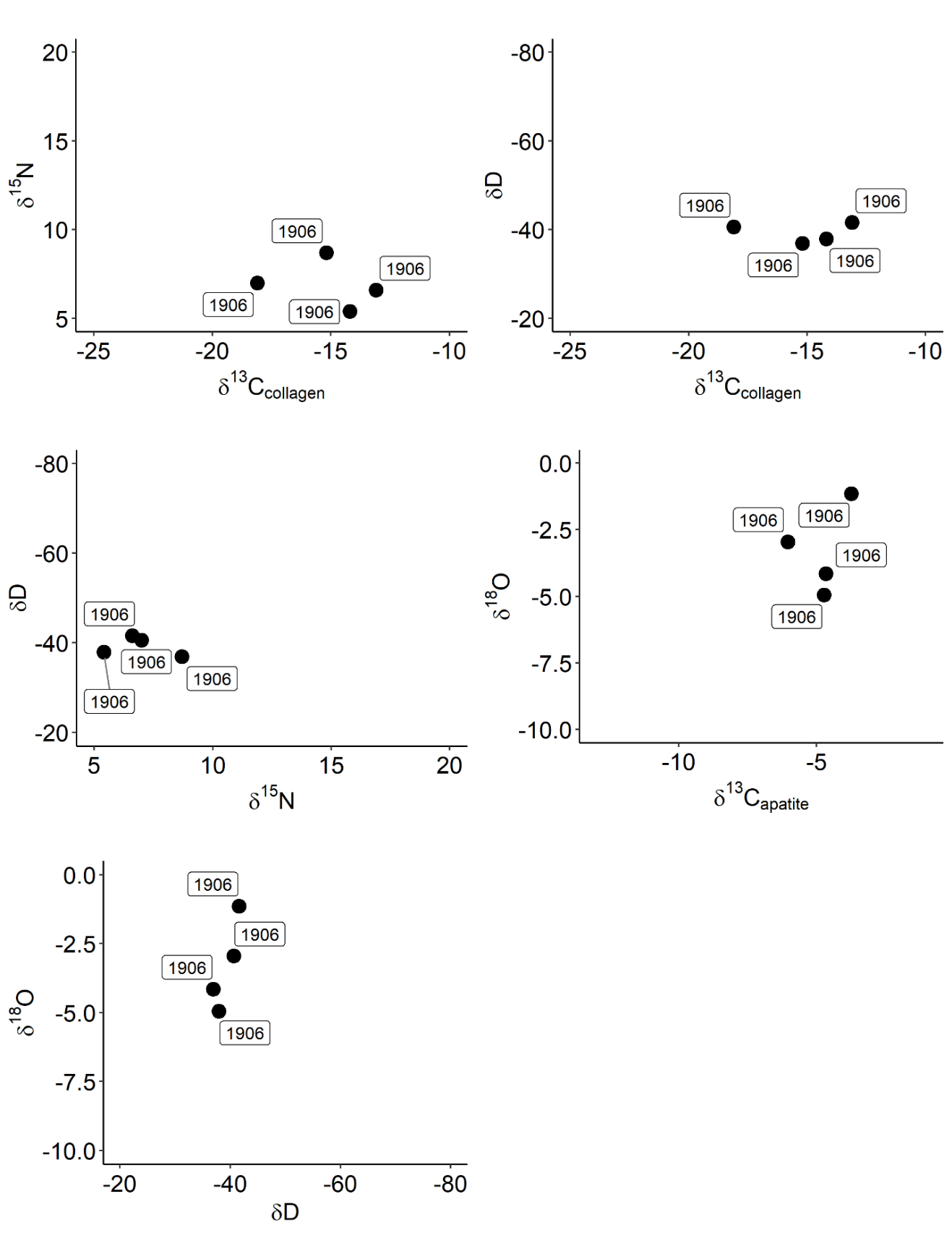


Fig. S24. Diachronic trends in *C. n. guentheri* from Isabela Island.


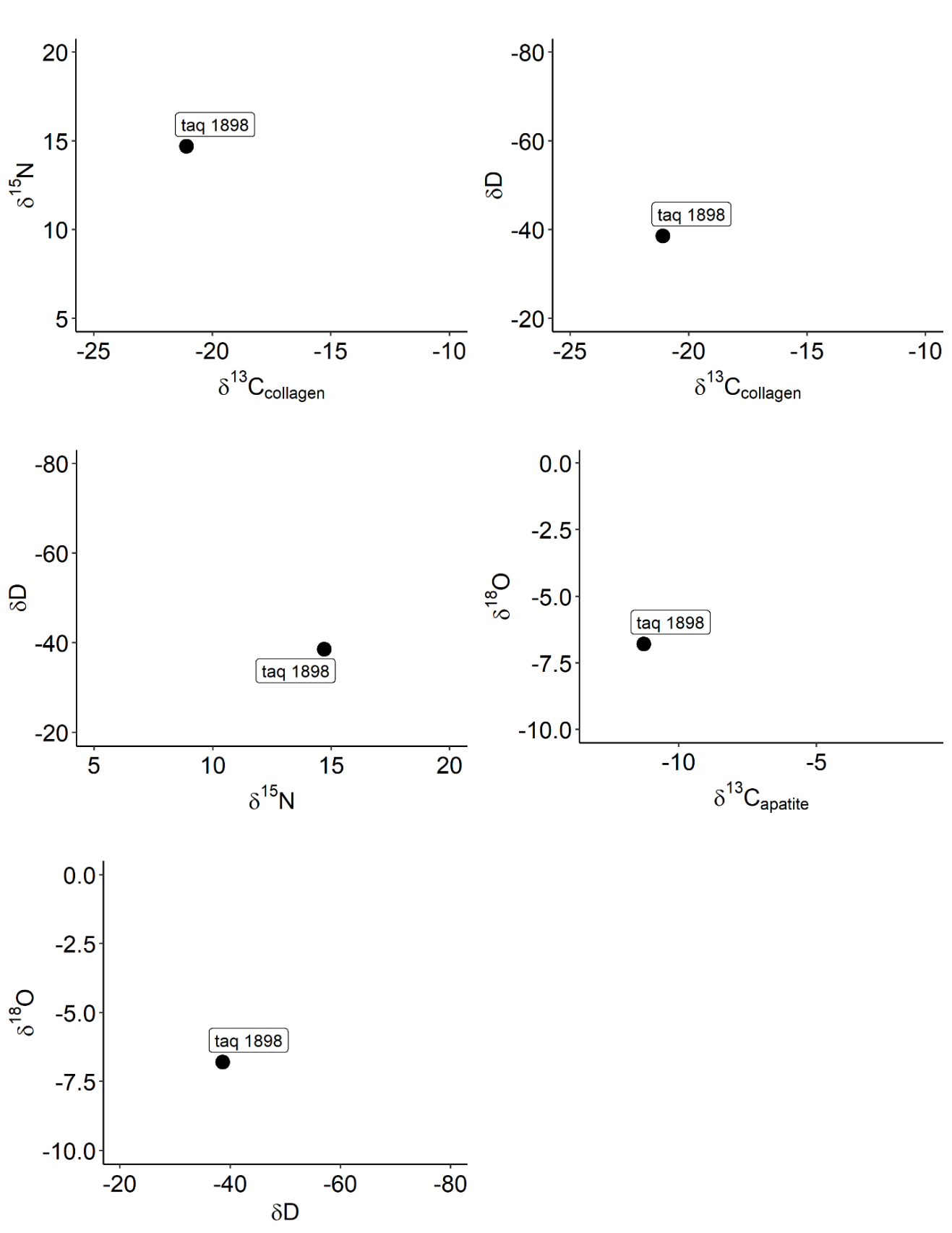


Fig. S25. Diachronic trends in *C. n. microphyes* from Isabela Island.


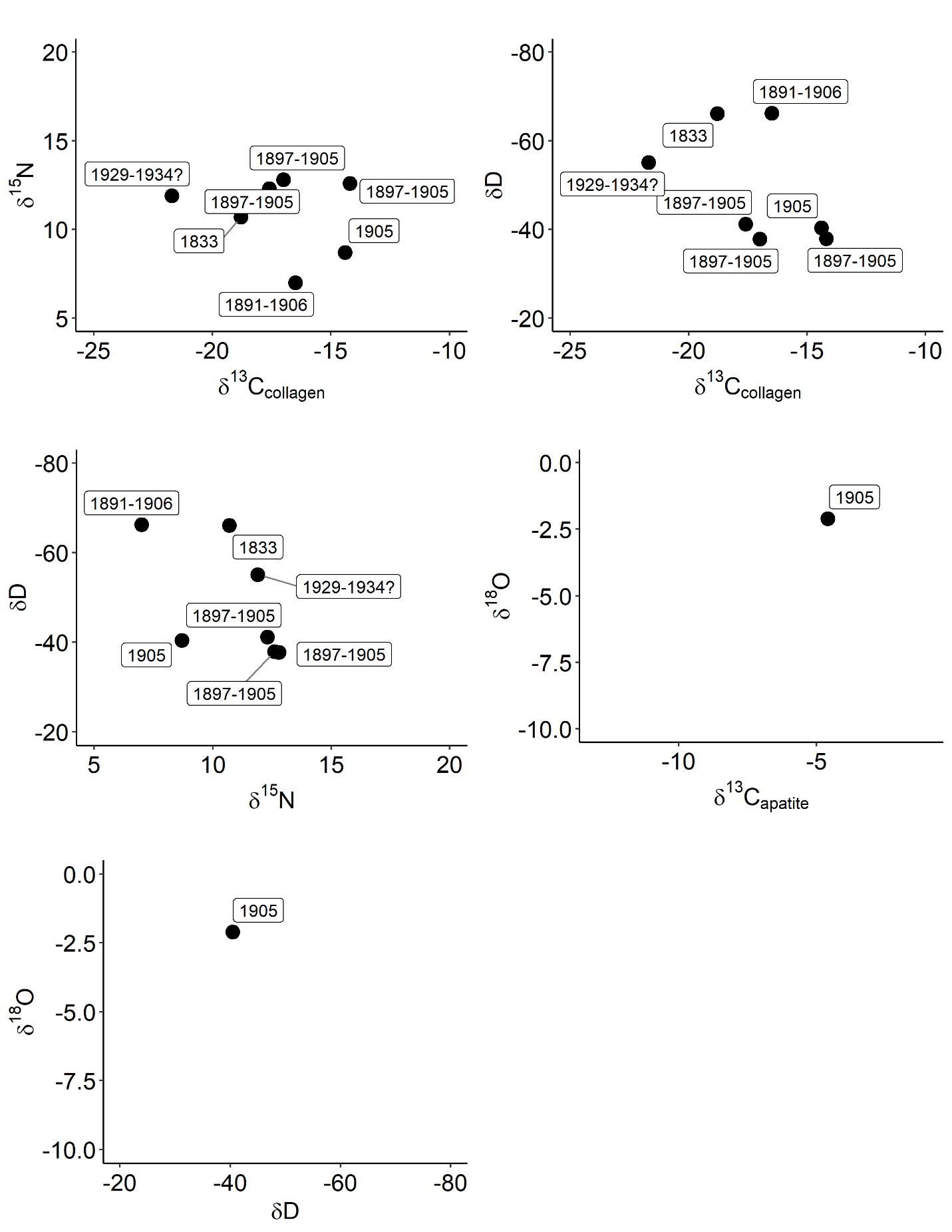


Fig. S26. Diachronic trends in *C. n. porteri* from Santa Cruz Island.


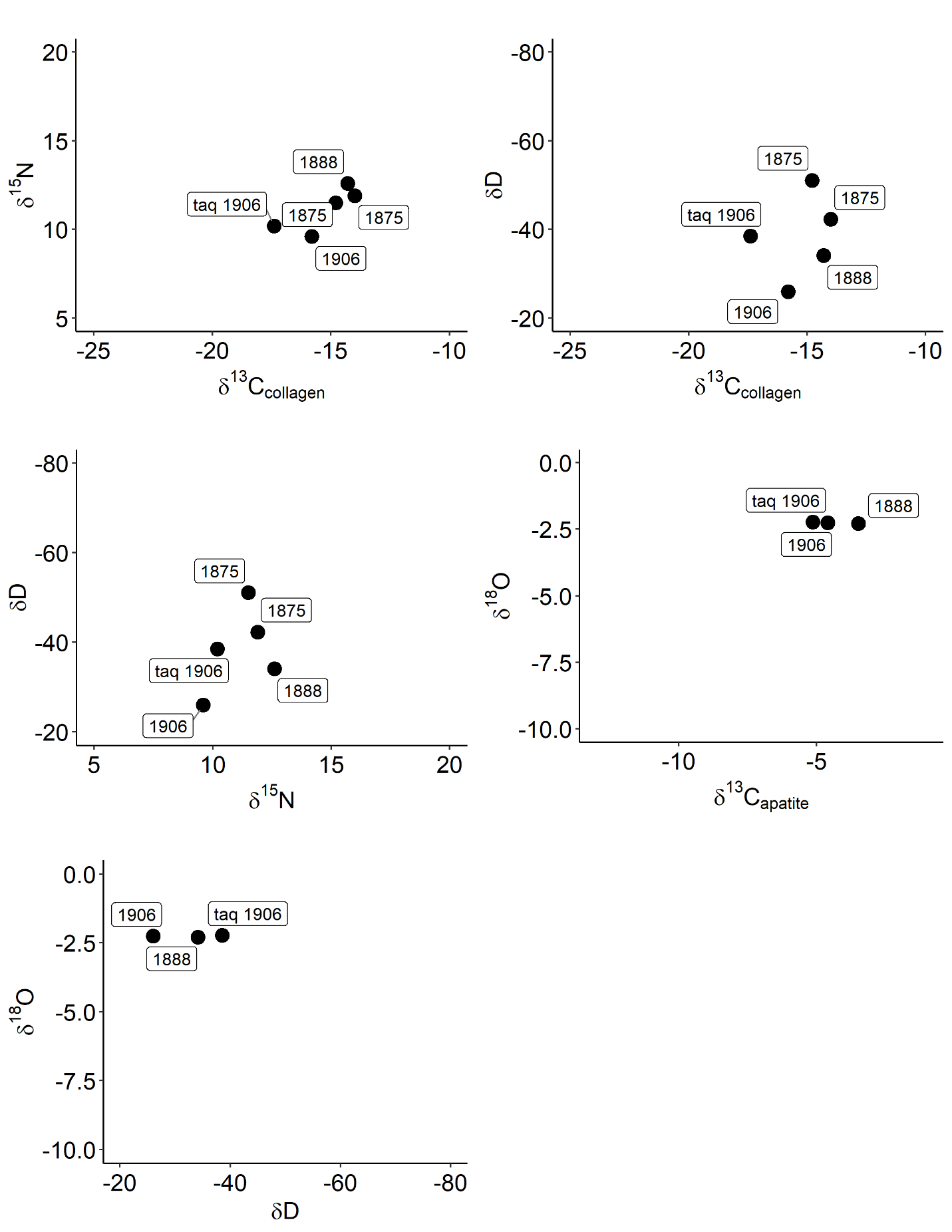


Fig. S27. Diachronic trends in *C. n. abingdonii* from Pinta Island.


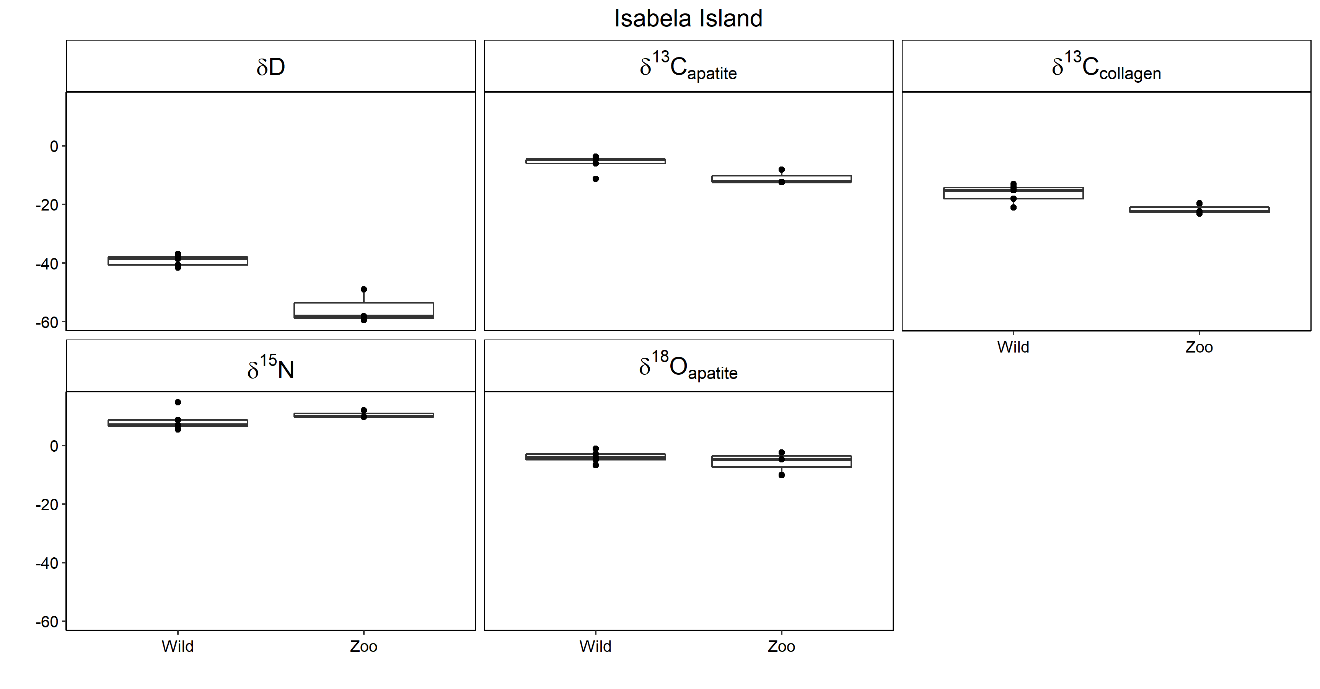


Fig. S28. Captive versus wild diets in Isabela Island tortoises. In several instances, our analysis of Galapagos tortoises included individuals transported from the islands, kept in captivity, fed a non-native diet, died, and then became incorporated into museum collections. In these specimens, it was possible to examine captive-fed diets against native, wild tortoise diets in the Galapagos, for the same species. Multiple tortoise species from Isabela Island, and tortoises from Pinzón and Santa Cruz (see below), have consistency in dietary stable isotopes whether or not they consumed captive diets or consumed native vegetation in the Galapagos. Captive-fed tortoises from Isabela have slightly more depleted δD, but these tortoises include *C. n. becki* and *C. n. vicina*, while the wild-fed tortoises from Isabela include *C. n. microphyes* and *C. n. guentheri*.


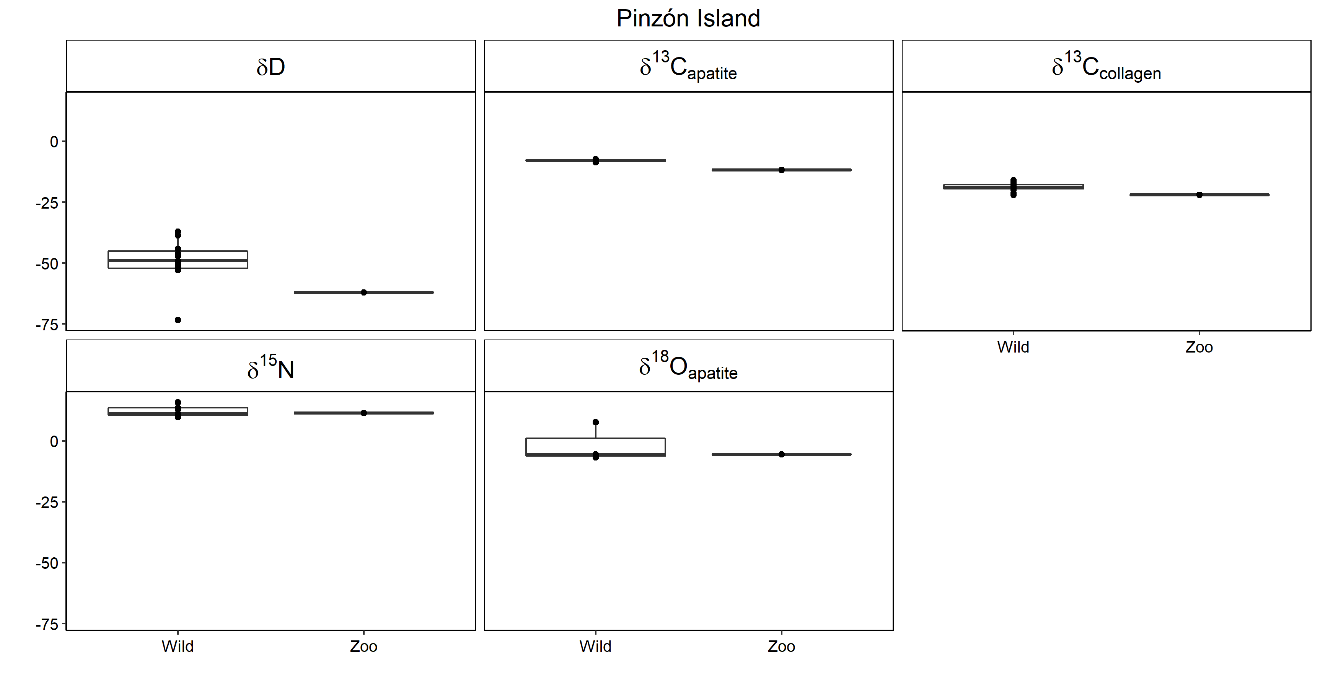


Fig. S29. Captive versus wild diets in Pinzón Island tortoises. See caption for Figure S27.


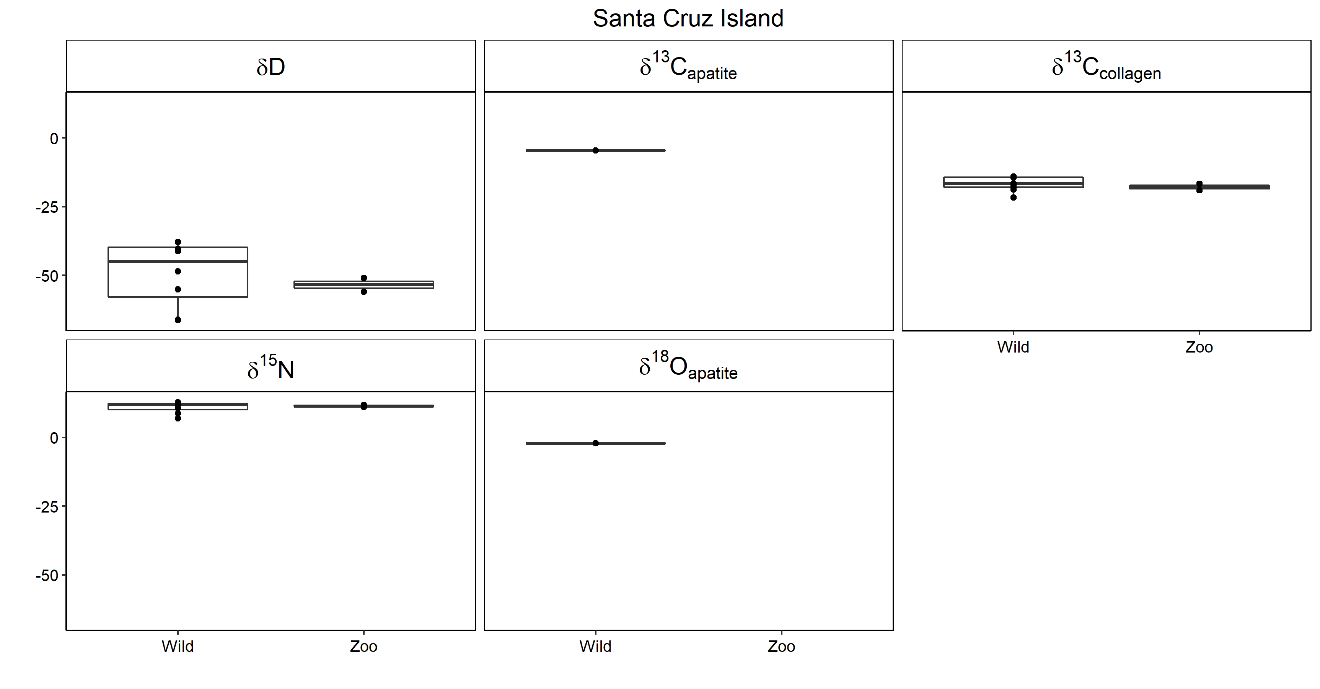


Fig. S30. Captive versus wild diets in Santa Cruz Island tortoises. See caption for Figure S27.

**Tables**

**Table S1. Galápagos Tortoise Stable Isotope Data.**

See attached dataset spreadsheet.

**Table S2. Modern Plant Stable Isotope Data**.

Gibbs et al. [34] collected and analyzed all modern plant stable isotope samples from Pinta, Espanola and Santa Fe islands. Sample preparation and analysis occurred at the State University of New York, Syracuse, College of Environmental Science and Forestry.

| Island | Date of Collection | Identifier | Taxon | %N | %C | δ^13^C | δ^15^N |
| --- | --- | --- | --- | --- | --- | --- | --- |
| Pinta | 10/3/2011 | GAL-P-Alte-3 | Alte | 1.9173852 | 41.2708717 | -30.837 | 7.2 |
| Pinta | 10/3/2011 | GAL-P-Alte-2 | Alte | 1.4498662 | 40.4224417 | -30.602 | 7 |
| Pinta | 10/3/2011 | GAL-P-Alte-1 | Alte | 2.1925163 | 41.5300357 | -30.321 | 8.8 |
| Pinta | 10/3/2011 | GAL-P-Aris-1 | Aris | 2.1948891 | 39.6511379 | -12.556 | 5.9 |
| Pinta | 10/3/2011 | GAL-P-Aris-2 | Aris | 1.5837215 | 40.0435795 | -12.453 | -0.8 |
| Pinta | 10/3/2011 | GAL-P-Aris-3 | Aris | 1.6216566 | 39.1918258 | -12.211 | 5.9 |
| Espanola | 8/27/2010 | Bursera 1 | Bursera | 1.5736982 | 46.4559861 | -29.1891 | 9.8 |
| Espanola | 8/27/2010 | Bursera 2 | Bursera | 1.653893 | 43.7607473 | -29.1487 | 12.8 |
| Espanola | 8/27/2010 | Bursera 3 | Bursera | 1.6588442 | 44.0703787 | -29.01235 | 4.2 |
| Pinta | 10/3/2011 | GAL-P-Burs-1 | Bursera | 1.1390013 | 41.7362202 | -28.915 | 4.2 |
| Santa Fe | 10/1/2011 | GAL-SF-Burs-1 | Bursera | 1.4299967 | 41.181478 | -28.705 | NA |
| Espanola | 8/27/2010 | Cordia 3 | Cordia | 1.3294242 | 43.1194017 | -30.08295 | 4.1 |
| Espanola | 8/27/2010 | Cordia 2 | Cordia | 1.421708 | 43.5859463 | -29.91226 | 3.5 |
| Espanola | 8/27/2010 | Cordia 1 | Cordia | 1.4347976 | 42.1036575 | -29.72743 | 8.8 |
| Santa Fe | 10/1/2011 | GAL-SF-Cord-1 | Cordia | 1.0507469 | 40.8369736 | -29.022 | 4.3 |
| Pinta | 10/3/2011 | GAL-P-Crot-2 | Croton | 1.7010772 | 40.891256 | -28.699 | 3.2 |
| Espanola | 8/27/2010 | Croton 2 | Croton | 1.6811526 | 41.9172229 | -28.26192 | 2.7 |
| Espanola | 8/27/2010 | Croton 3 | Croton | 1.6488733 | 41.7889825 | -28.24879 | 5.5 |
| Pinta | 10/3/2011 | GAL-P-Crot-3 | Croton | 1.7267828 | 40.6087639 | -28.134 | 5.6 |
| Pinta | 10/3/2011 | GAL-P-Crot-1 | Croton | 1.4622687 | 41.0423557 | -28.104 | 1.9 |
| Espanola | 8/27/2010 | Croton 1 | Croton | 1.7679889 | 39.4086432 | -28.08113 | NA |
| Santa Fe | 10/1/2011 | GAL-SF-Crot-1 | Croton | 0.8146954 | 36.5341222 | -27.946 | 3.8 |
| Espanola | 8/27/2010 | Cyperus 1 | Cypernus | NA | 43.6531713 | -12.39684 | -1.4 |
| Espanola | 8/27/2010 | Cyperus 2 | Cypernus | NA | 41.6235057 | -12.32917 | 2 |
| Espanola | 8/27/2010 | Cyperus 3 | Cypernus | NA | 42.7276431 | -12.24837 | 2.8 |
| Santa Fe | 10/1/2011 | GAL-SF-Erag-1 | Erag | 0.7957383 | 38.2216803 | -13.729 | 9.7 |
| Pinta | 10/3/2011 | GAL-P-Just-1 | Just | 1.4352182 | 40.8768068 | -28.614 | 4.3 |
| Pinta | 10/3/2011 | GAL-P-Just-3 | Just | 1.4833783 | 43.0103093 | -28.052 | NA |
| Pinta | 10/3/2011 | GAL-P-Just-2 | Just | 2.1907399 | 42.9823049 | -27.047 | NA |
| Pinta | 10/3/2011 | GAL-P-Lant-3 | Lant | 1.5829634 | 43.6240689 | -30.06 | 5.1 |
| Pinta | 10/3/2011 | GAL-P-Lant-2 | Lant | 1.9114 | 42.4257803 | -29.749 | 8.4 |
| Pinta | 10/3/2011 | GAL-P-Lant-1 | Lant | 1.8478548 | 42.3208425 | -29.588 | 7.9 |
| Santa Fe | 10/1/2011 | GAL-SF-Lept-1 | Lept | 0.4567937 | 39.3034094 | -13.274 | 5.6 |
| Espanola | 8/27/2010 | Opuntia 1 | Opuntia | NA | 52.7212199 | -15.95911 | NA |
| Espanola | 8/27/2010 | Opuntia 2 | Opuntia | NA | 52.1104693 | -15.44199 | 7.6 |
| Espanola | 8/27/2010 | Opuntia 3 | Opuntia | NA | 50.4405454 | -14.9279 | 1.2 |
| Pinta | 10/3/2011 | GAL-P-Opun-2 | Opuntia | 0.5137235 | 35.0430556 | -12.954 | 5.6 |
| Pinta | 10/3/2011 | GAL-P-Opun-1 | Opuntia | 0.384148 | 28.0997727 | -12.497 | -1.6 |
| Pinta | 10/3/2011 | GAL-P-Opun-3 | Opuntia | 0.623656 | 31.2101105 | -12.25 | 2.8 |
| Santa Fe | 10/1/2011 | GAL-SF-Opun-1 | Opuntia | NA | NA | NA | 3 |
| Espanola | 8/27/2010 | Parkinsonia 3 | Parkinsonia | 2.044652 | 46.2157258 | -27.96599 | NA |
| Espanola | 8/27/2010 | Parkinsonia 1 | Parkinsonia | 2.1700271 | 48.0370202 | -27.77106 | 5.8 |
| Espanola | 8/27/2010 | Parkinsonia 2 | Parkinsonia | 1.8762422 | 46.4324487 | -27.42059 | 3.9 |
| Espanola | 8/27/2010 | Paspalum 2 | Paspalum | NA | 42.1519957 | -14.65722 | 2.2 |
| Espanola | 8/27/2010 | Paspalum 1 | Paspalum | 7.0827349 | 267.525664 | -14.54713 | 0 |
| Espanola | 8/27/2010 | Paspalum 3 | Paspalum | 1.1029752 | 39.8854031 | -14.34311 | 9.7 |
| Pinta | 10/3/2011 | GAL-P-Pasp-3 | Paspalum | 0.6941678 | 39.320516 | -12.063 | 2.4 |
| Pinta | 10/3/2011 | GAL-P-Pasp-1 | Paspalum | 0.9008516 | 40.1336142 | -11.952 | 6.1 |
| Pinta | 10/3/2011 | GAL-P-Pasp-2 | Paspalum | 0.8651688 | 41.0860045 | -11.885 | 7 |
| Pinta | 10/3/2011 | GAL-P-Piso-3 | Piso | 2.5947451 | 38.9364972 | -29.998 | 5.6 |
| Pinta | 10/3/2011 | GAL-P-Piso-1 | Piso | 1.9738789 | 40.0642345 | -29.715 | 8.8 |
| Pinta | 10/3/2011 | GAL-P-Piso-2 | Piso | 2.8019312 | 40.8741518 | -28.551 | 3.7 |
| Espanola | 8/27/2010 | Prosopis 2 | Prosopis | 3.1094784 | 49.2263647 | -27.03477 | 9.1 |
| Espanola | 8/27/2010 | Prosopis 3 | Prosopis | 3.2881665 | 45.6325706 | -26.86913 | 10.8 |
| Espanola | 8/27/2010 | Prosopis 1 | Prosopis | 3.0780754 | 46.9037264 | -26.76611 | 8.2 |
| Pinta | 10/3/2011 | GAL-P-Rhyn-1 | Rhyn | 2.8535603 | 44.8483187 | -29.286 | 6.2 |
| Pinta | 10/3/2011 | GAL-P-Rhyn-2 | Rhyn | 3.5588042 | 44.8893899 | -29.285 | 12.9 |
| Pinta | 10/3/2011 | GAL-P-Rhyn-3 | Rhyn | 2.6261455 | 44.1832179 | -28.626 | 4.1 |
| Santa Fe | 10/1/2011 | GAL-SF-Tric-1 | Tric | 0.4681967 | 37.2064837 | -13.796 | 8.3 |
| Pinta | 10/3/2011 | GAL-P-Walt-2 | Walt | 1.9781781 | 46.5479591 | -29.001 | 4.6 |
| Pinta | 10/3/2011 | GAL-P-Walt-1 | Walt | 1.8295751 | 45.9625741 | -28.776 | NA |
| Santa Fe | 10/1/2011 | GAL-SF-Walt-1 | Walt | 0.5590074 | 44.2836437 | -28.724 | NA |
| Pinta | 10/3/2011 | GAL-P-Walt-3 | Walt | 1.4071468 | 46.0534931 | -27.039 | 10.4 |
| Pinta | 10/3/2011 | GAL-P-Zant-1 | Zant | 2.0849774 | 37.2234321 | -30.308 | 4.3 |
| Pinta | 10/3/2011 | GAL-P-Zant-3 | Zant | 1.8833689 | 35.8700966 | -30.149 | 4.2 |
| Pinta | 10/3/2011 | GAL-P-Zant-2 | Zant | 1.7287273 | 37.9375862 | -29.92 | 5 |

**SI References**

1. Turtle Taxonomy Working Group, “Turtles of the World: Annotated Checklist and Atlas of Taxonomy, Synonym, Distribution, and Conservation Status, 9th Edition” (Chelonian Research Foundation and Turtle Conservancy 2021).

2. A. G. Pastron, K. Bruner, “Archaeological Resources Report for the 717 Battery Street Project, City and County of San Francisco, California.” Available from the Northwest Information Center, Sonoma, CA (2014).

3. C. Conrad, K. W. Gobalet, K. Bruner, A. G. Pastron, Hide, Tallow and Terrapin: Gold Rush-Era Zooarchaeology at Thompson’s Cove (CA-SFR-186H), San Francisco, California. *Int. Hist. Arch*. **19**, 502-551 (2015).

4. T. H. Fritts, P. R. Fritts, “Race with Extinction Herpetological Notes of J.R. Slevin’s Journey to the Galápagos 1905-1906.” Herp. Mono. (1982).

5. N. Poulakakis, M. Russello, D. Geist, A. Caccone, Unravelling the peculiarities of island life: vicariance, dispersal and the diversification of the extinct and extant giant Galápagos tortoises. *Mol. Eco*. **21**, 160-173 (2012).

6. E. Heller, Papers from the Hopkins Stanford Galápagos Expedition, 1898-1899. *Wash. Acad. of Sci.* **5**, 39-98 (1903).

7. E. L. Jensen, M. C. Quinzin, J. M. Miller, M. A. Russello, R. C. Garrick, D. L. Edwards, S. Glaberman, Y. Chiari, N. Poulakakis, W. Tapia, J. P. Gibbs, A. Caccone, A new lineage of Galapagos giant tortoises identified from museum samples. *Heredity* **128**, 261-270 (2022).

8. C. H. Townsend, Propagation of the Giant Tortoise in the United States. *Sci.* **68**, 30 (1928).

9. C. H. Townsend, The Galápagos Islands Revisited. *Bull. New York Zoo. Soc.* **31**, 148-169 (1928).

10. J. R. Slevin, “The Galápagos Islands A History of Their Exploration” (California Academy of Sciences 1959).

11. M. M. Stewart, J. C. Mitchell, Historical Perspectives: Herndon Glenn Dowling, Jr. *Copeia* **1**, 166-172 (2013).

12. H. G. Dowling, Vanishing Giants & Enduring Dwarfs The Tortoises. *Ani. King.* 66-75 (1961).

13. H. G. Dowling, Recent Report on Galápagos Tortoises: Some Survive. *Herp. Rev.* **2**, 3 (1970).

14. C. H. Townsend, Impending Extinction of the Galápagos Tortoises. *Zool. Soc. Bull.* **27**, 55-56 (1924).

15. C. H. Townsend, The Whaler and the Tortoise. *Sci. Mon.* **21**, 166-172 (1925).

16. A. Agassiz, Reports on the Dredging Operations off the West Coast of Central America to the Galápagos, etc., by the U.S. Fish Commission Steamer “Albatross.” II. General Sketch of the Expedition of the “Albatross,” from February to May, 1891. *Bull. Mus. Comp. Zoo. Harv. Coll. Cam.* **13**, 1-90 (1892).

17. Z. L. Tanner, “Report on the Work of the United States Fish Commission Steamer Albatross from January 1, 1887, to June 30, 1888” (Washington, DC 1890).

18. Smithsonian Institution Archives, “Personnel Records, 1892-1952.” Available at https://sirismm.si.edu/EADpdfs/SIA.FA05-123.pdf. (n.d.).

19. C. H. Townsend, Giant Tortoises. *Sci. Amer.* **144**, 42-44 (1931).

20. G. Pinchot, “To the South Seas; the Cruise of the Schooner Mary Pinchot to the Galápagos, the Marquesas, and the Tuamotu Islands, and Tahiti” (Blue Ribbon Books 1930).

21. K. Roosevelt, The Mountain Party on Indefatigable Island. *New York Zoo. Soc.* **18**, 156-163 (1930).

22. H. L. Clark, The Hancock Pacific Expedition. *Sci. Mon.* **47**, 511-518 (1938).

23. C. M. Fraser, “General Account of the Scientific Work of the Velero III in the Eastern Pacific, 1931-41” (The University of Southern California Press 1943).

24. R. C. Bell, L. A. Scheinberg, “The collectors: Beginnings of scientific inquiry and the lasting impacts of living and museum collections”, in Galapagos Giant Tortoises, J.P. Gibbs, L.J. Cayot, W.T. Aguilera, Eds. (Elsevier, 2020), pp. 97-114.

25. C. Conrad and J. P. Gibbs, “The era of exploitation: 1535-1959” in Galapagos Giant Tortoises, J.P. Gibbs, L.J. Cayot, W.T. Aguilera, Eds. (Elsevier, 2020), pp. 63-82.

26. W. Cookson, On the Tortoises Etc. of Galápagos Islands. *Proc. Zoo. Soc.* (1876).

27. A. Günther, Account of the Zoological Collection made during the visit of H.M.S. ‘Peterel’ to the Galápagos Islands. *Proc. Zoo. Soc. Lon.* **64** (1877).

28. J. Van Denburgh, “Expedition of the California Academy of Sciences to the Galápagos Islands, 1905-1906” (California Academy of Sciences 1914).

29. W. Rothschild, The Giant Land Tortoises of the Galápagos Island in the Tring Museum. *Nov. Zoo.* **12**, 403-417 (1915).

30. A. Günther, Testudo Galapagoensis. *Nov. Zoo.* **9**, 184-192 (1902).

31. Anon, Donations, *Bos. J. Nat. His.* **1**, 521 (1834-37).

32. J. B. S. Jackson, Anatomical Description of the Galápagos Tortoise. *J. Bos. Soc. Nat. Hist.* **1**, 443-464 (1837).

33. W. Rothschild, Description of a New Species of Gigantic Land Tortoise from Indefatigable Island. *Nov. Zoo.* **10**, 119 (1903).

34. J. P. Gibbs, E. A. Hunter, K. T. Shoemaker, W. A. Tapia, L. J. Cayot, Demographic Outcomes and Ecosystems Implications of Giant Tortoise Reintroduction to Española Island, Galapagos. *PLoS ONE* **9**(10), e110742 (2014).
